# A diastereoselective approach to axially chiral biaryls via electrochemically enabled cyclization cascade

**DOI:** 10.3762/bjoc.15.76

**Published:** 2019-03-28

**Authors:** Hong Yan, Zhong-Yi Mao, Zhong-Wei Hou, Jinshuai Song, Hai-Chao Xu

**Affiliations:** 1 State Key Laboratory of Physical Chemistry of Solid Surfaces, Key Laboratory of Chemical Biology of Fujian Province, iChEM and College of Chemistry and Chemical Engineering, Xiamen University, People’s Republic of China; 2 College of Chemistry and Molecular Engineering, Zhengzhou University, Zhengzhou 450001, People’s Republic of China

**Keywords:** axial chirality, biaryl, electrochemistry, oxidation, radical

## Abstract

A diastereoselective approach to axially chiral imidazopyridine-containing biaryls has been developed. The reactions proceed through a radical cyclization cascade to construct the biaryls with good to excellent central-to-axial chirality transfer.

## Introduction

Axially chiral biaryls are prevalent in natural products, bioactive molecules and organocatalysts [1–2]. Among the many methods that have been developed for the synthesis of chiral biaryls [3–10], reactions that take avantage of the central-to-axial chirality transfer have been less explored [11–14]. In addition, an antroposelective synthesis of imidazopyridine-based biaryls has not been reported.

**Scheme 1:**
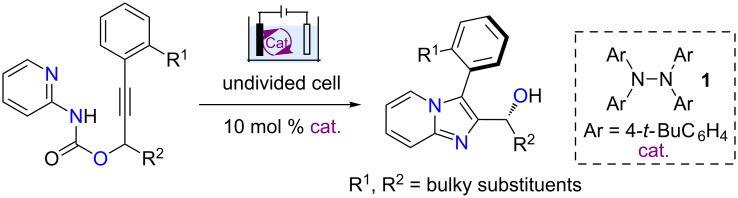
Reaction design.

Nitrogen-centered radicals (NCRs) are attractive reactive intermediates for organic synthesis as they provide opportunities for the efficient construction of C–N bonds [15–19]. Recently, the generation of NCRs through electron transfer-based methods has been attracting attention. Organic electrochemistry is a powerful tool for adding or taking electrons from organic molecules to promote redox reactions because of its reagent-free feature and the tunability of electric current and potential [20–30]. We [31–34] and others [35–41] have studied the reactions of electrochemically generated NCRs. Particularly, we have recently reported an electrochemical synthesis of imidazo-fused N-heteroaromatic compounds via a radical cyclization cascade [31]. Building on this work, we report herein an atroposelective synthesis of imidazopyridine-containing biaryls via central-to-axial chirality transfer (Scheme 1).

## Results and Discussion

The substituents on the phenyl ring (R^1^) and at the propargylic position (R^2^) of carbamate **2** were varied to study their effects on the diastereoselectivity (Table 1). The electrolysis was conducted under previously established conditions employing a three-necked round-bottomed flask as the cell, a reticulated vitreous carbon (RVC) anode and a platinum plate cathode [31]. The reaction was carried in refluxing MeCN/H_2_O (9:1) with tetraarylhydrazine **1** as the redox catalyst, NaHCO_3_ (2 equiv) as an additive, and Et_4_NBF_4_ (1 equiv) as the supporting electrolyte. These investigations indicated that bulky tertiary groups at both R^1^ and R^2^ positions were needed to ensure efficient chirality transfer. Hence, carbamate **2a** (Table 1, entry 1) bearing a *t*-Bu group at R^1^ and R^2^ positions, respectively, reacted to give imidazopyridine-based biaryl **3a** in 68% yield with good diastereoselectivity (14:1 dr). Replacing the *t-*Bu group at the propargylic position with iPr (Table 1, entry 2) or on the phenyl ring with Ph (Table 1, entry 3) or OiPr (Table 1, entry 4) all resulted in low diastereoselectivity (2:1 to 3:1).

**Table 1:** Investigation on the effects of substituents on the diastereoselectivity.^a^

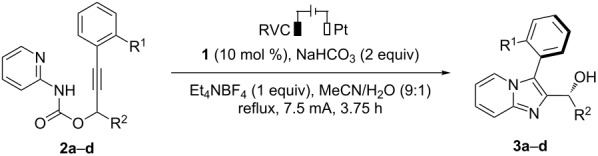		

Entry	Substrate	Product, yield,^b^ dr^c^

1	**2a** (R^1^ *= t-*Bu, R^2^ = *t-*Bu)	(±)-**3a**, 68%, 14:1 dr
2	**2b** (R^1^ *= t-*Bu, R^2^ = iPr)	(±)-**3b**, 64%, 3:1 dr
3	**2c** (R^1^ = Ph, R^2^ = *t-*Bu)	(±)-**3c**, 73%, 2:1 dr
4	**2d** (R^1^ *=* OiPr, R^2^ = *t-*Bu)	(±)-**3d**, 78%, 3:1 dr

^a^Reaction conditions: undivided cell, **1** (0.03 mmol), **2** (0.3 mmol), H_2_O (1 mL), MeCN (9 mL), 3.5 F mol^−1^. ^b^Isolated yield. ^c^Determined by ^1^H NMR analysis of the crude reaction mixture.

**Scheme 2:**
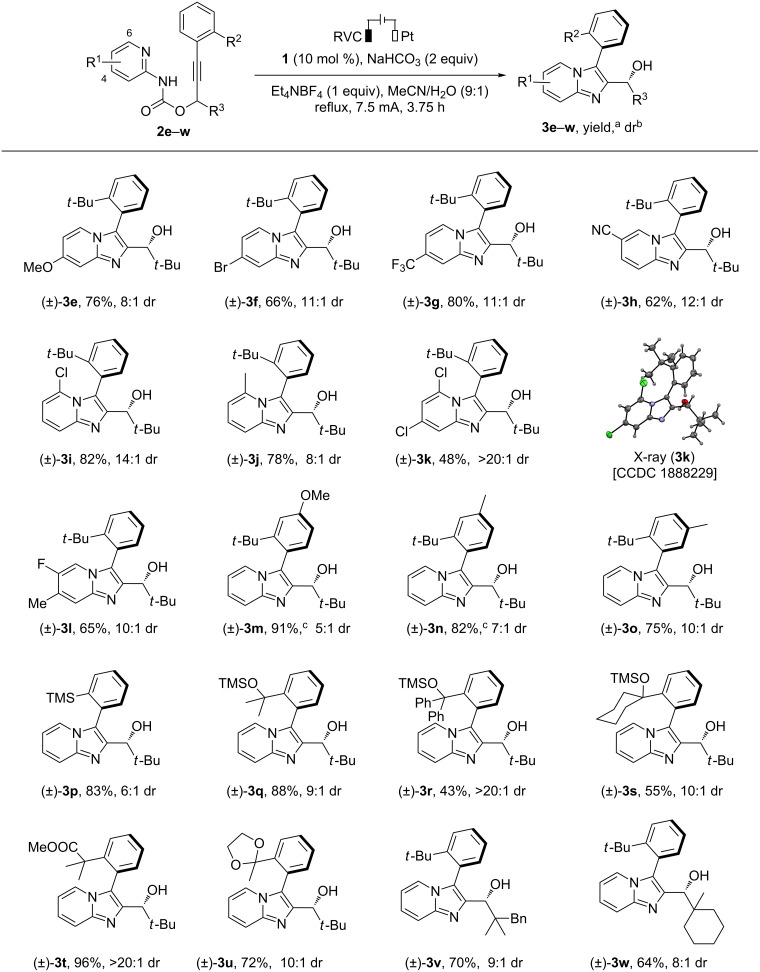
Scope of electrochemical synthesis of axially chiral biaryls. Reaction conditions: undivided cell, **2** (0.3 mmol), H_2_O (1 mL), MeCN (9 mL), 3.5 F mol^−1^. ^a^Isolated yield of the major diastereomer. ^b^Determined by ^1^H NMR analysis of crude reaction mixture. ^c^Combined yield of the two diastereomers.

The scope of the electrosynthesis was investigated by varying the peripheral substituents of the carbamate substrate **2** (Scheme 2). The pyridyl ring could be substituted at positions 4, 5 and 6 with a range of substituents with diverse electronic properties such as OMe (**3e**), Br (**3f**), CF_3_ (**3g**), CN (**3h**), Cl (**3i**), and Me (**3j**). Pyridyl rings bearing multiple substituents were also tolerated (**3k** and **3l**). The stereochemistry of the biaryl product was determined by obtaining an X-ray crystal structure of **3k**. The *t-*Bu-substituted phenyl ring on the alkyne moiety containing an extra OMe (**3m**) or Me (**3n** and **3o**) group was tolerated albeit with reduced diastereoselectivity. The *t-*Bu group on the phenyl ring and at the propargylic position could be replaced with other bulky tertiary substituents to afford a range of functionalized biaryl products (**3p**–**w**). The electrochemical conditions were compatible with several functional groups including aryltrimethylsilane (**3p**), silyl ether (**3q**–**s**), ester (**3t**) and cylic ketal (**3u**). Note that all the diastereomers were separable by flash column chromatography.

Heating a solution of the major isomer of **3n** in MeCN at 80 °C for 4 h did not lead to isomerization, suggesting that the stereoselectivity of the reaction was not controlled by relative thermodynamic stability of the diastereomers. The major isomer of **3c**, which contained a sterically less demanding Ph group at the R^1^ position (cf. Table 1), did not isomerize at room temperature for 1 year. However, heating a solution of this compound in MeCN at 80 °C for 4 h resulted in a mixture of diastereomers in a ratio of 3:1. These results suggest that the sterically demanding substituents at R^1^ and R^2^ positions (cf. Table 1) are critical to ensure good stereoselectivity during the product formation and to prevent isomerization after the reaction.

**Scheme 3:**
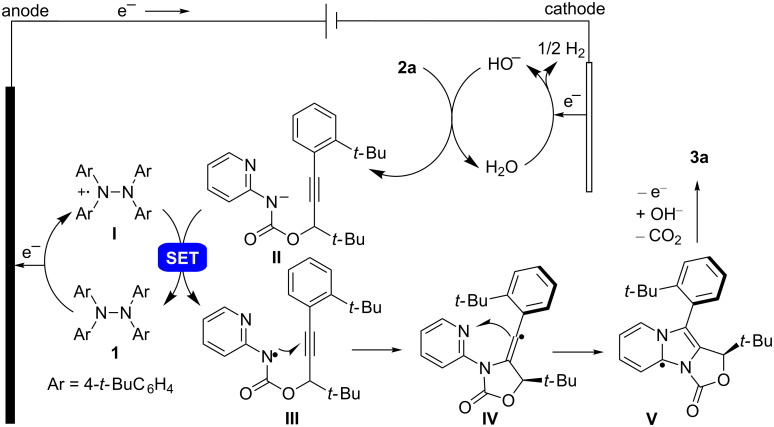
Proposed reaction mechanism for the electrochemical synthesis of **3a**.

A mechanism for the electrochemical synthesis was proposed based on the results from our previous work [31] and of this work (Scheme 3). The redox catalyst **1** is oxidized at the anode to give radical cation **I**. In the meanwhile, H_2_O is reduced at the cathode to afford HO^−^ and H_2_. The base generated at the cathode deprotonates **2a** to give its conjugate base **II**. The anionic **II** is oxidized by radical cation **I** through single electron transfer (SET) to give radical intermediate **III**, which undergoes a biscyclization to give **V**. Further oxidation of **V** followed by hydrolysis of the cyclic carbamate moiety leads to the formation of **3a**.

**Scheme 4:**
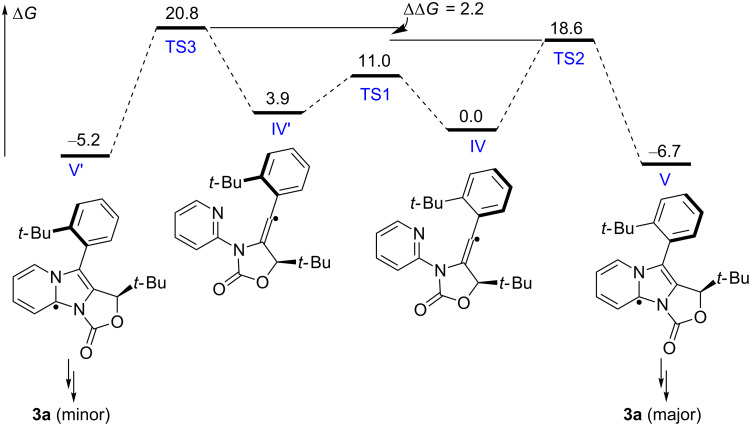
Computation investigation on the vinyl radical cyclization. DFT (M06-2X/6-31G*) calculated energetics (kcal mol^−1^) are Gibbs free energies in MeCN.

Based on the proposed reaction mechanism and the results mentioned above, the cyclization of vinyl radical **IV** to give **V** is the atroposelective step. Density functional theory (DFT)-based calculations suggested that the cyclization of **IV** could be explained by a Curtin–Hammett scenario (Scheme 4) [42]. Specifically, the equilibrium of the conformations **IV** and **IV′** is much faster than their respectively cyclizations to give **V** and **V′**. Since **TS2** is relatively lower in energy than **TS3**, **V** is formed as the major product.

## Conclusion

In summary, we have developed a diastereoselective approach for the synthesis of axially chiral biaryls through an electricity-powered cyclization cascade. The reactions employ easily assembled starting materials and afford functionalized imidazopyridine-based biaryls in good to high yields and diastereoselectivity.

## Supporting Information

File 1 Experimental part.
