# Supplementary material for: A diastereoselective approach to axially chiral biaryls via electrochemically enabled cyclization cascade
Source: Beilstein J Org Chem. 2019 Mar 28;15:795–800. doi: 10.3762/bjoc.15.76 (PMC6444422; doi:10.3762/bjoc.15.76)
Supplement: File 1 — Experimental part. [file Beilstein_J_Org_Chem-15-795-s001.pdf]

BEILSTEIN JOURNAL OF ORGANIC CHEMISTRY

Supporting Information

for

A diastereoselective approach to axially chiral biaryls via electrochemically enabled cyclization cascade

Hong Yan, Zhong-Yi Mao, Zhong-Wei Hou, Jinshuai Song and Hai-Chao Xu

Beilstein J. Org. Chem. **2019,** *15,* 795–800. doi:10.3762/bjoc.15.76

Experimental part

License and Terms: This is a supporting information file under the terms of the Creative Commons Attribution License (http://creativecommons.org/licenses/by/4.0). Please note that the reuse, redistribution and reproduction in particular requires that the authors and source are credited. The license is subject to the *Beilstein Journal of Organic Chemistry* terms and conditions: (https://www.beilstein-journals.org/bjoc)

Contents **Page**

Contents	Page
1. General Information	S2
2. General Procedure for the Electrolysis	S2
3. Unsuccessful Substrates	S2
4. Characterization Data for the Electrolysis Products	S2
6. General Procedure for the Synthesis of Substrates	S10
6. X-ray Crystallography of 3k	S18
7. References	S19
8. NMR Spectra for New Compounds	S20

1. General Information

Acetonitrile were obtained by distillation under argon from calcium hydride. Flash column chromatography was performed with silica gel (230–400 mesh). NMR spectra were recorded on Bruker AV-400, Bruker AV-500 and Bruker Ascend 600 A instruments. Data were reported as chemical shifts in ppm relative to $CDCl_3$ (7.27 ppm) for ^1H and $CDCl_3$ (77.2 ppm), respectively. The abbreviations used for explaining the multiplicities were as follows: s = singlet, d = doublet, t = triplet, q = quartet, m = multiplet, br = broad. High resolution mass spectra (ESI HRMS) were recorded on a Micromass QTOF2 Quadruple/Time-of-Flight Tandem mass spectrometer by the instrumentation center of Department of Chemistry, Xiamen University. Cyclic voltammograms were obtained on a CHI 760E potentiostat. Infrared spectra (IR) were recorded on a Nicolet AVATER FTIR330 spectrometer.

2. General Procedure for the Electrolysis

All the electrolysis reactions were performed according to a previously reported procedure [1]. A 25 mL three-necked round-bottomed flask was charged with **1** (0.03 mmol), the substrate (0.3 mmol), Et_4NBF_4 (0.3 mmol), and $NaHCO_3$ (0.6 mmol). The flask was equipped with a condenser, a reticulated vitreous carbon (100 PPI, ~65 cm^2 cm^{-3}, 1 cm x 1 cm x 1.2 cm) anode and a platinum plate (1 cm x 1 cm) cathode and then flushed with argon. MeCN (9 mL) and H_2O (1 mL) were added. The electrolysis was carried out at 80 °C (oil bath temperature) using a constant current of 7.5 mA for 3.75 h. The reaction mixture was cooled to RT and concentrated under reduced pressure. The residue was chromatographed through silica gel eluting with ethyl acetate/hexanes to give the desired product.

3. Unsuccessful Substrates

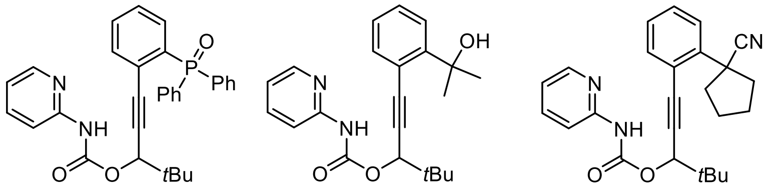

4. Characterization Data for the Electrolysis Products

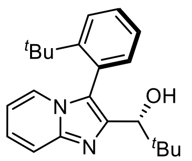

(±)-1-(3-(2-(*tert*-Butyl)phenyl)imidazo[1,2-*a*]pyridin-2-yl)-2,2-dimethylpropan-1-ol (3a). Yield = 68%; White solid; ^1H NMR (500 MHz, $CDCl_3$) δ 7.69 (dd, *J* = 8.2, 1.3 Hz, 1H), 7.66–7.60 (m, 1H), 7.51–7.44 (m, 1H), 7.41–7.36 (m, 1H), 7.31–7.26 (m, 1H), 7.21–7.12 (m, 1H), 7.06 (dd, *J* = 7.5, 1.6 Hz, 1H), 6.74–6.67 (m, 1H), 4.18 (d, *J* = 8.4 Hz, 1H), 2.86 (d, *J* = 8.9 Hz, 1H), 1.10 (s, 9H), 0.97 (s, 9H); ^{13}C NMR (126 MHz, $CDCl_3$) δ 152.4, 144.7, 143.8, 135.5, 130.0, 128.4, 126.2, 126.1, 124.3, 124.2, 123.4, 117.3, 112.1, 75.4, 37.1, 36.6, 31.6,

26.6. IR (neat, cm^{-1}):3294, 2957, 1633, 1504, 1477, 1373, 1261, 1067, 1016, 792, 756, 753. ESI HRMS *m/z* $(M+H)^+$ calcd 337.2274, obsd 337.2281.

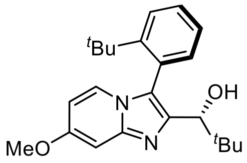

(±)-1-(3-(2-(*tert*-Butyl)phenyl)-7-methoxyimidazo[1,2-*a*]pyridin-2-yl)-2,2-dimethylpropan-1-ol (3e). Yield = 76%; White solid; ^1H NMR (500 MHz, $CDCl_3$) δ 7.66 (dd, *J* = 8.2, 1.3 Hz, 1H), 7.48–7.41 (m, 1H), 7.30–7.23 (m, 1H), 7.19 (d, *J* = 7.4 Hz, 1H), 7.06 (dd, *J* = 7.6, 1.6 Hz, 1H), 6.91 (d, *J* = 2.4 Hz, 1H), 6.42 (dd, *J* = 7.5, 2.4 Hz, 1H), 4.11 (d, *J* = 8.5 Hz, 1H), 3.86 (s, 3H), 2.81 (d, *J* = 8.9 Hz, 1H), 1.12 (s, 9H), 0.97 (s, 9H); ^{13}C NMR (126 MHz, $CDCl_3$) δ 157.7, 152.5, 145.1, 143.8, 135.8, 129.9, 128.3, 126.3, 126.0, 124.8, 122.1, 107.1, 94.5, 75.4, 55.6, 37.0, 36.6, 31.6, 26.7. IR (neat, cm^{-1}): 3354, 2953, 1648, 1476, 1364, 1220, 1172, 1085, 1026; ESI HRMS *m/z* $(M+Na)^+$ calcd 391.2356, obsd 391.2352.

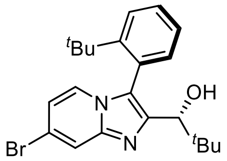

(±)-1-(7-Bromo-3-(2-(*tert*-butyl)phenyl)imidazo[1,2-*a*]pyridin-2-yl)-2,2-dimethylpropan-1-ol (3f). Yield = 66%; White solid; ^1H NMR (400 MHz, $CDCl_3$) δ 7.69 (dd, *J* = 8.2, 1.3 Hz, 1H), 7.66 (dd, *J* = 2.1, 0.8 Hz, 1H), 7.51–7.45 (m, 1H), 7.33–7.27 (m, 2H), 7.04 (dd, *J* = 7.6, 1.7 Hz, 1H), 6.69 (dd, *J* = 7.3, 2.0 Hz, 1H), 4.16 (d, *J* = 6.3 Hz, 1H), 3.03 (s, 1H), 1.10 (s, 9H), 0.96 (s, 9H). ^{13}C NMR (101 MHz, $CDCl_3$) δ 152.4, 145.7, 143.5, 135.3, 130.6, 130.2, 128.5, 126.2, 125.6, 124.5, 123.6, 116.3, 113.8, 75.3, 37.0, 36.6, 31.6, 26.6. IR (neat, cm^{-1}): 3350, 2958, 2905, 1629, 1508, 1480, 1392, 1370, 1340, 1062, 1001, 920, 877, 782, 755. ESI HRMS *m/z* $(M+Na)^+$ calcd 437.1199, obsd 437.1203.

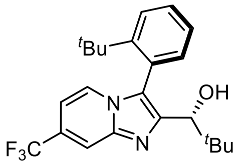

(±)-1-(3-(2-(*tert*-Butyl)phenyl)-7-(trifluoromethyl)imidazo[1,2-*a*]pyridin-2-yl)-2,2-dimethylpropan-1-ol (3g). Yield = 80%; White solid; ^1H NMR (500 MHz, $CDCl_3$) δ 8.01 (s, 1H), 7.71 (d, *J* = 8.2 Hz, 1H), 7.54–7.48 (m, 2H), 7.34–7.27 (m, 1H), 7.04 (d, *J* = 7.5 Hz, 1H), 6.88 (d, *J* = 7.2 Hz, 1H), 4.21 (d, *J* = 7.5 Hz, 1H), 3.17 (s, 1H), 1.10 (s, 9H), 0.96 (s, 9H); ^{13}C NMR (214 MHz, $CDCl_3$) δ 152.3, 147.3, 141.9, 135.0, 130.4, 128.6, 126.3, 125.9 (q, *J* = 33.7 Hz), 125.2, 125.0, 124.9, 123.7 (q, *J* = 271.9 Hz), 115.6 (q, *J* = 4.9 Hz), 108.1 (q, *J* = 3.0 Hz), 75.3, 37.1, 36.6, 31.6, 26.5; ^{19}F NMR (471 MHz, $CDCl_3$) δ -63.3. IR (neat, cm^{-1}): 3386, 2955, 1506, 1480, 1349, 1340, 1236, 1171, 1130, 1052, 1002, 921, 763; ESI HRMS *m/z* $(M+Na)^+$ calcd 427.1968, obsd 429.1967.

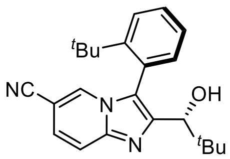

(±)-3-(2-(*tert*-Butyl)phenyl)-2-(1-hydroxy-2,2-dimethylpropyl)imidazo[1,2-*a*]pyridine-6-carbonitrile (3h). Yield = 62%; White solid; ^{1}H NMR (400 MHz, $CDCl_3$) δ 7.80 (dd, J = 1.8, 1.0 Hz, 1H), 7.77–7.68 (m, 2H), 7.56–7.51 (m, 1H), 7.37–7.29 (m, 1H), 7.27 (dd, J = 9.4, 1.7 Hz, 1H), 7.03 (dd, J = 7.6, 1.6 Hz, 1H), 4.21 (d, J = 8.9 Hz, 1H), 2.90 (s, 1H), 1.09 (s, 9H), 0.96 (s, 9H). ^{13}C NMR (101 MHz, $CDCl_3$) δ 152.4, 147.4, 143.0, 135.0, 130.8, 130.5, 128.8, 126.5, 124.8, 124.3, 123.9, 118.5, 116.8, 98.5, 75.2, 37.1, 36.6, 31.6, 26.5. IR (neat, cm^{-1}): 3401, 2955, 2230, 1713, 1631, 1481, 1380, 1321, 1272, 1062, 1002, 763. ESI HRMS *m/z* $(M+Na)^+$ calcd 384.2046, obsd 384.2047.

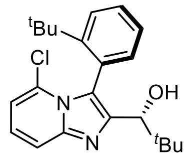

(±)-1-(3-(2-(*tert*-Butyl)phenyl)-5-chloroimidazo[1,2-*a*]pyridin-2-yl)-2,2-dimethylpropan-1-ol (3i). Yield = 82%; White solid; ^{1}H NMR (600 MHz, $CDCl_3$) δ 7.60 (dd, J = 15.9, 8.6 Hz, 2H), 7.45–7.39 (m, 1H), 7.17 (dd, J = 18.8, 7.4 Hz, 2H), 7.09 (d, J = 8.0 Hz, 1H), 6.74 (d, J = 7.1 Hz, 1H), 4.04 (d, J = 8.3 Hz, 1H), 2.76 (d, J = 9.2 Hz, 1H), 1.10 (s, 9H), 0.97 (s, 9H); ^{13}C NMR (151 MHz, $CDCl_3$) δ 151.5, 146.0, 145.6, 136.3, 129.4, 127.8, 127.2, 126.7, 124.8, 123.8, 123.7, 116.3, 113.9, 74.4, 36.8, 36.8, 31.8, 26.5. IR (neat, cm^{-1}): 3351, 2955, 1624, 1488, 1393, 1363, 1275, 1152, 1064, 1015, 775, 761; ESI HRMS *m/z* $(M+Na)^+$ calcd 393.1704, obsd 393.1709.

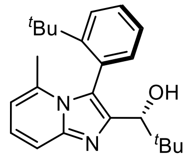

(±)-1-(3-(2-(*tert*-Butyl)phenyl)-5-methylimidazo[1,2-*a*]pyridin-2-yl)-2,2-dimethylpropan-1-ol (3j). Yield = 78%; White solid; ^{1}H NMR (500 MHz, $CDCl_3$) δ 7.59 (d, J = 8.1 Hz, 1H), 7.52 (d, J = 9.0 Hz, 1H), 7.47–7.40 (m, 1H), 7.29–7.23 (m, 1H), 7.19–7.12 (m, 1H), 7.05 (dd, J = 9.0, 6.8 Hz, 1H), 6.43 (d, J = 6.7 Hz, 1H), 4.01 (d, J = 9.1 Hz, 1H), 2.70 (d, J = 9.0 Hz, 1H), 2.00 (s, 3H), 1.07 (s, 9H), 0.97 (s, 9H); ^{13}C NMR (126 MHz, $CDCl_3$) δ 152.1, 145.4, 145.4, 136.6, 136.4, 129.7, 128.5, 128.3, 124.2, 124.1, 123.9, 116.0, 113.5, 74.8, 37.1, 37.0, 32.1, 26.7, 21.2; IR (neat, cm^{-1}): 3195, 2964, 1736, 1582, 1532, 1460, 1395, 1262, 1211, 1076, 985, 757; ESI HRMS *m/z* $(M+Na)^+$ calcd 375.2407, obsd 375.2371.

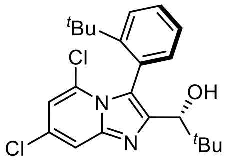

(±)-1-(3-(2-(*tert*-Butyl)phenyl)-5,7-dichloroimidazo[1,2-*a*]pyridin-2-yl)-2,2-dimethylpropan-1-ol (3k). Yield = 48%; White solid; ^{1}H NMR (400 MHz, $CDCl_3$) δ 7.62 (d, J = 2.0 Hz, 1H), 7.61–7.57 (m, 1H), 7.47–7.40 (m, 1H), 7.19–7.13 (m, 2H), 6.77 (d, J = 2.0 Hz, 1H), 4.02 (d, J = 9.3 Hz, 1H), 2.61 (d, J = 9.3 Hz, 1H), 1.10 (s, 9H), 0.96 (s, 9H); ^{13}C NMR (101 MHz, $CDCl_3$) δ 151.8, 147.1, 145.2, 136.5, 130.1, 129.9, 128.2, 127.5, 126.8, 125.4, 124.1, 115.5, 115.1, 74.6, 37.0, 37.0, 32.1, 26.6; IR (neat, cm^{-1}): 3362, 2955, 1618, 1491, 1365, 1274, 1148, 1061, 1011, 828, 762; ESI HRMS *m/z* $(M+Na)^+$ calcd 427.1314, obsd 427.1318.

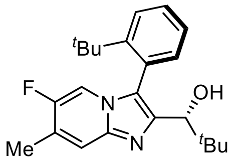

(±)-1-(3-(2-(*tert*-Butyl)phenyl)-6-fluoro-7-methylimidazo[1,2-*a*]pyridin-2-yl)-2,2-dimethylpropan-1-ol (3l). Yield = 65%; White solid; ^{1}H NMR (400 MHz, $CDCl_3$) δ 7.69 (dd, J = 8.2, 1.1 Hz, 1H), 7.52–7.44 (m, 1H), 7.42 (d, J = 7.1 Hz, 1H), 7.29-7.24 (m, 1H), 7.24 (d, J = 4.7 Hz, 1H), 7.03 (dd, J = 7.6, 1.6 Hz, 1H), 4.15 (d, J = 8.9 Hz, 1H), 2.89 (s, 1H), 2.36 (s, 3H), 1.11 (s, 9H), 0.95 (s, 9H). ^{13}C NMR (126 MHz, $CDCl_3$) δ 153.0 (d, J = 243.2 Hz), 152.2, 145.3 (d, J = 2.1 Hz), 141.6, 135.2, 129.9, 128.3, 126.4 (d, J = 21.2 Hz), 126.0, 125.8, 123.7 (d, J = 1.9 Hz), 116.7 (d, J = 5.6 Hz), 109.9 (d, J = 42.3 Hz), 75.2, 36.9, 36.5, 31.4, 26.4, 15.2 (d, J = 3.1 Hz). ^{19}F NMR (471 MHz, $CDCl_3$) δ -144.1; IR (neat, cm^{-1}): 3359, 2921, 1632, 1513, 1382, 1123, 1063; ESI HRMS *m/z* $(M+Na)^+$ calcd 391.2156, obsd 391.2158.

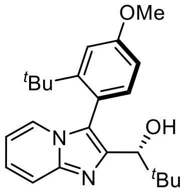

(±)-1-(3-(2-(*tert*-Butyl)-4-methoxyphenyl)imidazo[1,2-*a*]pyridin-2-yl)-2,2-dimethylpropan-1-ol (3m). Major isomer: Yield = 75%; White solid; ^{1}H NMR (400 MHz, $CDCl_3$) δ 7.60 (d, J = 9.1 Hz, 1H), 7.39 (d, J = 6.8 Hz, 1H), 7.21 (d, J = 2.5 Hz, 1H), 7.16–7.10 (m, 1H), 6.98 (d, J = 8.4 Hz, 1H), 6.82 (dd, J = 8.4, 2.6 Hz, 1H), 6.70–6.65 (m, 1H), 4.19 (d, J = 8.3 Hz, 1H), 3.88 (s, 3H), 2.89 (s, 1H), 1.08 (s, 9H), 0.97 (s, 9H); ^{13}C NMR (101 MHz, $CDCl_3$) δ 160.5, 154.1, 145.0, 143.8, 136.8, 124.3, 124.0, 123.1, 118.2, 117.3, 115.0, 112.0, 110.5, 75.4, 55.3, 37.0, 36.6, 31.4, 26.6; IR (neat, cm^{-1}): 3317, 2953, 1605, 1561, 1505, 1480, 1372, 1268, 1230, 1060, 1015, 761; ESI HRMS *m/z* $(M+Na)^+$ calcd 389.2199, obsd 389.2201.

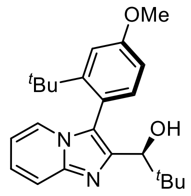

(±)-1-(3-(2-(*tert*-Butyl)-4-methoxyphenyl)imidazo[1,2-*a*]pyridin-2-yl)-2,2-dimethylpropan-1-ol (3m'). Minor isomer: Yield = 16%; White solid; ^{1}H NMR (400 MHz, $CDCl_3$) δ 7.61–7.58 (m, 1H), 7.46–7.44 (m, 1H), 7.22 (d, J = 2.6 Hz, 1H), 7.17–7.11 (m, 1H), 7.07 (d, J = 8.5 Hz, 1H), 6.84 (dd, J = 8.5, 2.7 Hz, 1H), 6.72–6.69 (m, 1H), 4.46 (s, 1H), 3.88 (s, 3H), 1.13 (s, 9H), 1.09 (s, 9H). ^{13}C NMR (151 MHz, $CDCl_3$) δ 160.5, 153.2, 145.1, 143.5, 136.2, 124.6, 124.2, 124.0, 117.9, 117.5, 116.3, 112.0, 111.3, 76.3, 55.4, 37.2, 36.9, 31.8, 26.9. IR (neat, cm^{-1}): 3431, 2955, 1604, 1480, 1342, 1289, 1230, 1048, 1012, 753; ESI HRMS *m/z* $(M+Na)^+$ calcd 389.2199, obsd 389.2203.

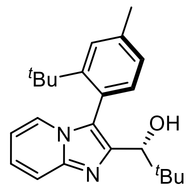

(±)-1-(3-(2-(*tert*-Butyl)-4-methylphenyl)imidazo[1,2-*a*]pyridin-2-yl)-2,2-dimethylpropan-1-ol (3e). Major isomer. Yield = 71%; White solid; ^{1}H NMR (400 MHz, $CDCl_3$) δ 7.61 (d, J = 9.0 Hz, 1H), 7.47 (s, 1H), 7.38 (d, J = 6.9 Hz, 1H), 7.17–7.05 (m, 2H), 6.94 (d, J = 7.7 Hz, 1H), 6.70–6.65 (m, 1H), 4.19 (d, J = 8.5 Hz, 1H), 2.90 (d, J = 8.8 Hz, 1H), 2.44 (s, 3H), 1.08 (s, 9H), 0.97 (s, 9H). ^{13}C NMR (101 MHz, $CDCl_3$) δ 152.0, 144.8, 143.8, 139.6, 135.4, 129.1, 126.9, 124.3, 123.9, 123.4, 123.1, 117.3, 111.9, 75.4, 37.0, 36.4, 31.6, 26.6, 21.8. IR (neat, cm^{-1}): 3320, 2950, 1504, 1476, 1387, 1371, 1353, 1275, 1058, 1014, 760; ESI HRMS *m/z* $(M+Na)^+$ calcd 373.2250, obsd 373.2247.

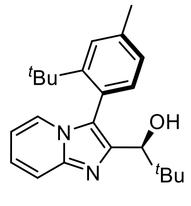

(±)-1-(3-(2-(*tert*-Butyl)-4-methylphenyl)imidazo[1,2-*a*]pyridin-2-yl)-2,2-dimethylpropan-1-ol (3e'). Minor isomer. Yield = 11%; White solid; ^{1}H NMR (400 MHz, $CDCl_3$) δ 7.62–7.59 (m, 1H), 7.48 (d, J = 1.8 Hz, 1H), 7.46–7.43 (m, 1H), 7.17 – 7.12 (m, 1H), 7.10 (dd, J = 7.7, 1.1 Hz, 1H), 7.01 (d, J = 7.7 Hz, 1H), 6.70–6.67 (m, 1H), 4.47 (s, 1H), 2.44 (s, 3H), 1.14 (s, 9H), 1.09 (s, 9H); ^{13}C NMR (126 MHz, $CDCl_3$) δ 151.0, 144.8, 143.3, 139.3, 134.6, 130.5, 127.4, 124.7, 124.0, 123.7, 122.5, 117.3, 111.8, 76.2, 36.8, 36.8, 31.7, 26.7, 21.6; IR (neat, cm^{-1}): 3412, 2954, 1607, 1504, 1478, 1362, 1342, 1267, 1014, 753; ESI HRMS *m/z* $(M+Na)^+$

calcd 373.2250, obsd 373.2250.

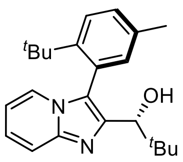

(±)-1-(3-(2-(*tert*-Butyl)-5-methylphenyl)imidazo[1,2-*a*]pyridin-2-yl)-2,2-dimethylpropan-1-ol (3o). Yield = 75%; White solid; ^{1}H NMR (400 MHz, $CDCl_3$) δ 7.66–7.58 (m, 1H), 7.56 (d, *J* = 8.3 Hz, 1H), 7.44–7.36 (m, 1H), 7.27 (dd, *J* = 8.3, 2.1 Hz, 1H), 7.19–7.10 (m, 1H), 6.87 (d, *J* = 2.1 Hz, 1H), 6.73–6.65 (m, 1H), 4.19 (d, *J* = 8.5 Hz, 1H), 2.86 (s, 1H), 2.30 (s, 3H), 1.07 (s, 9H), 0.98 (s, 9H); ^{13}C NMR (101 MHz, $CDCl_3$) δ 149.2, 144.7, 143.8, 136.0, 135.5, 130.6, 128.3, 126.0, 124.4, 124.0, 123.5, 117.3, 112.0, 75.4, 37.1, 36.3, 31.7, 26.6, 20.5; IR (neat, cm^{-1}): 3294, 2951, 1506, 1496, 1384, 1345, 1289, 1275, 1054, 1015, 826, 744; ESI HRMS *m/z* $(M+Na)^+$ calcd 373.2250, obsd 373.2248.

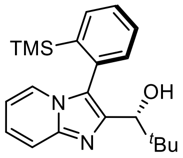

(±)-2,2-Dimethyl-1-(3-(2-(trimethylsilyl)phenyl)imidazo[1,2-*a*]pyridin-2-yl)propan-1-ol (3p). Yield = 83%; White solid; ^{1}H NMR (500 MHz, $CDCl_3$) δ 7.80–7.72 (m, 1H), 7.63 (d, *J* = 9.0 Hz, 1H), 7.54–7.46 (m, 2H), 7.44 (d, *J* = 6.8 Hz, 1H), 7.29–7.23 (m, 1H), 7.21–7.13 (m, 1H), 6.73–6.65 (m, 1H), 4.16 (d, *J* = 8.7 Hz, 1H), 3.01 (d, *J* = 8.7 Hz, 1H), 0.94 (s, 9H), –0.14 (s, 9H); ^{13}C NMR (126 MHz, CDCl3) δ 144.8, 143.7, 143.7, 136.0, 133.8, 133.3, 129.3, 128.9, 124.3, 124.1, 123.2, 117.4, 112.1, 75.0, 37.0, 26.5, –0.6; IR (neat, cm^{-1}): 3369, 2951, 1505, 1362, 1342, 1250, 1121, 1082, 1014, 839, 752; ESI HRMS *m/z* $(M+Na)^+$ calcd 377.2020, obsd 377.2012.

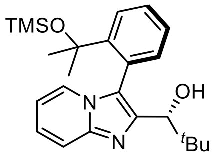

(±)-2,2-Dimethyl-1-(3-(2-(2-((trimethylsilyl)oxy)propan-2-yl)phenyl)imidazo[1,2-*a*]pyridin-2-yl)propan-1-ol (3q). Yield = 88%; White solid; ^{1}H NMR (500 MHz, $CDCl_3$) δ 7.90 (d, *J* = 8.1 Hz, 1H), 7.62 (d, *J* = 9.1 Hz, 1H), 7.54–7.46 (m, 1H), 7.39 (d, *J* = 6.9 Hz, 1H), 7.34–7.27 (m, 1H), 7.18–7.11 (m, 1H), 7.05 (dd, *J* = 7.5 Hz, 1.0 Hz, 1H), 6.72–6.63 (m, 1H), 4.12 (d, *J* = 8.8 Hz, 1H), 2.92 (s, 1H), 1.56 (s, 3H), 1.11 (s, 3H), 0.96 (s, 9H), -0.07 (s, 9H); ^{13}C NMR (126 MHz, $CDCl_3$) δ 152.0, 144.3, 143.9, 134.9, 129.9, 127.9, 126.8, 124.5, 124.5, 124.1, 123.0, 117.4, 112.0, 76.7, 75.2, 36.9, 32.7, 30.7, 26.6, 2.5; IR (neat, cm^{-1}): 3367, 2953, 1506, 1477, 1362, 1250, 1163, 1016, 908, 840, 753; ESI HRMS *m/z* $(M+Na)^+$ calcd 433.2282,

obsd 433.2282.

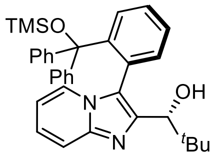

(±)-1-(3-(2-(Diphenyl((trimethylsilyl)oxy)methyl)phenyl)imidazo[1,2-*a*]pyridin-2-yl)-2,2-dimethylpropan-1-ol (3r). Yield = 43%; White solid; ^{1}H NMR (400 MHz, $CDCl_3$) δ 8.27 (d, *J* = 9.1 Hz, 1H), 8.20–8.11 (m, 2H), 8.08–8.03 (m, 1H), 8.02–7.94 (m, 4H), 7.94–7.85 (m, 5H), 7.83–7.74 (m, 4H), 7.30–7.22 (m, 1H), 3.83 (d, *J* = 5.8 Hz, 1H), 2.33 (d, *J* = 5.9 Hz, 1H), 1.46 (s, 9H), 0.15 (s, 9H); ^{13}C NMR (101 MHz, $CDCl_3$) δ 150.3, 144.4, 144.0, 143.6, 136.0, 131.0, 130.2, 128.6, 128.2, 128.1, 127.9, 127.8, 127.7, 127.2, 127.0, 124.6, 123.4, 123.0, 117.3, 111.4, 86.6, 74.5, 36.6, 26.6, 1.7; IR (neat, cm^{-1}): 3590, 3508, 2951, 1505, 1492, 1361, 1251, 1165, 1071, 1009, 904, 840, 880, 703; ESI HRMS *m/z* $(M+Na)^+$ calcd 557.2595, obsd 557.2601.

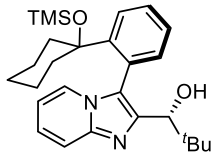

(±)-2,2-Dimethyl-1-(3-(2-(1-((trimethylsilyl)oxy)cyclohexyl)phenyl)imidazo[1,2-*a*]pyridin -2-yl)propan-1-ol (3s). Yield = 55%; White solid; ^{1}H NMR (400 MHz, $CDCl_3$) δ 7.99 (dd, *J* = 8.3, 1.4 Hz, 1H), 7.64 (d, *J* = 9.1 Hz, 1H), 7.57–7.48 (m, 1H), 7.45 (d, *J* = 6.9 Hz, 1H), 7.37–7.29 (m, 1H), 7.23–7.13 (m, 1H), 7.08 (dd, *J* = 7.5, 1.5 Hz, 1H), 6.76–6.68 (m, 1H), 4.14 (d, *J* = 8.7 Hz, 1H), 2.68 (d, *J* = 8.7 Hz, 1H), 1.85–1.74 (m, 1H), 1.70–1.45 (m, 2H), 1.45–1.23 (m, 4H), 1.16–1.03 (m, 2H), 0.97 (s, 9H), 0.93–0.83 (m, 1H), -0.02 (s, 9H); ^{13}C NMR (151 MHz, $CDCl_3$) δ 152.0, 144.6, 143.8, 134.5, 130.6, 129.8, 127.0, 124.2, 124.0, 123.2, 117.5, 112.2, 79.0, 75.1, 41.1, 36.9, 35.9, 26.6, 25.3, 22.8, 22.6, 2.6; IR (neat, cm^{-1}): 3340, 2948, 1500, 1474, 1345, 1248, 1143, 1017, 839, 753; ESI HRMS *m/z* $(M+Na)^+$ calcd 473.2595, obsd 473.2604.

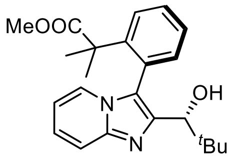

(±)-Methyl 2-(2-(2-(1-hydroxy-2,2-dimethylpropyl)imidazo[1,2-*a*]pyridin-3-yl)phenyl)-2-methylpropanoate (3t). Yield = 96%; White solid; ^{1}H NMR (600 MHz, $CDCl_3$) δ 7.66–7.58 (m, 2H), 7.56–7.48 (m, 1H), 7.40 (d, *J* = 6.7 Hz, 1H), 7.38–7.31 (m, 1H), 7.21–7.13 (m, 1H), 7.08 (d, *J* = 7.3 Hz, 1H), 6.73–6.65 (m, 1H), 4.14 (d, *J* = 5.0 Hz, 1H), 3.17 (s, 1H), 2.92 (s, 3H), 1.51 (s, 3H), 1.44 (s, 3H), 0.94 (s, 9H); ^{13}C NMR (151 MHz, $CDCl_3$) δ 177.2, 147.1,

146.2, 144.5, 134.8, 130.2, 127.7, 127.1, 126.4, 124.8, 124.5, 121.0, 117.2, 111.7, 74.6, 51.9, 47.4, 36.7, 28.6, 28.4, 26.6; IR (neat, cm^{-1}): 3364, 2956, 1723, 1505, 1478, 1353, 1250, 1145, 1064, 1105, 990, 753; ESI HRMS *m/z* $(M+Na)^+$ calcd 403.1992, obsd 403.1995.

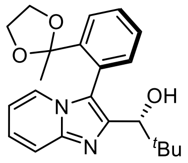

(±)-2,2-Dimethyl-1-(3-(2-(2-methyl-1,3-dioxolan-2-yl)phenyl)imidazo[1,2-*a*]pyridin-2-yl) propan-1-ol (3u). Yield = 72%; White solid; ^1H NMR (400 MHz, $CDCl_3$) δ 7.83 (dd, *J* = 7.9, 1.4 Hz, 1H), 7.61 (d, *J* = 9.1 Hz, 1H), 7.55–7.47 (m, 1H), 7.46–7.38 (m, 1H), 7.37 (d, *J* = 6.9 Hz, 1H), 7.20 (dd, *J* = 7.5, 1.2 Hz, 1H), 7.17–7.10 (m, 1H), 6.69–6.61 (m, 1H), 4.13 (d, *J* = 6.6 Hz, 1H), 3.88 (q, *J* = 6.9 Hz, 1H), 3.78 (q, *J* = 6.9 Hz, 1H), 3.67 (q, *J* = 6.9 Hz, 1H), 3.59 (q, *J* = 6.9 Hz, 1H), 2.95 (d, *J* = 6.8 Hz, 1H), 1.36 (s, 3H), 0.99 (s, 9H); ^{13}C NMR (126 MHz, $CDCl_3$) δ 145.1, 144.9, 144.1, 134.9, 129.8, 128.5, 127.1, 126.6, 124.4, 123.9, 121.7, 117.4, 111.5, 109.1, 75.1, 65.3, 64.4, 36.4, 26.7, 26.2; IR (neat, cm^{-1}): 3453, 2951, 1681, 1653, 1505, 1478, 1361, 1263, 1043, 949, 904, 854, 801, 753; ESI HRMS *m/z* $(M+Na)^+$ calcd 389.1836, obsd 389.1843.

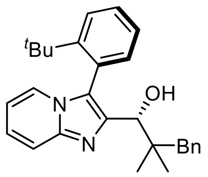

(±)-1-(3-(2-(*tert*-Butyl)phenyl)imidazo[1,2-*a*]pyridin-2-yl)-2,2-dimethyl-3-phenylpropan-1-ol (3v). Yield = 70%; White solid; ^1H NMR (400 MHz, $CDCl_3$) δ 7.67 (dd, *J* = 8.2, 1.3 Hz, 2H), 7.49–7.37 (m, 2H), 7.28–7.21 (m, 1H), 7.21–7.12 (m, 4H), 7.12–7.07 (m, 2H), 7.03 (dd, *J* = 7.6, 1.6 Hz, 1H), 6.76–6.68 (m, 1H), 4.30 (d, *J* = 7.4 Hz, 1H), 3.08 (s, 1H), 2.72 (s, 2H), 1.11 (s, 9H), 1.02 (s, 3H), 0.76 (s, 3H); ^{13}C NMR (101 MHz, $CDCl_3$) δ 152.3, 144.5, 143.9, 139.3, 135.4, 131.0, 129.9, 128.4, 127.6, 126.1, 126.0, 125.7, 124.3, 124.2, 123.6, 117.4, 112.2, 74.5, 45.0, 40.8, 36.7, 31.6, 23.5, 22.7; IR (neat, cm^{-1}): 337, 2960, 1504, 1494, 1363, 1343, 1275, 1055, 1015, 755, 739, 702; ESI HRMS *m/z* $(M+Na)^+$ calcd 435.2407, obsd 435.2408.

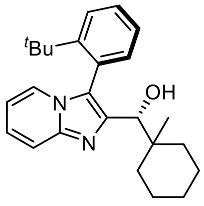

(±)-(3-(2-(*tert*-Butyl)phenyl)imidazo[1,2-*a*]pyridin-2-yl)(1-methylcyclohexyl)methanol (3w). Yield = 64%; White solid; ^1H NMR (600 MHz, $CDCl_3$) δ 7.68 (d, *J* = 8.2 Hz, 1H), 7.62

(d, J = 9.1 Hz, 1H), 7.49–7.42 (m, 1H), 7.38 (d, J = 6.8 Hz, 1H), 7.31–7.25 (m, 1H), 7.19–7.11 (m, 1H), 7.07 (d, J = 7.5 Hz, 1H), 6.72–6.66 (m, 1H), 4.21 (d, J = 8.9 Hz, 1H), 2.90 (d, J = 19.8 Hz, 1H), 1.62–1.54 (m, 1H), 1.53–1.44 (m, 2H), 1.44–1.33 (m, 3H), 1.34–1.23 (m, 2H), 1.16–1.05 (m, 11H), 1.01 (s, 3H); ^{13}C NMR (151 MHz, $CDCl_3$) δ 152.3, 144.5, 143.7, 135.5, 129.9, 128.4, 126.2, 126.1, 124.2, 124.0, 123.5, 117.3, 112.0, 75.7, 39.6, 36.6, 34.3, 34.0, 31.6, 26.5, 21.9, 21.8, 19.4; IR (neat, cm^{-1}): 3356, 2293, 1504, 1481, 1465, 1364, 1344, 1274, 1053, 1013, 738; ESI HRMS *m/z* $(M+Na)^+$ calcd 427.2356, obsd 427.2369.

5. General Procedure for the Synthesis of Substrates

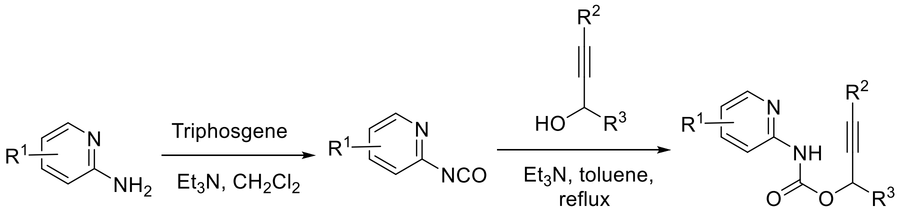

All the substrates were prepared according to the method reported previously [1]. To a solution of triphosgene (2.0 mmol, 1.0 equiv) in anhydrous CH_2Cl_2 (8 mL) was added dropwise the aminoarene (4.0 mmol, 2.0 equiv) at 0 °C followed by Et_3N (4.0 mmol, 2.0 equiv). The reaction mixture was stirred at r.t. for 6 h. The solvent was then removed under reduced pressure. The residue was dissolved in toluene (6.0 mL). The propargyl alcohol (2.0 mmol, 1.0 equiv) and Et_3N (4.0 mmol, 2.0 equiv) were added. The resulting mixture was heated to reflux and maintained at this temperature for 36 h. The solvent was evaporated under reduced pressure and the residue was chromatographed through silica gel eluting with ethyl acetate/hexanes to give the product.

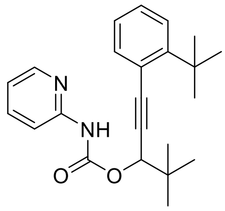

1-(2-(*tert*-Butyl)phenyl)-4,4-dimethylpent-1-yn-3-yl pyridin-2-ylcarbamate (2a). White solid; ^{1}H NMR (500 MHz, $CDCl_3$) δ 9.39 (s, 1H), 8.42 (d, J = 5.0 Hz, 1H), 8.07 (d, J = 8.5 Hz, 1H), 7.75–7.68 (m, 1H), 7.50 (dd, J = 7.6, 1.6 Hz, 1H), 7.36 (dd, J = 8.0, 1.2 Hz, 1H), 7.27–7.23 (m, 1H), 7.17–7.09 (m, 1H), 6.99 (ddd, J = 7.3, 5.0, 1.1 Hz, 1H), 5.53 (s, 1H), 1.49 (s, 9H), 1.15 (s, 9H); ^{13}C NMR (126 MHz, $CDCl_3$) δ 153.1, 152.3, 151.7, 148.0, 138.7, 135.9, 128.7, 125.6, 121.0, 118.9, 112.8, 91.6, 87.8, 74.1, 36.1, 35.9, 30.2, 26.0; IR (neat, cm^{-1}): 2964, 1734, 1587, 1537, 1439, 1307, 1217, 1059, 778, 757; ESI HRMS *m/z* $(M+Na)^+$ calcd 387.2043, obsd 387.2056.

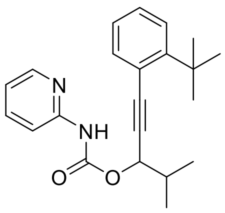

1-(2-(*tert*-Butyl)phenyl)-4-methylpent-1-yn-3-yl pyridin-2-ylcarbamate (2b). White solid; ^{1}H NMR (500 MHz, $CDCl_3$) δ 10.36 (s, 1H), 8.51 (d, *J* = 5.0 Hz, 1H), 8.11 (d, *J* = 8.5 Hz, 1H), 7.76–7.67 (m, 1H), 7.56–7.48 (m, 1H), 7.37 (d, *J* = 8.0 Hz, 1H), 7.30–7.22 (m, 1H), 7.18–7.11 (m, 1H), 6.96 (dd, *J* = 7.3, 5.0 Hz, 1H), 5.66 (d, *J* = 5.6 Hz, 1H), 2.32–2.19 (m, 1H), 1.51 (s, 9H), 1.16 (dd, *J* = 16.3, 6.8 Hz, 6H); ^{13}C NMR (126 MHz, $CDCl_3$) δ 153.1, 152.6, 151.8, 147.9, 138.7, 135.9, 128.7, 125.6, 120.9, 118.7, 112.9, 91.3, 87.9, 71.1, 35.9, 33.1, 30.2, 18.6, 18.0; ESI HRMS *m/z* $(M+Na)^+$ calcd 373.1886, obsd 373.1891.

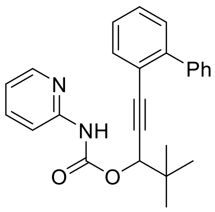

1-([1,1'-Biphenyl]-2-yl)-4,4-dimethylpent-1-yn-3-yl pyridin-2-ylcarbamate (2c). White solid; ^{1}H NMR (500 MHz, $CDCl_3$) δ 9.28 (s, 1H), 8.43–8.29 (m, 1H), 8.09–8.00 (m, 1H), 7.75–7.67 (m, 1H), 7.58 (d, *J* = 7.7 Hz, 1H), 7.53 (d, *J* = 7.2 Hz, 2H), 7.41–7.32 (m, 4H), 7.33–7.22 (m, 2H), 7.00–6.93 (m, 1H), 5.38–5.28 (m, 1H), 0.94 (s, 9H); ^{13}C NMR (126 MHz, $CDCl_3$) δ 153.0, 152.2, 148.0, 144.4, 140.6, 138.6, 133.5, 129.6, 129.4, 128.8, 128.1, 127.5, 127.1, 121.2, 118.8, 112.8, 88.6, 85.9, 73.8, 35.8, 25.8;

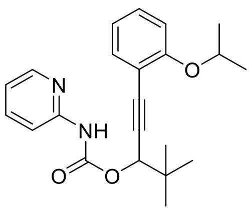

1-(2-(*tert*-Butyl)phenyl)-4,4-dimethylpent-1-yn-3-yl pyridin-2-ylcarbamate (2d). White solid; ^{1}H NMR (500 MHz, $CDCl_3$) δ 9.91 (s, 1H), 8.51–8.44 (m, 1H), 8.10 (d, *J* = 8.4 Hz, 1H), 7.74–7.66 (m, 1H), 7.41 (dd, *J* = 7.5, 1.5 Hz, 1H), 7.28–7.21 (m, 1H), 6.96 (dd, *J* = 7.3, 5.1 Hz, 1H), 6.90–6.83 (m, 2H), 5.53 (s, 1H), 4.59 (hept, *J* = 6.0 Hz, 1H), 1.34 (t, *J* = 5.7 Hz, 6H), 1.16 (s, 9H); ^{13}C NMR (126 MHz, $CDCl_3$) δ 159.2, 153.2, 152.5, 148.0, 138.6, 133.8, 129.8, 120.4, 118.7, 114.2, 113.5, 112.8, 89.5, 83.1, 73.9, 71.2, 36.1, 25.9, 22.3, 22.3.

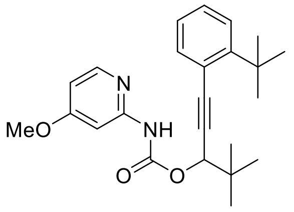

1-(2-(*tert*-Butyl)phenyl)-4,4-dimethylpent-1-yn-3-yl (4-methoxypyridin-2-yl)carbamate (2e). White solid; ^{1}H NMR (500 MHz, $CDCl_3$) δ 10.33 (s, 1H), 8.28 (d, *J* = 6.0 Hz, 1H), 7.71 (s, 1H), 7.50 (dd, *J* = 7.6, 1.6 Hz, 1H), 7.39–7.32 (m, 1H), 7.27–7.23 (m, 1H), 7.17–7.09 (m, 1H), 6.53 (dd, *J* = 5.9, 2.4 Hz, 1H), 5.56 (s, 1H), 3.89 (s, 3H), 1.50 (s, 9H), 1.15 (s, 9H); ^{13}C NMR (126 MHz, $CDCl_3$) δ 167.8, 154.4, 153.4, 151.7, 148.7, 135.9, 128.7, 125.6, 121.0, 106.9, 97.3, 91.6, 87.8, 74.0, 55.4, 36.1, 35.9, 30.2, 26.1; IR (neat, cm^{-1}): 2965, 1731, 1601, 1585, 1538, 1481, 1223, 1046, 996, 954, 758; ESI HRMS *m/z* $(M+Na)^+$ calcd 391.2356, obsd 391.2352.

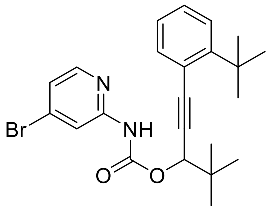

1-(2-(*tert*-Butyl)phenyl)-4,4-dimethylpent-1-yn-3-yl (4-bromopyridin-2-yl)carbamate (2f). White solid; ^{1}H NMR (600 MHz, $CDCl_3$) δ 10.14 (s, 1H), 8.39 (d, *J* = 5.5 Hz, 1H), 8.21 (s, 1H), 7.51 (d, *J* = 7.6 Hz, 1H), 7.39 (d, *J* = 8.0 Hz, 1H), 7.32–7.24 (m, 1H), 7.20–7.12 (m, 1H), 7.00 (d, *J* = 4.3 Hz, 1H), 5.60 (s, 1H), 1.52 (s, 9H), 1.17 (s, 9H); ^{13}C NMR (151 MHz, $CDCl_3$) δ 153.5, 153.0, 151.7, 148.6, 146.4, 135.9, 128.8, 125.7, 120.8, 119.3, 113.2, 91.2, 88.1, 74.4, 36.2, 35.9, 30.2, 26.0; IR (neat, cm^{-1}): 2965, 1738, 1578, 1530, 1414, 1252, 1212, 1108, 1048, 921, 757, 719; ESI HRMS *m/z* $(M+Na)^+$ calcd 465.1148, obsd 465.1174.

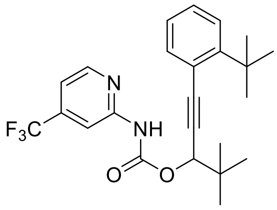

1-(2-(*tert*-Butyl)phenyl)-4,4-dimethylpent-1-yn-3-yl(4-(trifluoromethyl)pyridin-2-yl)-carbamate (2g). White solid; ^{1}H NMR (500 MHz, $CDCl_3$) δ 9.21 (s, 1H), 8.54 (d, *J* = 5.2 Hz, 1H), 8.34 (s, 1H), 7.46 (d, *J* = 7.6 Hz, 1H), 7.34 (d, *J* = 8.0 Hz, 1H), 7.28–7.20 (m, 1H), 7.17 (d, *J* = 5.1 Hz, 1H), 7.15–7.07 (m, 1H), 5.52 (s, 1H), 1.46 (s, 9H), 1.13 (s, 9H); ^{13}C NMR (151 MHz, $CDCl_3$) δ 153.0, 152.7, 151.6, 148.9, 140.8 (q, *J* = 34.0 Hz),135.8, 128.7, 125.53 (d, *J* = 2.1 Hz), 122.7 (q, *J* = 273.5 Hz), 120.5, 114.4 (d, *J* = 3.9 Hz),108.9, 90.9, 88.0, 74.6, 36.0, 35.8, 30.0, 25.8; ^{19}F NMR (471 MHz, $CDCl_3$) δ -65.0; IR (neat, cm^{-1}): 2966, 1736, 1543, 1480, 1405, 1272, 1221, 1049, 950, 757; ESI HRMS *m/z* $(M+Na)^+$ calcd 455.1917, obsd 455.1924.

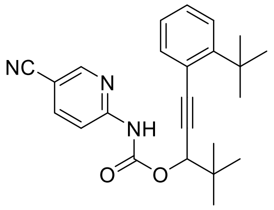

1-(2-(*tert*-Butyl)phenyl)-4,4-dimethylpent-1-yn-3-yl (5-cyanopyridin-2-yl)carbamate (2h). White solid; ^{1}H NMR (500 MHz, $CDCl_3$) δ 8.78 (s, 1H), 8.64 (d, *J* = 2.2 Hz, 1H), 8.20 (d, *J* = 8.8 Hz, 1H), 7.94 (dd, *J* = 8.8, 2.2 Hz, 1H), 7.49 (d, *J* = 7.6 Hz, 1H), 7.35 (d, *J* = 8.0 Hz, 1H), 7.28–7.23 (m, 1H), 7.17–7.09 (m, 1H), 5.48 (s, 1H), 1.47 (s, 9H), 1.15 (s, 9H); ^{13}C NMR (126 MHz, $CDCl_3$) δ 154.6, 152.3, 151.9, 151.8, 141.8, 136.0, 128.9, 125.7, 120.6, 116.9, 112.5, 104.5, 90.7, 88.5, 75.3, 36.0, 35.9, 30.2, 26.0; IR (neat, cm^{-1}): 2967, 2230, 1742, 1600, 1530, 1481, 1387, 1225, 1200, 1051, 956, 844, 753; ESI HRMS *m/z* $(M+Na)^+$ calcd 412.1995, obsd 412.2003.

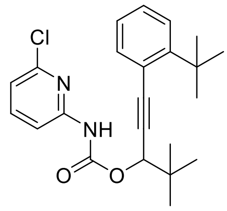

1-(2-(*tert*-Butyl)phenyl)-4,4-dimethylpent-1-yn-3-yl (6-chloropyridin-2-yl)carbamate (2i). White solid; ^{1}H NMR (600 MHz, $CDCl_3$) δ 7.95 (d, *J* = 8.2 Hz, 1H), 7.71–7.63 (m, 1H), 7.62–7.54 (m, 1H), 7.50 (dd, *J* = 7.6, 1.6 Hz, 1H), 7.37 (dd, *J* = 8.0, 1.3 Hz, 1H), 7.31–7.23 (m, 1H), 7.19–7.11 (m, 1H), 7.04 (d, *J* = 7.7 Hz, 1H), 5.42 (s, 1H), 1.50 (s, 9H), 1.14 (s, 9H); ^{13}C NMR (151 MHz, $CDCl_3$) δ 152.3, 151.8, 151.5, 149.2, 141.0, 135.8, 128.7, 125.6, 125.6, 120.8, 119.0, 110.5, 91.1, 88.1, 74.7, 36.0, 35.9, 30.1, 25.9; IR (neat, cm^{-1}): 2964, 1741, 1574, 1520, 1440, 1394, 1208, 1163, 1063, 960, 915, 790, 757; ESI HRMS *m/z* $(M+Na)^+$ calcd 421.1653, obsd 421.1635.

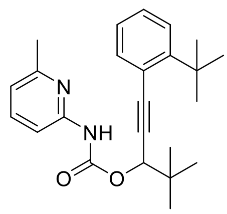

1-(2-(*tert*-Butyl)phenyl)-4,4-dimethylpent-1-yn-3-yl (6-methylpyridin-2-yl)carbamate (2j). White solid; ^{1}H NMR (500 MHz, $CDCl_3$) δ 8.05 (s, 1H), 7.82 (d, *J* = 8.3 Hz, 1H), 7.63 – 7.56 (m, 1H), 7.49 (dd, *J* = 7.6, 1.6 Hz, 1H), 7.38–7.33 (m, 1H), 7.29–7.21 (m, 1H), 7.17–7.09 (m, 1H), 6.86 (d, *J* = 7.5 Hz, 1H), 5.42 (s, 1H), 2.47 (s, 3H), 1.49 (s, 9H), 1.10 (s, 9H); ^{13}C NMR (126 MHz, $CDCl_3$) δ 157.0, 152.8, 151.7, 151.1, 138.8, 135.8, 128.6, 125.6, 121.0,

118.5, 109.5, 91.5, 87.8, 74.1, 36.0, 35.9, 30.1, 25.9, 24.1; IR (neat, cm^{-1}): 2964, 1736, 1582, 1537, 1460, 1395, 1262, 1211, 1076, 985, 757; ESI HRMS *m/z* $(M+Na)^+$ calcd 401.2199, obsd 401.2209.

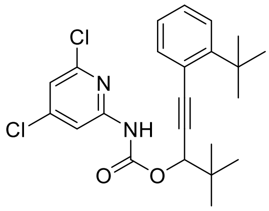

1-(2-(*tert*-Butyl)phenyl)-4,4-dimethylpent-1-yn-3-yl (4,6-dichloropyridin-2-yl)carbamate (2k). Pale yellow oil; ^{1}H NMR (500 MHz, $CDCl_3$) δ 8.02 (d, *J* = 1.5 Hz, 1H), 7.56 (s, 1H), 7.49 (dd, *J* = 7.6, 1.6 Hz, 1H), 7.37 (dd, *J* = 8.1, 1.3 Hz, 1H), 7.29–7.24 (m, 1H), 7.18–7.10 (m, 1H), 7.06 (d, *J* = 1.5 Hz, 1H), 5.41 (s, 1H), 1.49 (s, 9H), 1.13 (s, 9H); ^{13}C NMR (126 MHz, $CDCl_3$) δ 152.1, 151.9, 151.8, 149.9, 147.7, 135.9, 128.8, 125.7, 125.7, 120.7, 119.1, 111.0, 90.8, 88.3, 75.0, 36.0, 35.9, 30.2, 25.9; IR (neat, cm^{-1}): 2964, 1474, 1562, 1515, 1417, 1386, 1207, 1161, 1066, 932, 825, 796, 757; ESI HRMS *m/z* $(M+Na)^+$ calcd 455.1264, obsd 455.1269.

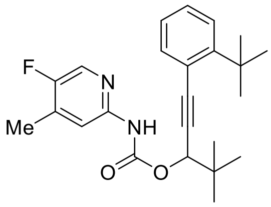

1-(2-(*tert*-Butyl)phenyl)-4,4-dimethylpent-1-yn-3-yl(5-fluoro-4-methylpyridin-2-yl)carbamate (2l). White solid; ^{1}H NMR (850 MHz, $CDCl_3$) δ 9.34 (d, *J* = 14.4 Hz, 1H), 8.18 (s, 1H), 7.96 (s, 1H), 7.50 (dd, *J* = 7.7, 1.5 Hz, 1H), 7.36 (d, *J* = 8.0 Hz, 1H), 7.30–7.22 (m, 1H), 7.18–7.11 (m, 1H), 5.53 (s, 1H), 2.34 (s, 3H), 1.49 (s, 9H), 1.15 (s, 9H); ^{13}C NMR (214 MHz, $CDCl_3$) δ155.6 (d, *J* = 247.6 Hz), 153.1, 151.7, 148.2, 137.1 (d, *J* = 15.7 Hz), 135.9, 134.5 (d, *J* = 27.3 Hz), 128.7, 125.6 (d, *J* = 3.1 Hz), 120.9, 114.8, 91.4, 87.9, 74.2, 36.1, 35.9, 30.2, 26.0, 15.0 (d, *J* = 2.9 Hz); IR (neat, cm^{-1}): 2966, 1736, 1543, 1480, 1405, 1272, 1221, 1151, 1049, 952, 757; ESI HRMS *m/z* $(M+H)^+$ calcd 397.2286, obsd 397.2292.

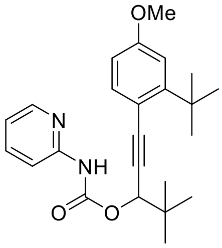

1-(2-(*tert*-Butyl)-4-methoxyphenyl)-4,4-dimethylpent-1-yn-3-yl pyridin-2-ylcarbamate (2m). White solid; ^{1}H NMR (400 MHz, $CDCl_3$) δ 9.82 (s, 1H), 8.51–8.43 (m, 1H), 8.10 (d, *J* = 8.5 Hz, 1H), 7.76–7.69 (m, 1H), 7.46 (d, *J* = 8.5 Hz, 1H), 7.02–6.96 (m, 1H), 6.93 (d, *J* = 2.6 Hz, 1H), 6.68 (dd, *J* = 8.5, 2.6 Hz, 1H), 5.56 (s, 1H), 3.82 (s, 3H), 1.50 (s, 9H), 1.15 (s,

9H); ^{13}C NMR (101 MHz, $CDCl_3$) δ 159.7, 153.7, 153.2, 152.5, 148.0, 138.7, 137.3, 118.7, 113.2, 112.9, 112.9, 109.8, 89.9, 87.8, 74.2, 55.4, 36.1, 36.0, 30.0, 26.1; IR (neat, cm^{-1}): 2960, 1735, 1589, 1537, 1439, 1307, 1217, 1050; ESI HRMS *m/z* $(M+Na)^+$ calcd 417.2149, obsd 417.2158.

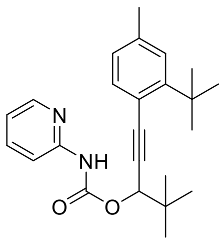

1-(2-(*tert*-Butyl)-4-methylphenyl)-4,4-dimethylpent-1-yn-3-yl pyridin-2-ylcarbamate (2n). White solid; ^1H NMR (500 MHz, $CDCl_3$) δ 9.44 (s, 1H), 8.43 (d, *J* = 4.9 Hz, 1H), 8.07 (d, *J* = 8.4 Hz, 1H), 7.74–7.67 (m, 1H), 7.39 (d, *J* = 7.7 Hz, 1H), 7.16 (s, 1H), 7.01–6.92 (m, 2H), 5.53 (s, 1H), 2.34 (s, 3H), 1.48 (s, 9H), 1.14 (s, 9H); ^{13}C NMR (126 MHz, $CDCl_3$) δ 153.1, 152.3, 151.5, 148.0, 138.7, 138.6, 135.9, 126.6, 126.3, 118.8, 117.9, 112.8, 90.8, 88.0, 74.2, 36.1, 35.8, 30.2, 26.0, 21.9; IR (neat, cm^{-1}): 2963, 1735, 1587, 1537, 1439, 1307, 1216, 1059, 1045, 985, 962, 777; ESI HRMS *m/z* $(M+Na)^+$ calcd 401.2199, obsd 401.2204.

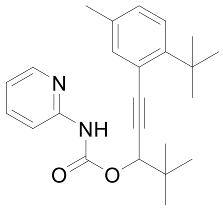

1-(2-(*tert*-Butyl)-5-methylphenyl)-4,4-dimethylpent-1-yn-3-yl pyridin-2-ylcarbamate (2o). White solid; ^1H NMR (400 MHz, $CDCl_3$) δ 9.72 (s, 1H), 8.52–8.42 (m, 1H), 8.10 (d, *J* = 8.4 Hz, 1H), 7.77–7.70 (m, 1H), 7.34 (d, *J* = 2.0 Hz, 1H), 7.26 (d, *J* = 8.1 Hz, 1H), 7.08 (dd, *J* = 8.2, 2.1 Hz, 1H), 7.02–6.98 (m, 1H), 5.56 (s, 1H), 2.29 (s, 3H), 1.49 (s, 9H), 1.17 (s, 9H); ^{13}C NMR (101 MHz, $CDCl_3$) δ 153.2, 152.4, 148.8, 148.0, 138.7, 136.4, 135.1, 129.5, 125.6, 120.7, 118.8, 112.9, 91.1, 88.0, 74.1, 36.1, 35.6, 30.3, 26.1, 20.5; IR (neat, cm^{-1}): 2963, 1734, 1587, 1537, 1439, 1307, 1255, 1217, 1059, 1047, 777; ESI HRMS *m/z* $(M+Na)^+$ calcd 401.2199, obsd 401.2212.

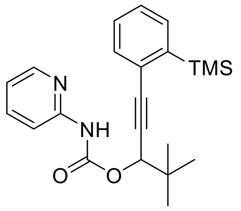

4,4-Dimethyl-1-(2-(trimethylsilyl)phenyl)pent-1-yn-3-yl pyridin-2-ylcarbamate (2p). White solid; ^{1}H NMR (500 MHz, $CDCl_3$) δ 9.26 (s, 1H), 8.41 (dd, *J* = 5.1, 1.8 Hz, 1H), 8.06 (d, *J* = 8.5 Hz, 1H), 7.77–7.67 (m, 1H), 7.54–7.44 (m, 2H), 7.33 – 7.28 (m, 2H), 7.00 (dd, *J* = 7.3, 5.0 Hz, 1H), 5.50 (s, 1H), 1.14 (s, 9H), 0.34 (s, 9H); ^{13}C NMR (126 MHz, $CDCl_3$) δ 153.0, 152.2, 148.0, 142.6, 138.7, 134.0, 133.2, 128.8, 127.8, 127.8, 118.9, 112.9, 88.5, 87.9, 73.9, 35.9, 26.0, –0.9; IR (neat, cm^{-1}): 2959, 1734, 1588, 1537, 1439, 1307, 1255, 1217, 1059, 1035, 840, 778, 759; ESI HRMS *m/z* $(M+Na)^+$ calcd 403.1812, obsd 403.1827.

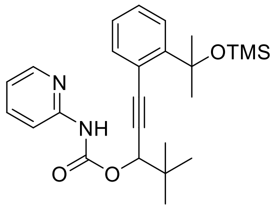

4,4-Dimethyl-1-(2-(2-((trimethylsilyl)oxy)propan-2-yl)phenyl)pent-1-yn-3-yl pyridin-2-ylcarbamate (2q). White solid; ^{1}H NMR (600 MHz, $CDCl_3$) δ 9.74 (s, 1H), 8.46 (s, 1H), 8.09 (d, *J* = 6.7 Hz, 1H), 7.75–7.68 (m, 2H), 7.48 (d, *J* = 7.6 Hz, 1H), 7.34–7.27 (m, 1H), 7.21–7.13 (m, 1H), 7.02–6.96 (m, 1H), 5.53 (s, 1H), 1.79 (s, 3H), 1.78 (s, 3H), 1.14 (s, 9H), 0.15 (s, 9H); ^{13}C NMR (151 MHz, $CDCl_3$) δ 153.1, 152.4, 151.3, 148.0, 138.7, 135.2, 128.7, 126.3, 125.5, 118.8, 118.7, 112.9, 91.0, 87.1, 76.2, 74.0, 36.1, 30.6, 30.6, 26.0, 2.7; IR (neat, cm^{-1}): 2957, 1735, 1588, 1537, 1439, 1308, 1252, 1217, 1177, 1040, 995, 840, 777, 759; ESI HRMS *m/z* $(M+Na)^+$ calcd 461.2231, obsd 461.2235.

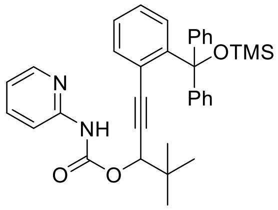

1-(2-(Diphenyl((trimethylsilyl)oxy)methyl)phenyl)-4,4-dimethylpent-1-yn-3-yl pyridin-2-ylcarbamate (2r). White solid; ^{1}H NMR (600 MHz, $CDCl_3$) δ 8.48 (s, 1H), 8.36–8.32 (m, 1H), 8.04 (d, *J* = 8.5 Hz, 1H), 7.88 (dd, *J* = 8.1, 1.3 Hz, 1H), 7.75–7.69 (m, 1H), 7.56–7.49 (m, 4H), 7.41 (dd, *J* = 7.6, 1.5 Hz, 1H), 7.36–7.28 (m, 1H), 7.25–7.15 (m, 5H), 7.16–7.09 (m, 2H), 7.01–6.96 (m, 1H), 5.08 (s, 1H), 0.89 (s, 9H), -0.20 (s, 9H); ^{13}C NMR (151 MHz, $CDCl_3$) δ 152.5, 152.1, 148.8, 148.1, 145.4, 145.4, 138.6, 135.6, 128.7, 128.7, 128.3, 128.2, 127.7, 127.1, 127.0, 126.9, 121.8, 118.9, 112.6, 92.7, 87.0, 84.4, 73.4, 35.9, 25.9, 1.8; IR (neat, cm^{-1}): 2959, 1732, 1587, 1439, 1307, 1251, 1215, 1060, 885, 839, 758, 700; ESI HRMS *m/z* $(M+Na)^+$ calcd 585.2544, obsd 585.2543.

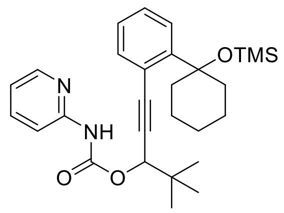

4,4-Dimethyl-1-(2-(1-((trimethylsilyl)oxy)cyclohexyl)phenyl)pent-1-yn-3-yl pyridin-2-ylcarbamate (2s). White solid; ^{1}H NMR (600 MHz, $CDCl_3$) δ 10.11–9.94 (m, 1H), 8.52–8.47 (m, 1H), 8.11 (d, J = 8.4 Hz, 1H), 7.74–7.68 (m, 1H), 7.50 (dd, J = 7.6, 1.5 Hz, 1H), 7.42 (d, J = 7.9 Hz, 1H), 7.30–7.22 (m, 1H), 7.21–7.13 (m, 1H), 7.00–6.95 (m, 1H), 5.56 (s, 1H), 2.40 (d, J = 12.0 Hz, 1H), 2.33 (d, J = 13.0 Hz, 1H), 1.93–1.73 (m, 4H), 1.67–1.60 (m, 1H), 1.58–1.50 (m, 2H), 1.30–1.21 (m, 1H), 1.15 (s, 9H), -0.08 (s, 9H); ^{13}C NMR (151 MHz, $CDCl_3$) δ 153.2, 152.6, 149.9, 147.9, 138.6, 136.0, 128.2, 126.6, 125.5, 121.4, 118.7, 112.9, 91.2, 87.4, 76.3, 74.1, 37.9, 37.7, 36.1, 26.1, 25.9, 22.5, 2.3; IR (neat, cm^{-1}): 2928, 1735, 1588, 1537, 1439, 1307, 1216, 1049, 1024, 838, 777, 755; ESI HRMS *m/z* (M+Na)$^+$ calcd 501.2544, obsd 501.2525.

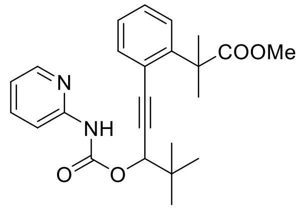

Methyl 2-(2-(4,4-dimethyl-3-((pyridin-2-ylcarbamoyl)oxy)pent-1-yn-1-yl)phenyl)-2-methylpropanoate (2t). White solid; ^{1}H NMR (600 MHz, $CDCl_3$) δ 9.46 (s, 1H), 8.49–8.45 (m, 1H), 8.08 (d, J = 8.4 Hz, 1H), 7.74–7.70 (m, 1H), 7.50 (dd, J = 7.7, 1.4 Hz, 1H), 7.39–7.32 (m, 2H), 7.27–7.19 (m, 1H), 7.03–6.99 (m, 1H), 5.51 (s, 1H), 3.65 (s, 3H), 1.65 (s, 3H), 1.63 (s, 3H), 1.13 (s, 9H); ^{13}C NMR (151 MHz, $CDCl_3$) δ 177.3, 153.0, 152.4, 148.1, 146.5, 138.6, 134.4, 128.8, 126.6, 125.1, 121.8, 118.8, 112.8, 91.3, 85.0, 73.6, 52.4, 47.2, 36.0, 26.7, 26.6, 26.0; IR (neat, cm^{-1}): 2970, 1736, 1587, 1537, 1439, 1307, 1254, 1217, 1145, 1059, 995, 778, 758; ESI HRMS *m/z* (M+Na)$^+$ calcd 431.1941, obsd 431.1923.

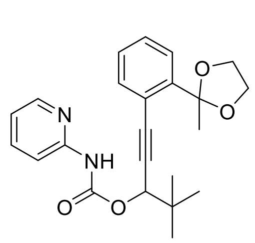

4,4-Dimethyl-1-(2-(2-methyl-1,3-dioxolan-2-yl)phenyl)pent-1-yn-3-ylpyridin-2-ylcarbamate (2u). White solid; ^{1}H NMR (500 MHz, $CDCl_3$) δ 9.34 (s, 1H), 8.47–8.41 (m, 1H), 8.08 (d,

J = 8.6 Hz, 1H), 7.73–7.68 (m, 1H), 7.57 (dd, J = 7.8, 1.4 Hz, 1H), 7.50 (dd, J = 7.6, 1.5 Hz, 1H), 7.29–7.26 (m, 1H), 7.26–7.20 (m, 1H), 7.00–6.96 (m, 1H), 5.52 (s, 1H), 4.00–3.97 (m, 2H), 3.77–3.71 (m, 2H), 1.79 (s, 3H), 1.15 (s, 9H); ^{13}C NMR (126 MHz, $CDCl_3$) δ 153.1, 152.3, 148.1, 144.7, 138.6, 134.9, 128.3, 127.8, 125.9, 120.2, 118.8, 112.8, 108.8, 90.3, 85.5, 74.0, 64.4, 64.4, 36.2, 26.1, 25.9; IR (neat, cm^{-1}): 2966, 1731, 1588, 1537, 1439, 1308, 1218, 1036, 995; ESI HRMS *m/z* (M+Na)$^+$ calcd 417.1785, obsd 417.1766.

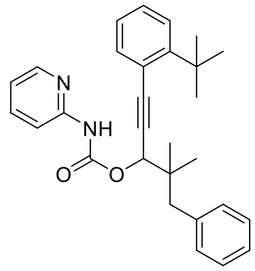

1-(2-(*tert*-Butyl)phenyl)-4,4-dimethyl-5-phenylpent-1-yn-3-yl pyridin-2-ylcarbamate (2v). White solid; ^{1}H NMR (400 MHz, $CDCl_3$) δ 9.67 (s, 1H), 8.50 (d, J = 5.0 Hz, 1H), 8.13 (d, J = 8.5 Hz, 1H), 7.80–7.70 (m, 1H), 7.57 (dd, J = 7.5, 1.6 Hz, 1H), 7.41 (dd, J = 8.0, 1.3 Hz, 1H), 7.38–7.20 (m, 6H), 7.22–7.14 (m, 1H), 7.07–6.98 (m, 1H), 5.62 (s, 1H), 2.99–2.79 (m, 2H), 1.56 (s, 9H), 1.16 (s, 3H), 1.14 (s, 3H); ^{13}C NMR (101 MHz, $CDCl_3$) δ 152.8, 152.2, 151.6, 147.8, 138.6, 137.7, 135.8, 130.7, 128.6, 128.0, 126.3, 125.5, 120.7, 118.7, 112.8, 91.3, 88.6, 73.3, 44.4, 39.7, 35.8, 30.1, 23.3, 23.0; IR (neat, cm^{-1}): 2964, 2922, 1735, 1586, 1536, 1438, 1307, 1217, 1058, 1034, 995, 778, 757, 702; ESI HRMS *m/z* (M+Na)$^+$ calcd 463.2356, obsd 463.2365.

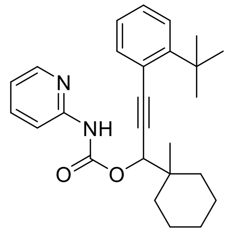

3-(2-(*tert*-Butyl)phenyl)-1-(1-methylcyclohexyl)prop-2-yn-1-yl pyridin-2-ylcarbamate (2w). White solid; ^{1}H NMR (400 MHz, $CDCl_3$) δ 9.36 (s, 1H), 8.48–8.38 (m, 1H), 8.07 (d, J = 8.4 Hz, 1H), 7.74–7.68 (m, 1H), 7.50 (dd, J = 7.6, 1.6 Hz, 1H), 7.36 (dd, J = 8.1, 1.3 Hz, 1H), 7.27–7.22 (m, 1H), 7.17–7.09 (m, 1H), 7.01–6.95 (m, 1H), 5.66 (s, 1H), 1.70–1.44 (m, 18H), 1.34–1.26 (m, 1H), 1.15 (s, 3H); ^{13}C NMR (151 MHz, $CDCl_3$) δ 153.2, 152.3, 151.7, 148.0, 138.7, 135.9, 128.6, 125.6, 121.0, 118.8, 112.8, 91.4, 88.3, 73.7, 38.6, 35.9, 34.3, 33.7, 30.2, 26.3, 21.8, 21.8, 20.4; IR (neat, cm^{-1}): 2929, 2862, 1734, 1587, 1537, 1438, 1307, 1254, 1216, 1058, 1034, 994, 777, 757; ESI HRMS *m/z* (M+Na)$^+$ calcd 427.2356, obsd 427.2369.

6. X-ray Crystallography of 3k

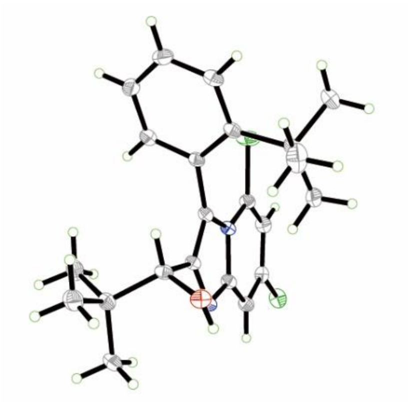

Table 1 Crystal data and structure refinement for 3k.

Identification code	3k
Empirical formula	$C_{22}H_{26}Cl_2N_2O$
Formula weight	405.35
Temperature/K	273.15
Crystal system	monoclinic
Space group	$P2_1/n$
a/Å	12.703(3)
b/Å	9.799(2)
c/Å	16.767(4)
α/°	90
β/°	97.536(4)
γ/°	90
Volume/Å^3	2069.0(9)
Z	4
ρ_{calc}g/cm^3	1.301
μ/mm^{-1}	0.328
F(000)	856.0
Crystal size/mm^3	0.5 × 0.3 × 0.3
Radiation	MoKα (λ = 0.71073)
2Θ range for data collection/°	3.792 to 57.078
Index ranges	$-16 \le h \le 17$, $-13 \le k \le 13$, $-21 \le l \le 22$
Reflections collected	15889
Independent reflections	4747 [R_{int} = 0.0310, R_{sigma} = 0.0295]
Data/restraints/parameters	4747/0/251
Goodness-of-fit on F^2	1.149
Final R indexes [I>=2σ (I)]	R_1 = 0.0518, wR_2 = 0.1341
Final R indexes [all data]	R_1 = 0.0551, wR_2 = 0.1385
Largest diff. peak/hole / e Å^{-3}	0.35/-0.24

7. References

[1] Hou, Z.-W.; Mao, Z.-Y.; Melcamu, Y. Y.; Lu, X.; Xu, H.-C. *Angew. Chem. Int. Ed.* **2018**, *57*, 1636-1639.

8. NMR Spectra for New Compounds

Compound **3a**

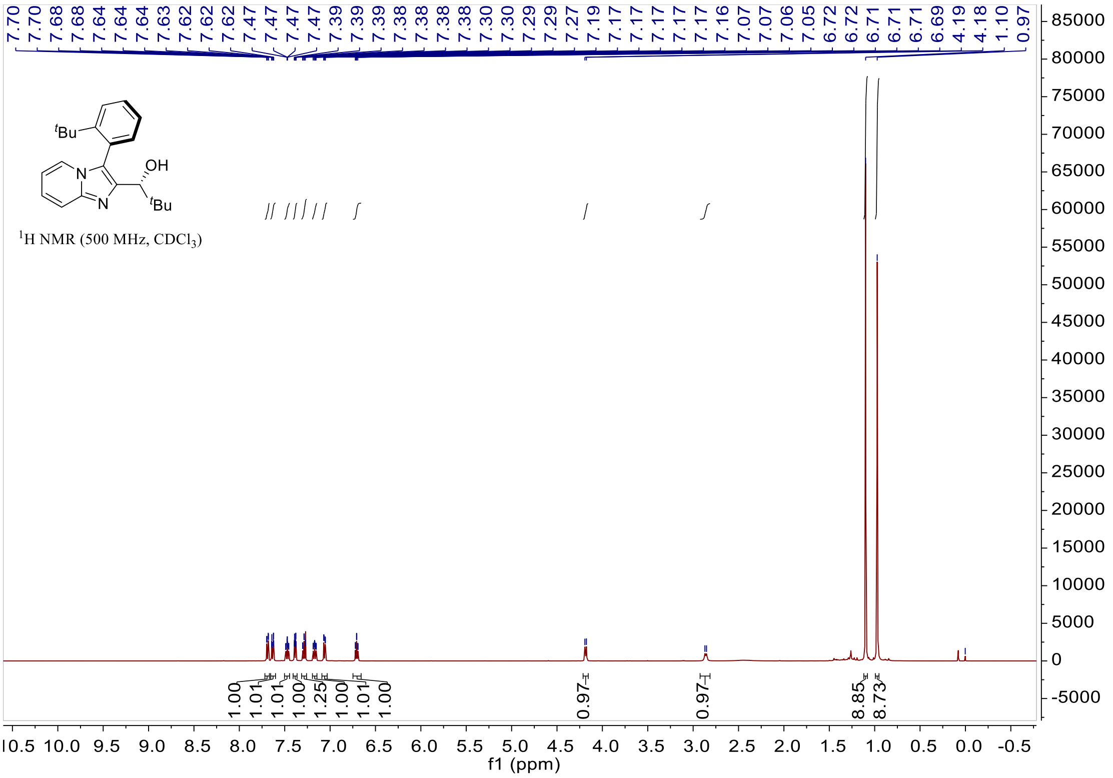

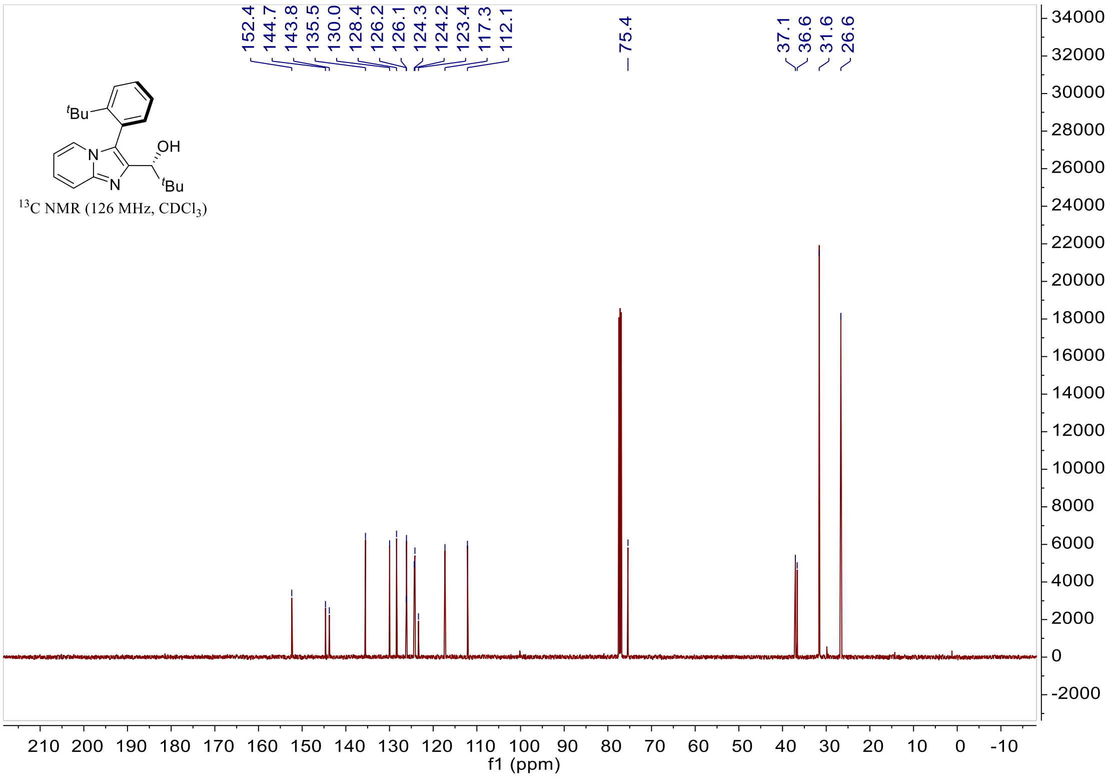

Compound **3e**

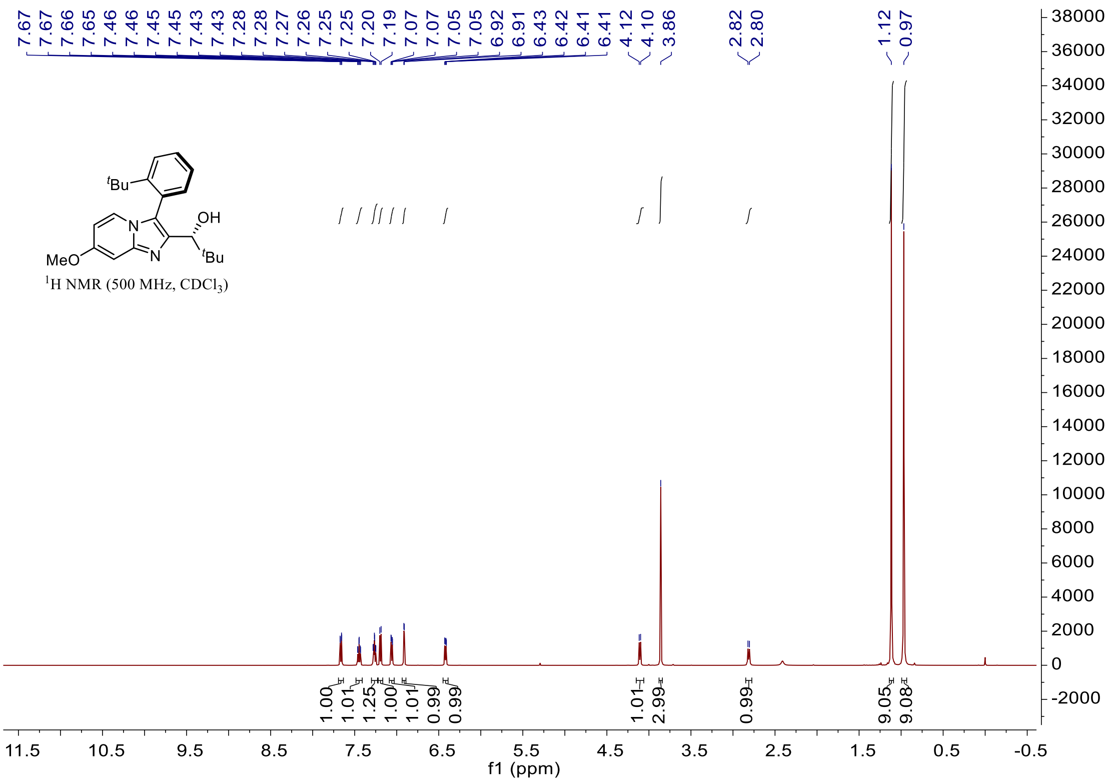

7.67 7.67 7.66 7.65 7.46 7.46 7.45 7.45 7.43 7.43 7.28 7.28 7.27 7.26 7.25 7.25 7.20 7.19 7.07 7.07 7.05 7.05 6.92 6.91 6.43 6.42 6.41 6.41 4.12 4.10 3.86 2.82 2.80 1.12 0.97
^tBu
OH
MeO
N
^tBu
1H NMR (500 MHz, CDCl3)
1.00 1.01 1.25 1.00 1.01 0.99 0.99 1.01 2.99 0.99 9.05 9.08
f1 (ppm)

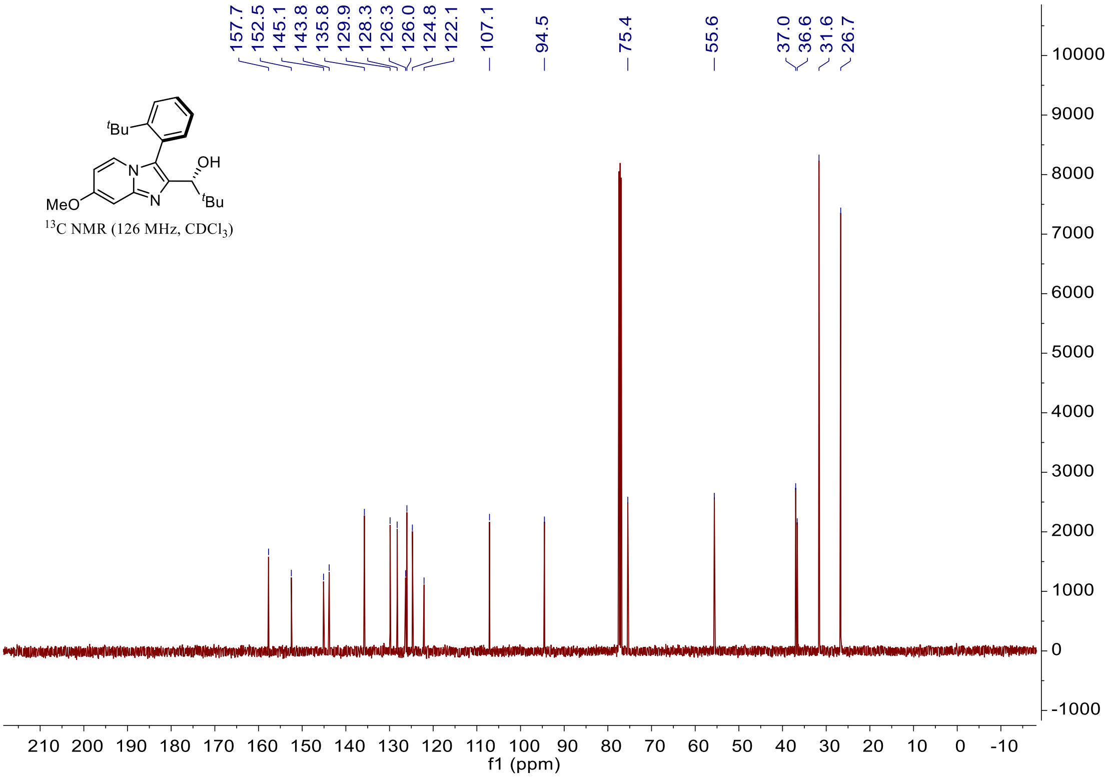

157.7 152.5 145.1 143.8 135.8 129.9 128.3 126.3 126.0 124.8 122.1 107.1 94.5 75.4 55.6 37.0 36.6 31.6 26.7
13C NMR (126 MHz, CDCl3)
f1 (ppm)

Compound **3f**

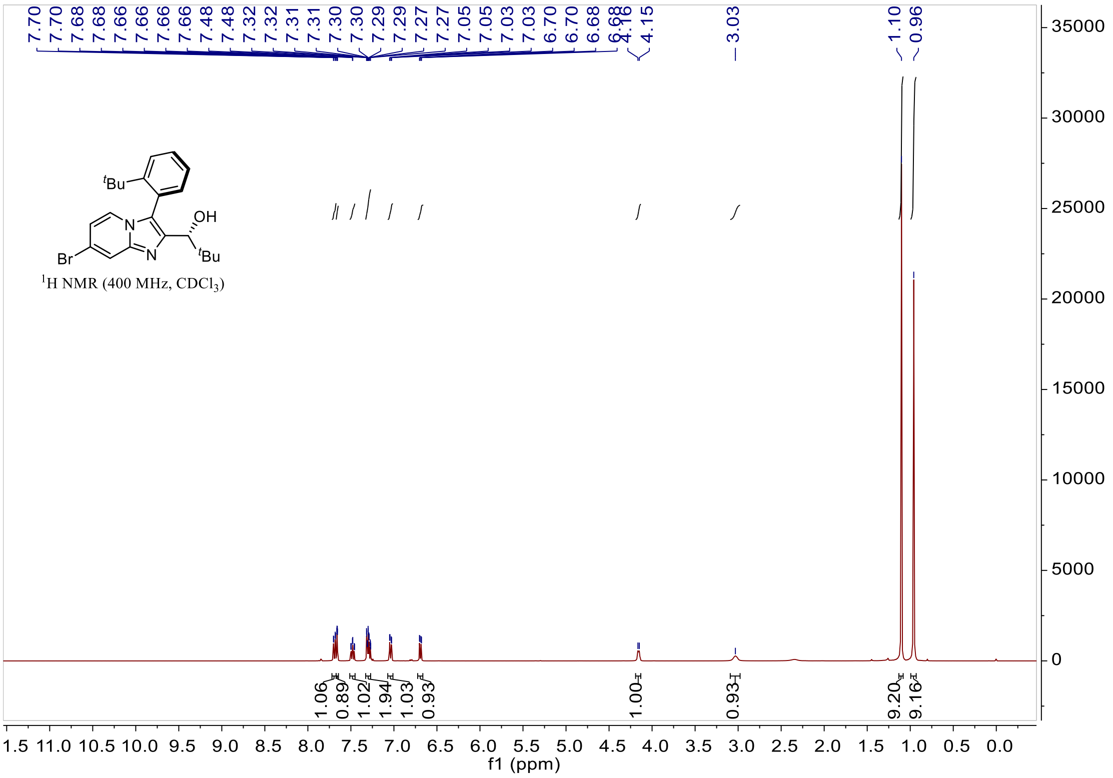
7.70
7.70
7.68
7.68
7.66
7.66
7.66
7.66
7.48
7.48
7.32
7.32
7.31
7.31
7.30
7.30
7.29
7.29
7.27
7.27
7.05
7.05
7.03
7.03
6.70
6.70
6.68
6.68
4.16
4.15
3.03
1.10
0.96
tBu
OH
Br
N
N
tBu
1H NMR (400 MHz, CDCl3)
1.06
0.89
1.02
1.94
1.03
0.93
1.00
0.93
9.20
9.16
f1 (ppm)

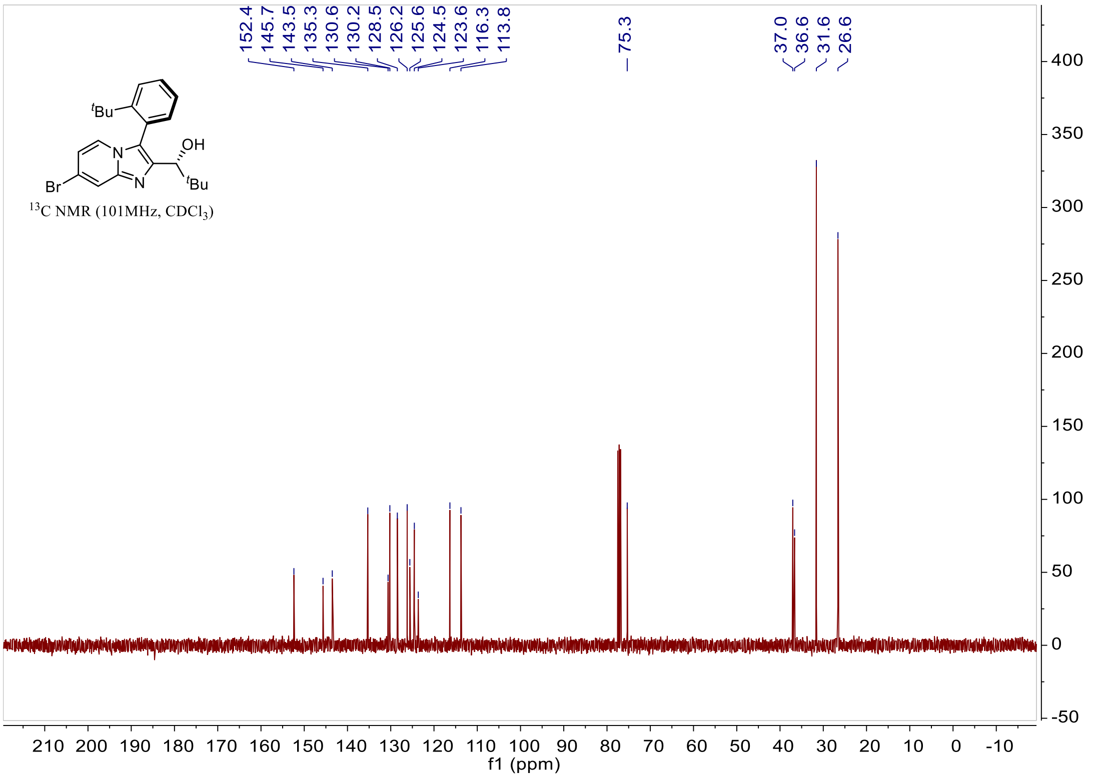
152.4
145.7
143.5
135.3
130.6
130.2
128.5
126.2
125.6
124.5
123.6
116.3
113.8
75.3
37.0
36.6
31.6
26.6
tBu
OH
Br
N
N
tBu
13C NMR (101MHz, CDCl3)
f1 (ppm)

Compound **3g**

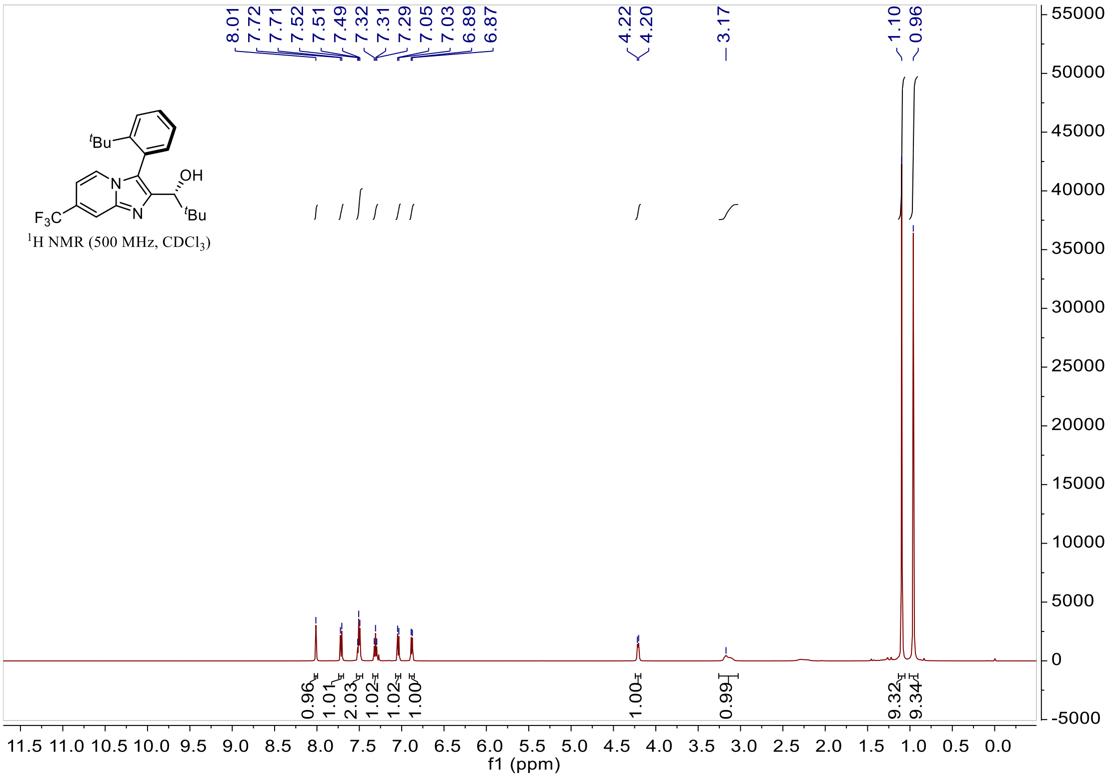

8.01
7.72
7.71
7.52
7.51
7.49
7.32
7.31
7.29
7.05
7.03
6.89
6.87
4.22
4.20
3.17
1.10
0.96
tBu
OH
F3C
N
N
tBu
1H NMR (500 MHz, CDCl3)
0.96
1.01
2.03
1.02
1.02
1.00
1.00
0.99
9.32
9.34
f1 (ppm)

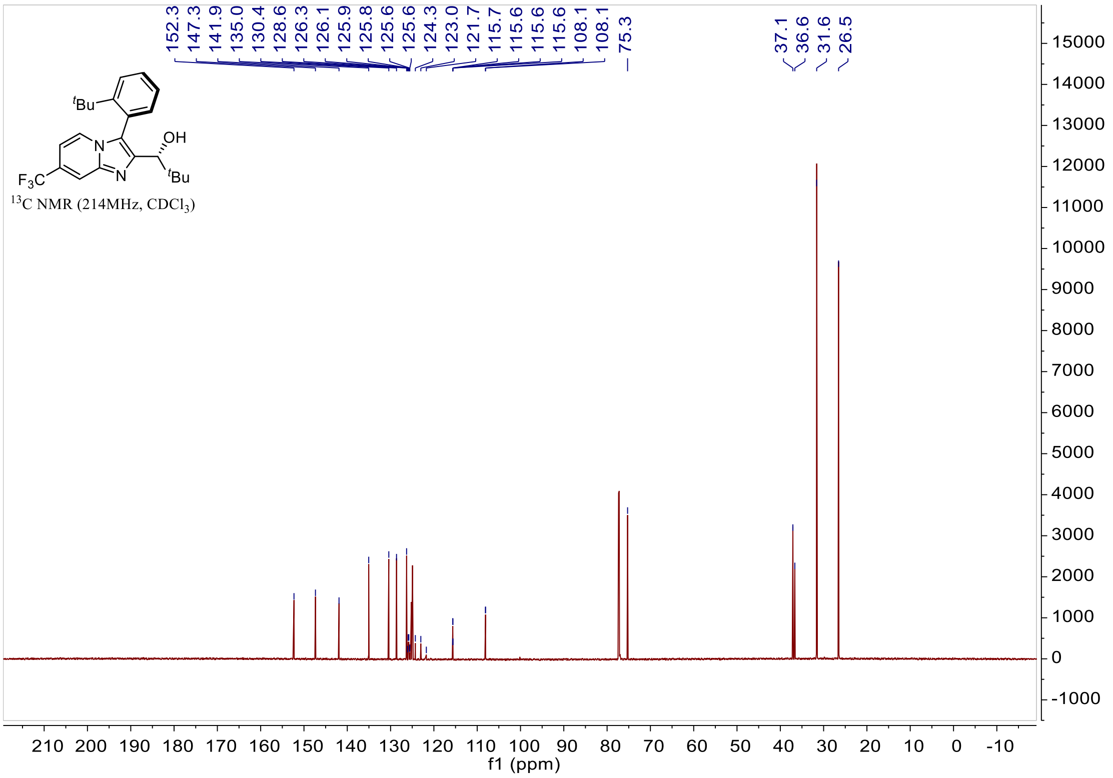

152.3
147.3
141.9
135.0
130.4
128.6
126.3
126.1
125.9
125.8
125.6
125.6
124.3
123.0
121.7
115.7
115.6
115.6
108.1
108.1
75.3
37.1
36.6
31.6
26.5
tBu
OH
F3C
N
N
tBu
13C NMR (214MHz, CDCl3)
f1 (ppm)

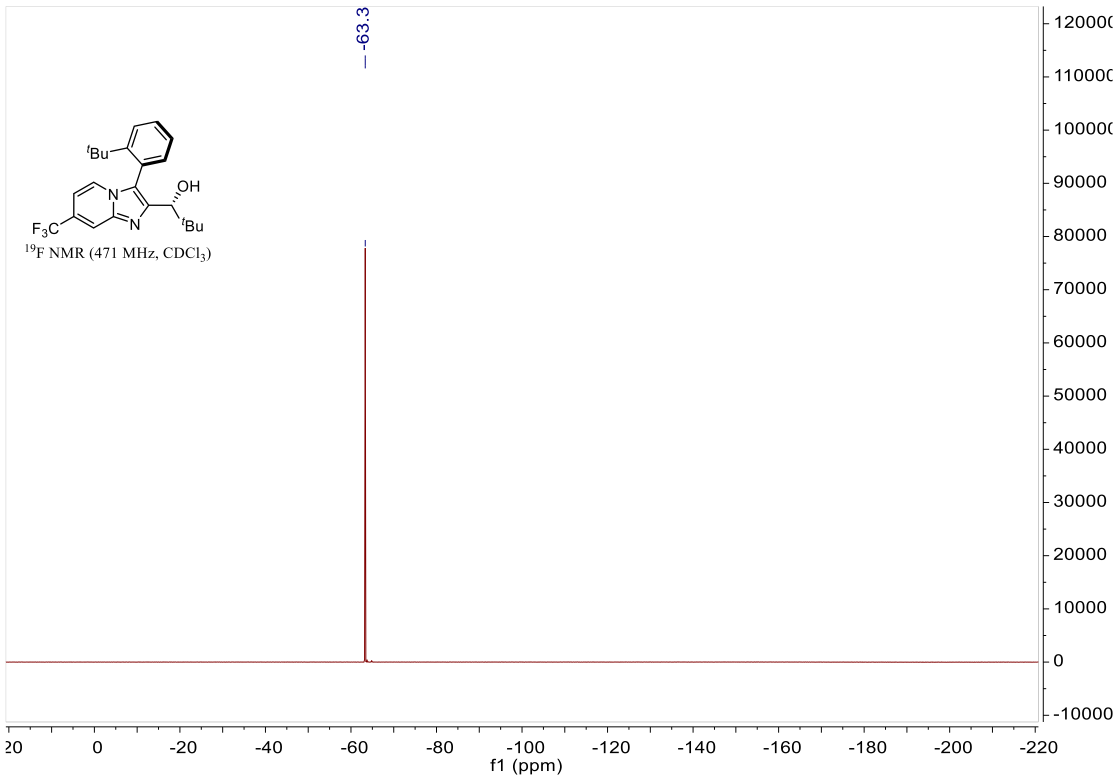
-63.3
tBu
OH
N
N
F3C
tBu
19F NMR (471 MHz, CDCl3)
120000
110000
100000
90000
80000
70000
60000
50000
40000
30000
20000
10000
0
-10000
20
0
-20
-40
-60
-80
-100
-120
-140
-160
-180
-200
-220
f1 (ppm)

Compound **3h**

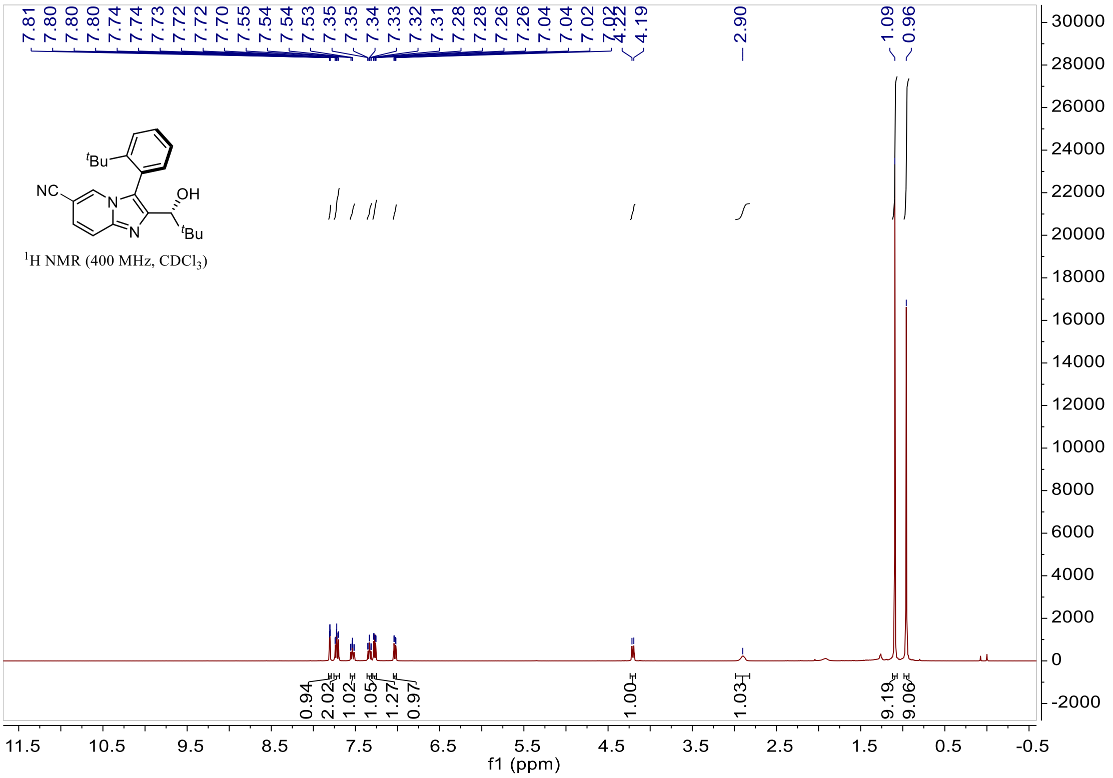

7.81 7.80 7.80 7.80 7.74 7.74 7.73 7.72 7.72 7.70 7.55 7.54 7.54 7.53 7.35 7.35 7.34 7.33 7.32 7.31 7.28 7.28 7.26 7.26 7.04 7.04 7.02 7.02 4.22 4.19
2.90
1.09 0.96
NC
OH
tBu
tBu
N
N
¹H NMR (400 MHz, CDCl₃)
0.94 2.02 1.02 1.05 1.27 0.97
1.00
1.03
9.19 9.06
f1 (ppm)

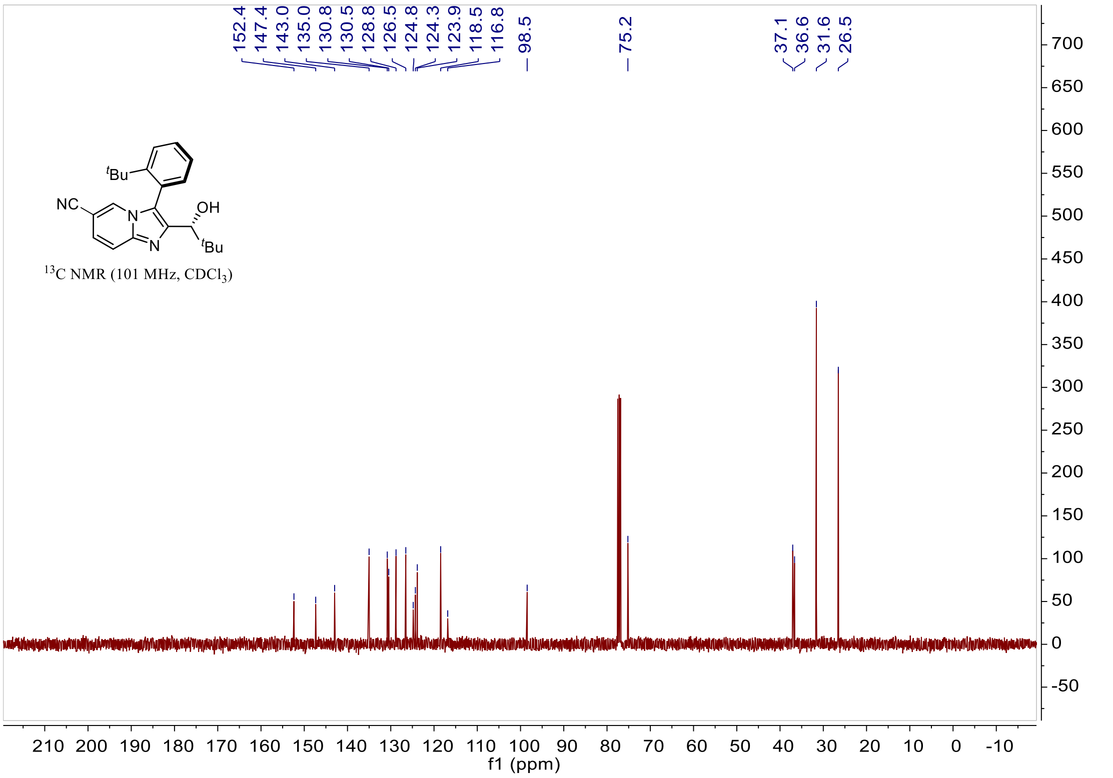

152.4 147.4 143.0 135.0 130.8 130.5 128.8 126.5 124.8 124.3 123.9 118.5 116.8
98.5
75.2
37.1 36.6 31.6 26.5
NC
OH
tBu
tBu
N
N
¹³C NMR (101 MHz, CDCl₃)
f1 (ppm)

Compound **3i**

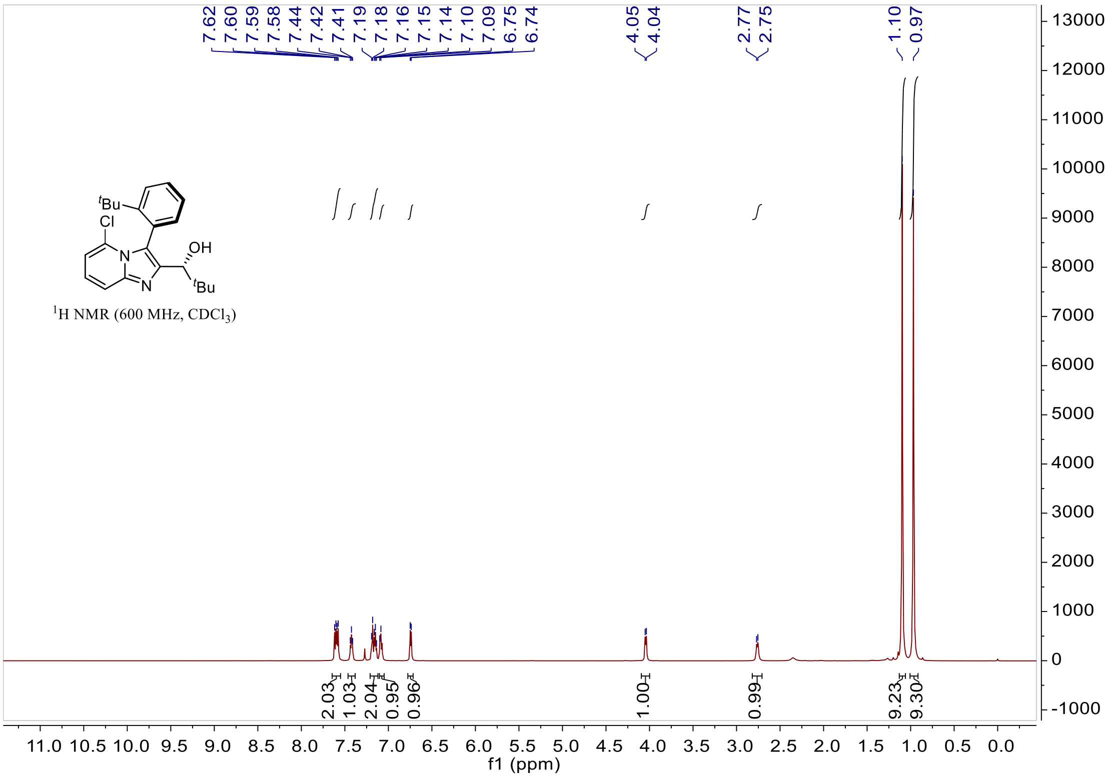
7.62
7.60
7.59
7.58
7.44
7.42
7.41
7.19
7.18
7.16
7.15
7.14
7.10
7.09
6.75
6.74
4.05
4.04
2.77
2.75
1.10
0.97
^{t}Bu
Cl
N
N
OH
^{t}Bu
^{1}H NMR (600 MHz, $CDCl_3$)
2.03
1.03
2.04
0.95
0.96
1.00
0.99
9.23
9.30
f1 (ppm)

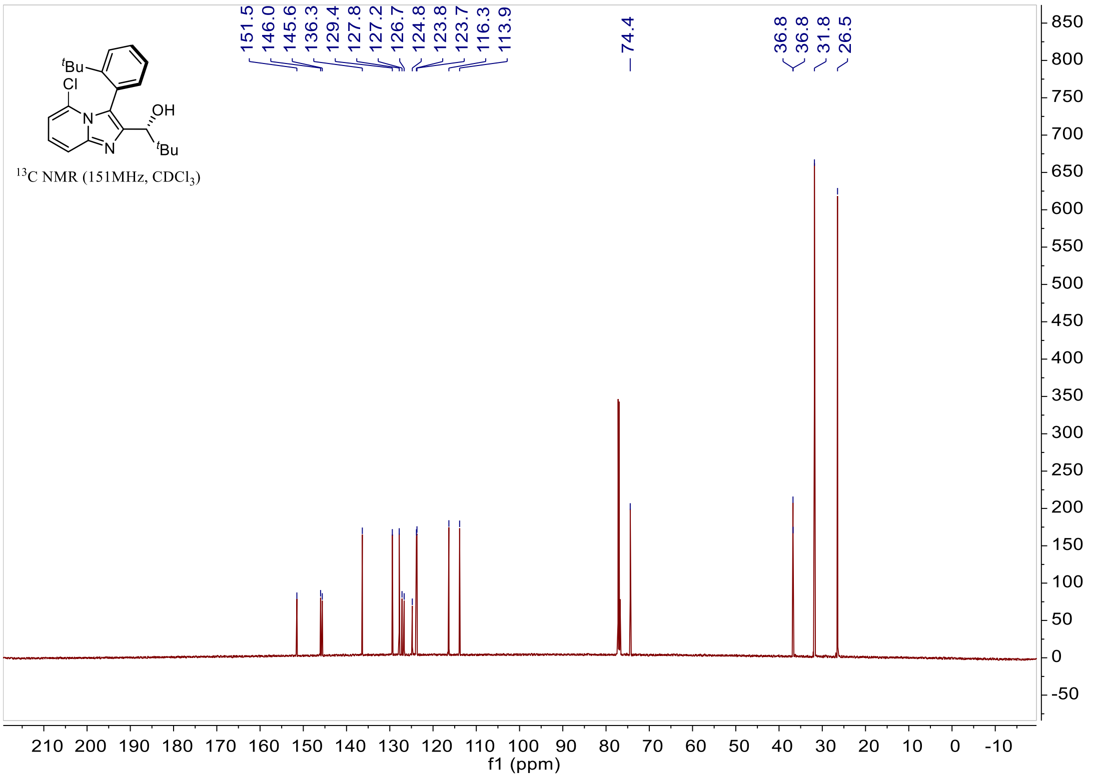
151.5
146.0
145.6
136.3
129.4
127.8
127.2
126.7
124.8
123.8
123.7
116.3
113.9
74.4
36.8
36.8
31.8
26.5
^{t}Bu
Cl
N
N
OH
^{t}Bu
^{13}C NMR (151MHz, $CDCl_3$)
f1 (ppm)

Compound **3j**

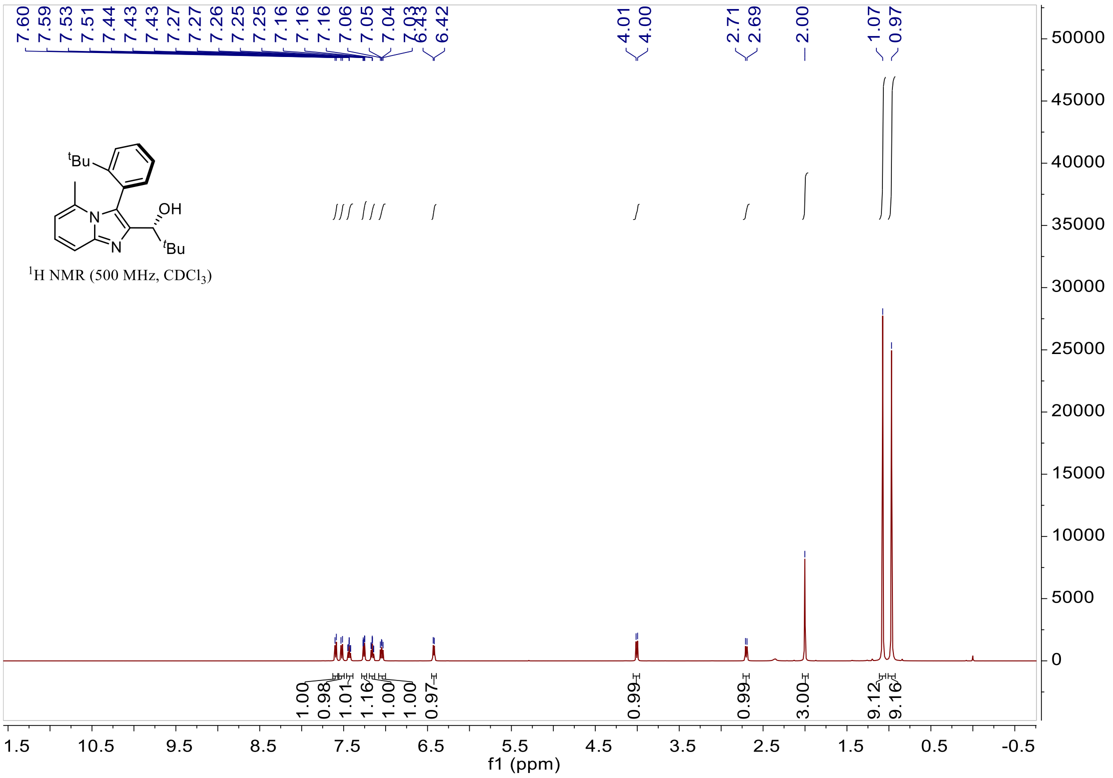
7.60
7.59
7.53
7.51
7.44
7.43
7.43
7.27
7.27
7.26
7.25
7.25
7.16
7.16
7.16
7.06
7.05
7.04
7.03
6.43
6.42
4.01
4.00
2.71
2.69
2.00
1.07
0.97
tBu
OH
N
tBu
1H NMR (500 MHz, CDCl3)
1.00
0.98
1.01
1.16
1.00
1.00
0.97
0.99
0.99
3.00
9.12
9.16
f1 (ppm)

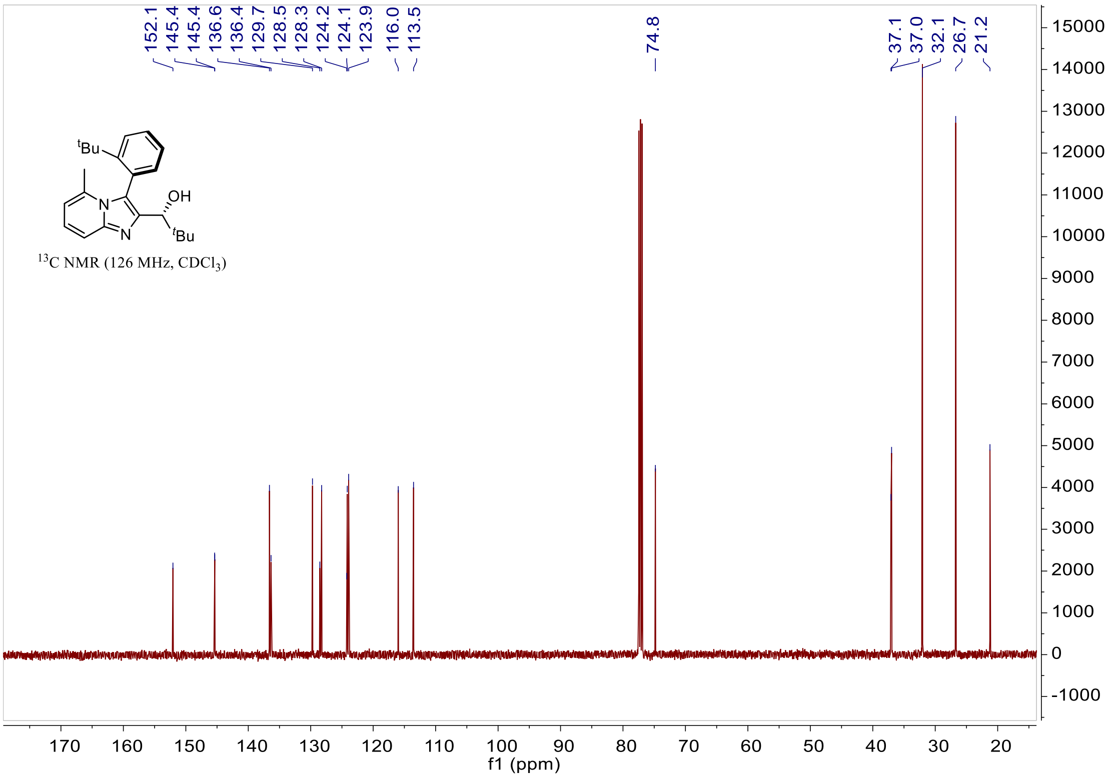
152.1
145.4
145.4
136.6
136.4
129.7
128.5
128.3
124.2
124.1
123.9
116.0
113.5
74.8
37.1
37.0
32.1
26.7
21.2
tBu
OH
N
tBu
13C NMR (126 MHz, CDCl3)
f1 (ppm)

Compound **3k**

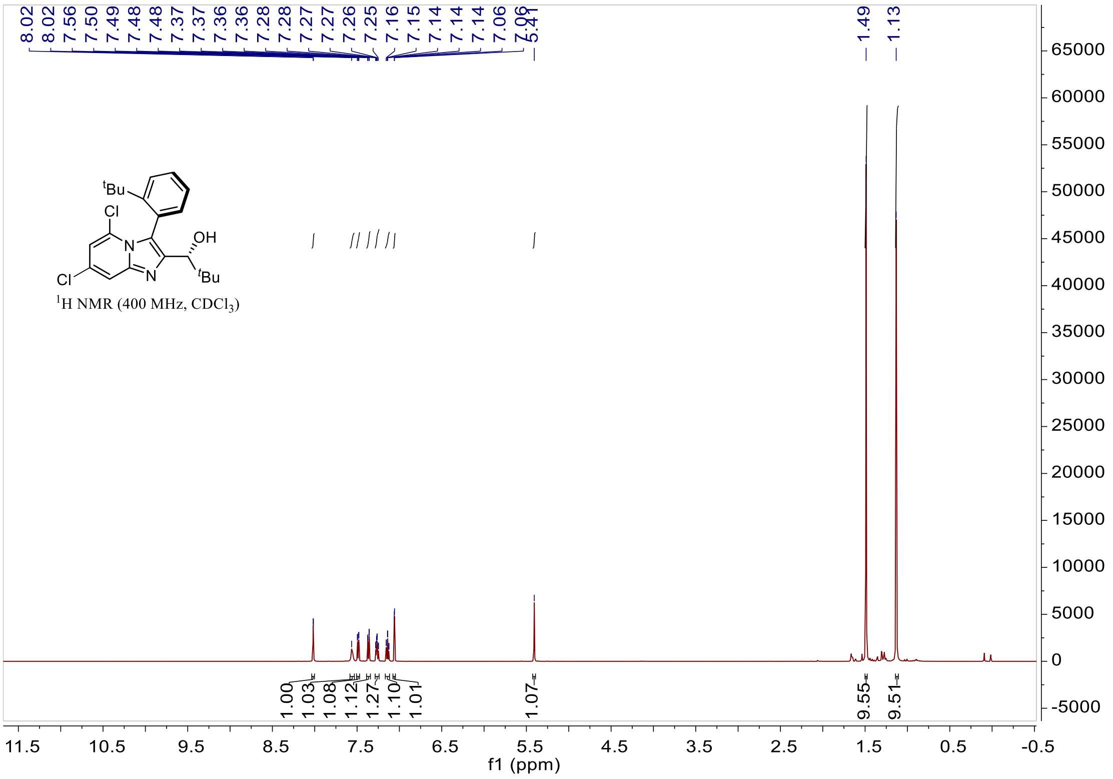

8.02
8.02
7.56
7.50
7.49
7.48
7.48
7.37
7.37
7.36
7.36
7.28
7.28
7.27
7.27
7.26
7.25
7.16
7.15
7.14
7.14
7.14
7.06
7.06
5.41
1.49
1.13
tBu
Cl
OH
Cl
N
N
tBu
1H NMR (400 MHz, CDCl3)
1.00
1.03
1.08
1.12
1.27
1.10
1.01
1.07
9.55
9.51
f1 (ppm)

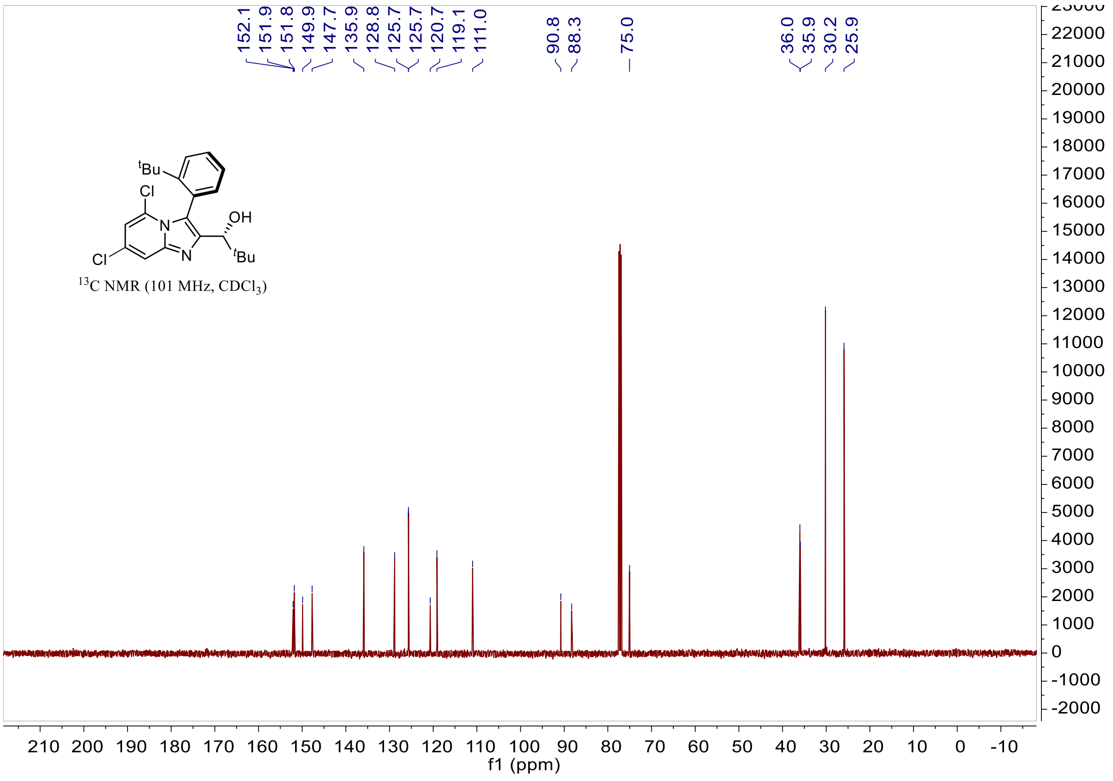

152.1
151.9
151.8
149.9
147.7
135.9
128.8
125.7
125.7
120.7
119.1
111.0
90.8
88.3
75.0
36.0
35.9
30.2
25.9
tBu
Cl
OH
Cl
N
N
tBu
13C NMR (101 MHz, CDCl3)
f1 (ppm)

Compound **3l**

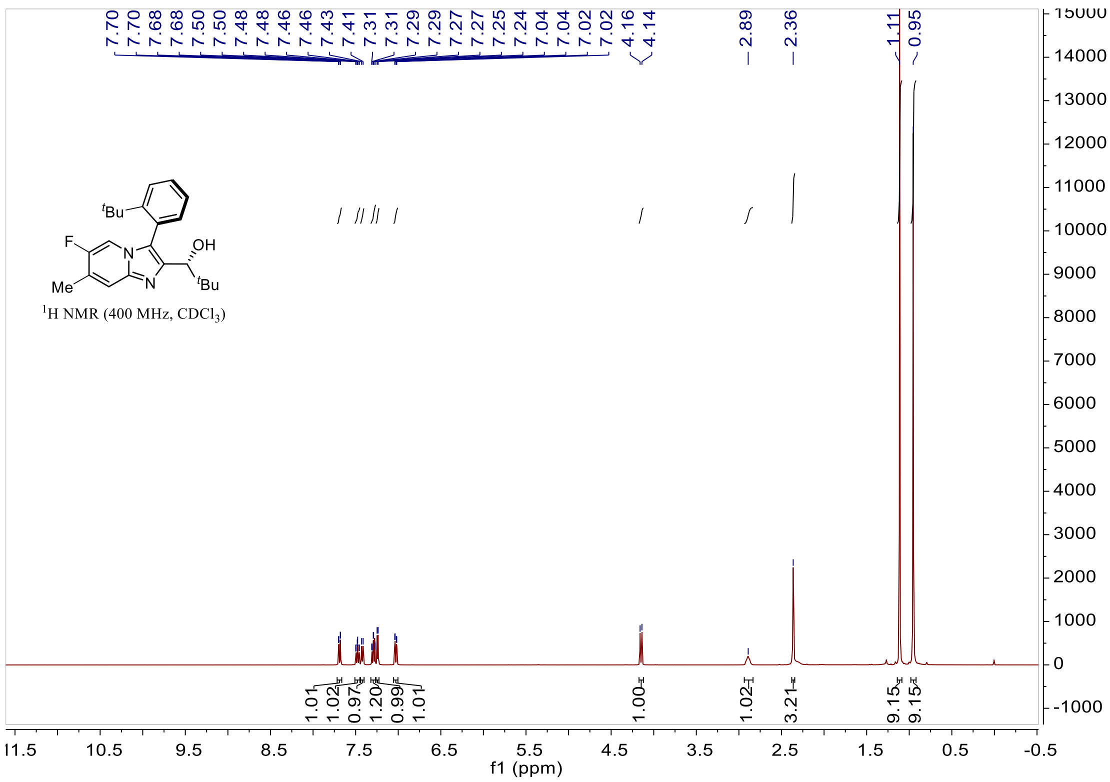

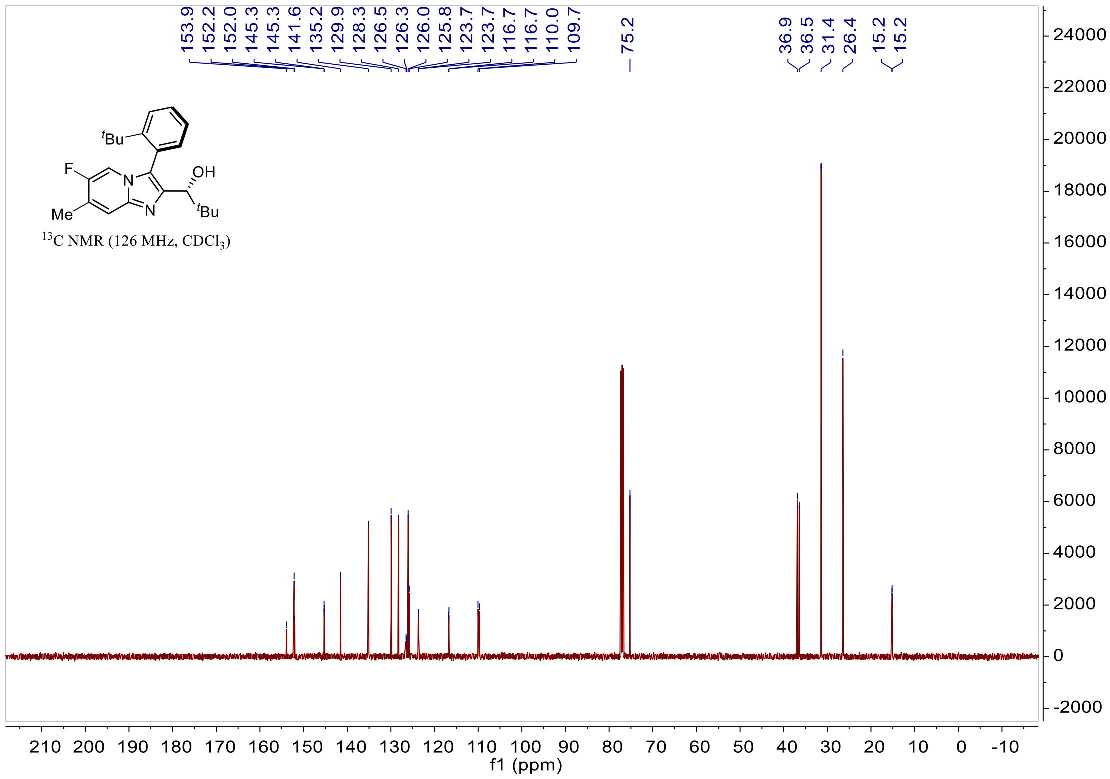

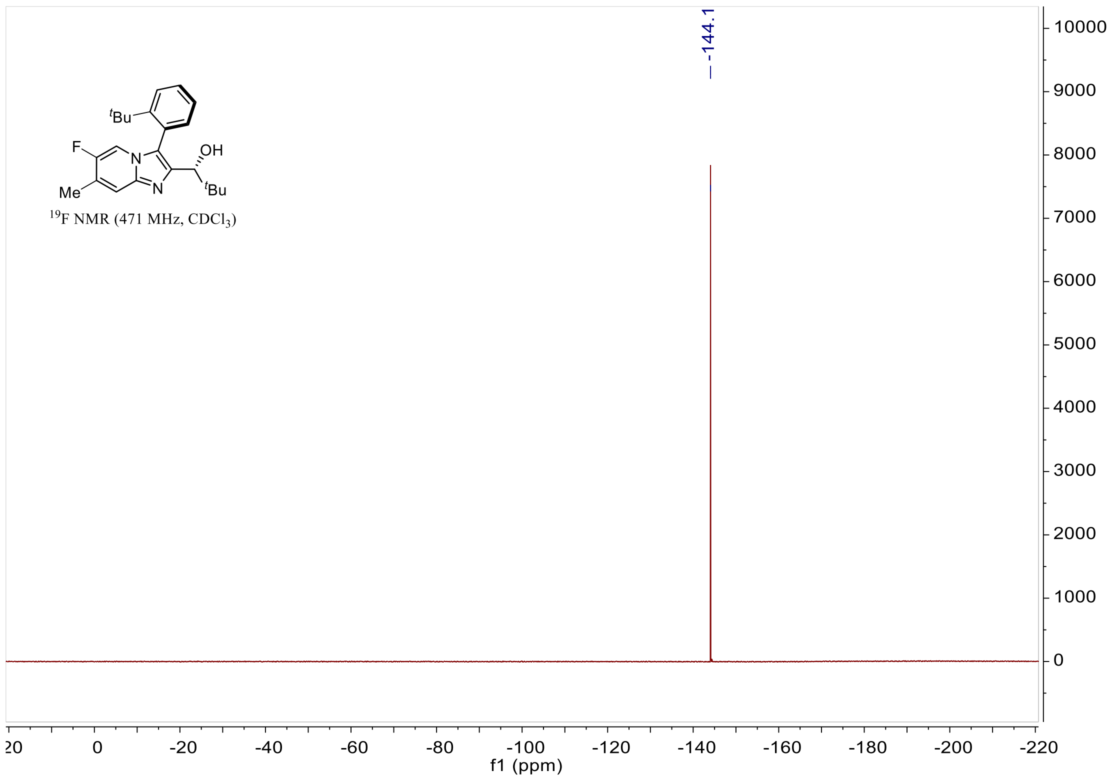

-144.1
tBu
F
OH
Me
N
N
tBu
19F NMR (471 MHz, CDCl3)
10000
9000
8000
7000
6000
5000
4000
3000
2000
1000
0
20
0
-20
-40
-60
-80
-100
-120
-140
-160
-180
-200
-220
f1 (ppm)

Compound **3m**

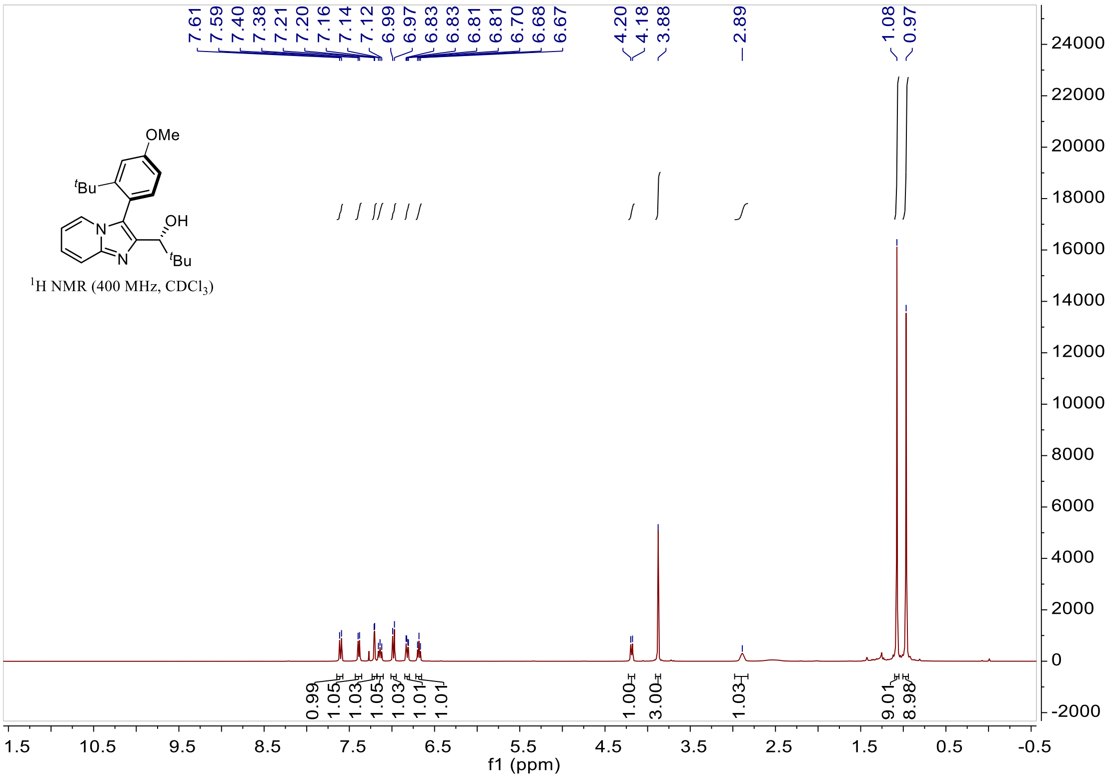

OMe
tBu
OH
N
tBu
1H NMR (400 MHz, CDCl3)
7.61 7.59 7.40 7.38 7.21 7.20 7.16 7.14 7.12 6.99 6.97 6.83 6.83 6.81 6.81 6.70 6.68 6.67
4.20 4.18 3.88
2.89
1.08 0.97
0.99 1.05 1.03 1.05 1.03 1.01 1.01
1.00 3.00
1.03
9.01 8.98
f1 (ppm)

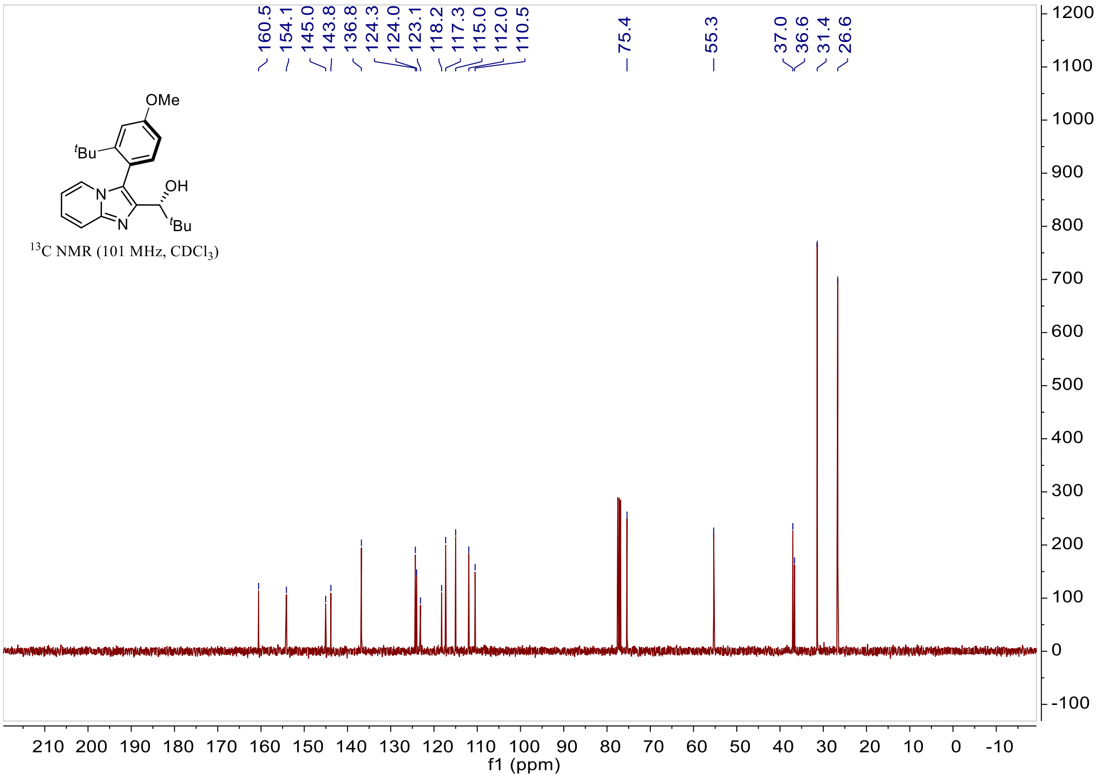

OMe
tBu
OH
N
tBu
13C NMR (101 MHz, CDCl3)
160.5 154.1 145.0 143.8 136.8 124.3 124.0 123.1 118.2 117.3 115.0 112.0 110.5
75.4
55.3
37.0 36.6 31.4 26.6
f1 (ppm)

Compound **3m'**

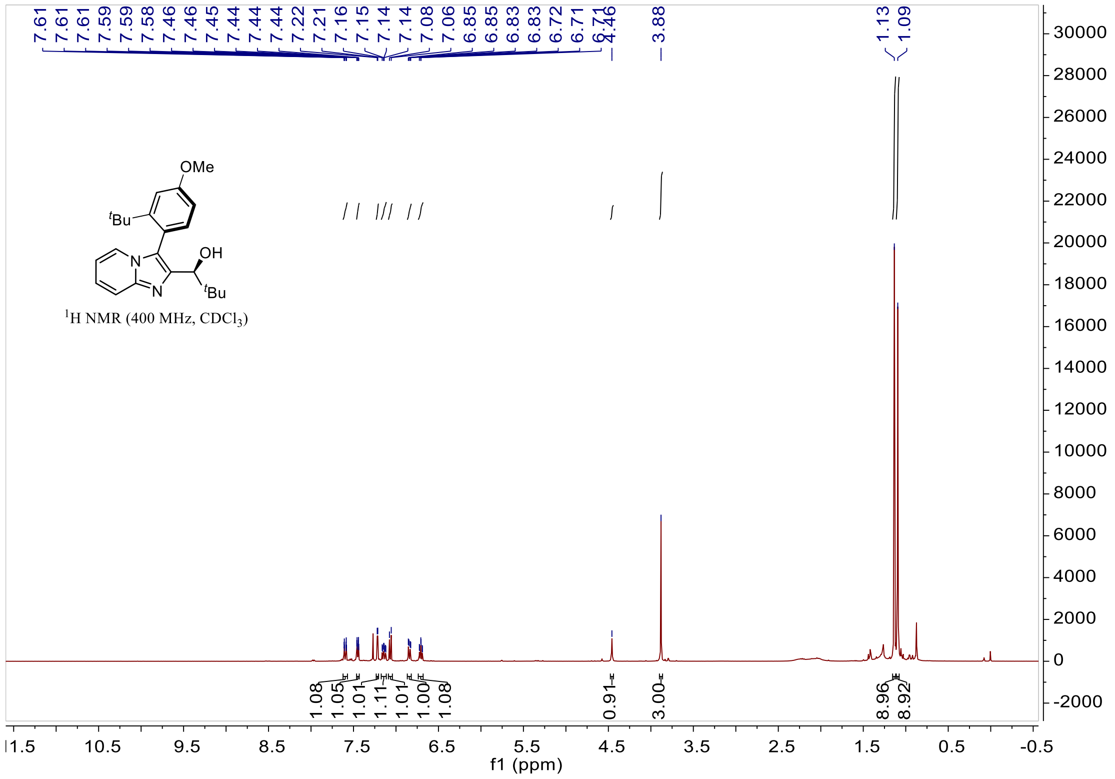

7.61 7.61 7.61 7.59 7.59 7.58 7.46 7.46 7.45 7.44 7.44 7.44 7.22 7.21 7.16 7.15 7.14 7.14 7.08 7.06 6.85 6.85 6.83 6.83 6.72 6.71 6.71 4.46
3.88
1.13 1.09
OMe
tBu
OH
N
N
tBu
1H NMR (400 MHz, CDCl3)
1.08 1.05 1.01 1.11 1.01 1.00 1.08
0.91
3.00
8.96 8.92
f1 (ppm)

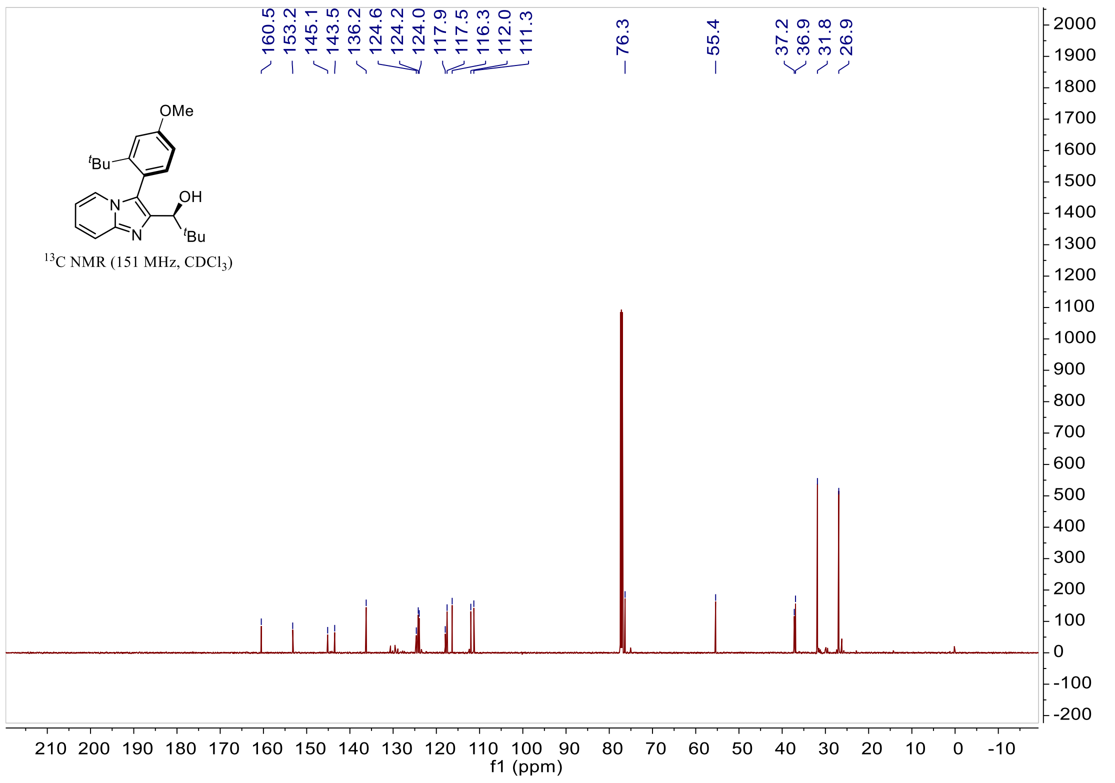

160.5 153.2 145.1 143.5 136.2 124.6 124.2 124.0 117.9 117.5 116.3 112.0 111.3
76.3
55.4
37.2 36.9 31.8 26.9
OMe
tBu
OH
N
N
tBu
13C NMR (151 MHz, CDCl3)
f1 (ppm)

Compound **3n**

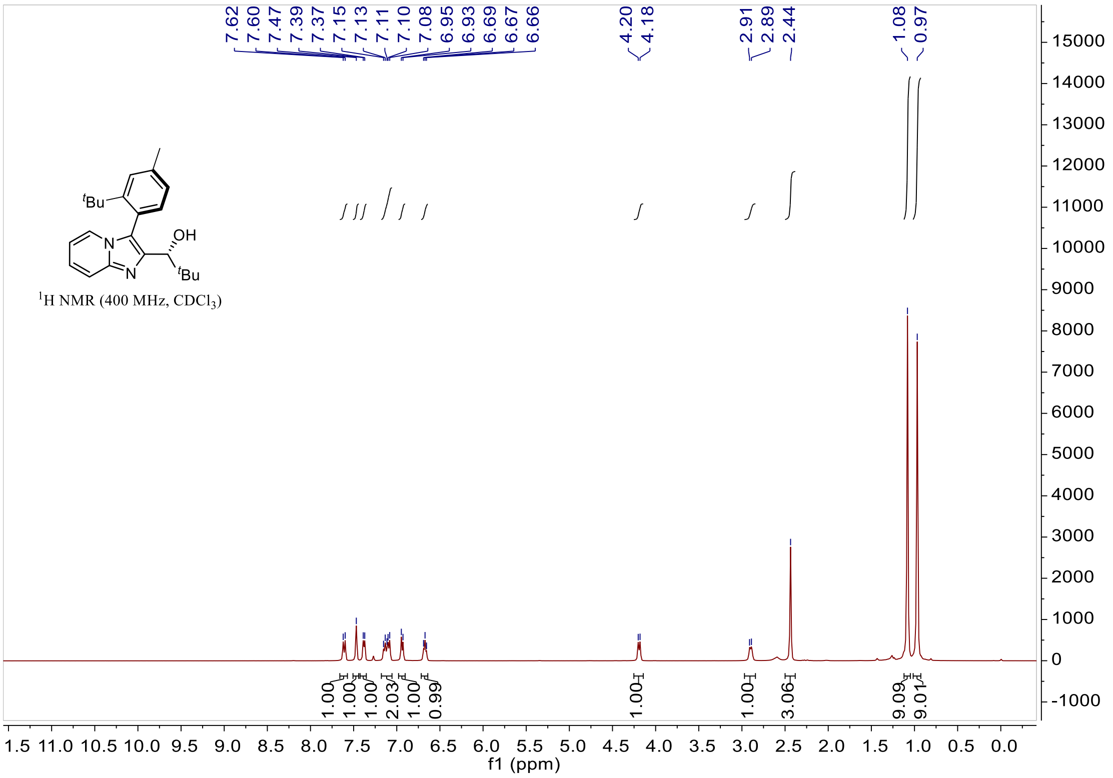

7.62
7.60
7.47
7.39
7.37
7.15
7.13
7.11
7.10
7.08
6.95
6.93
6.69
6.67
6.66
4.20
4.18
2.91
2.89
2.44
1.08
0.97
^{1}H NMR (400 MHz, CDCl$_3$)
OH
tBu
N
1.00
1.00
1.00
2.03
1.00
0.99
1.00
1.00
3.06
9.09
9.01
f1 (ppm)

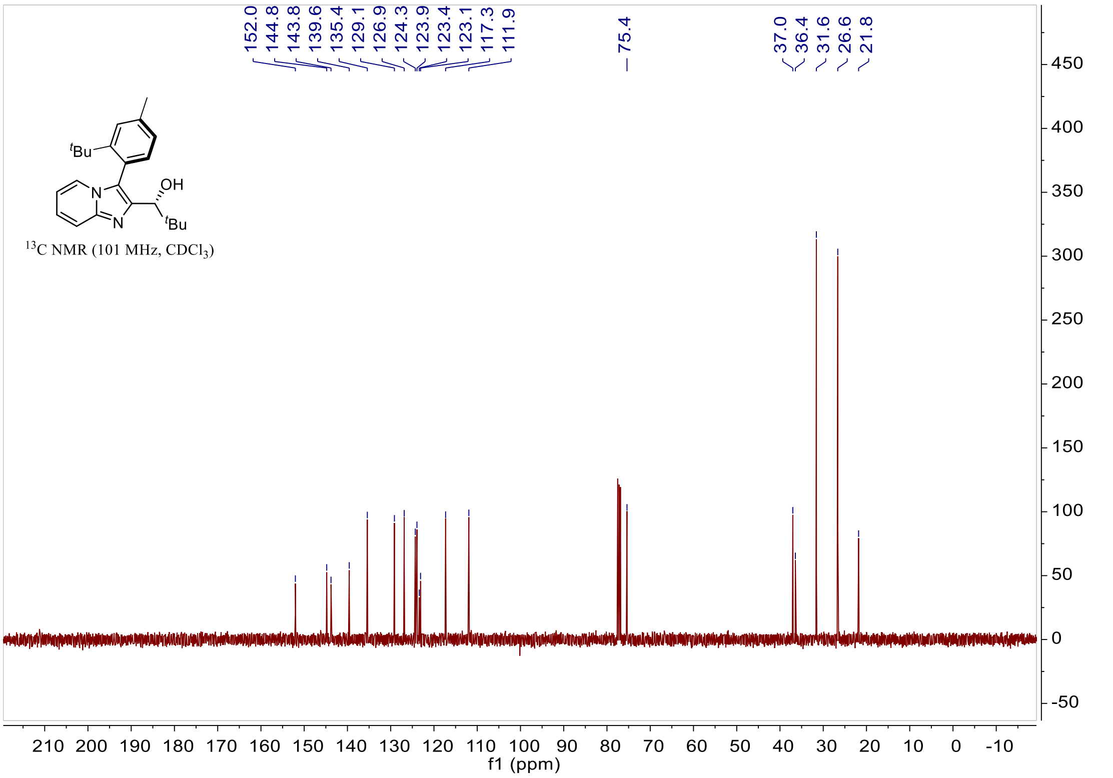

152.0
144.8
143.8
139.6
135.4
129.1
126.9
124.3
123.9
123.4
123.1
117.3
111.9
75.4
37.0
36.4
31.6
26.6
21.8
^{13}C NMR (101 MHz, CDCl$_3$)
OH
tBu
N
f1 (ppm)

Compound **3n'**

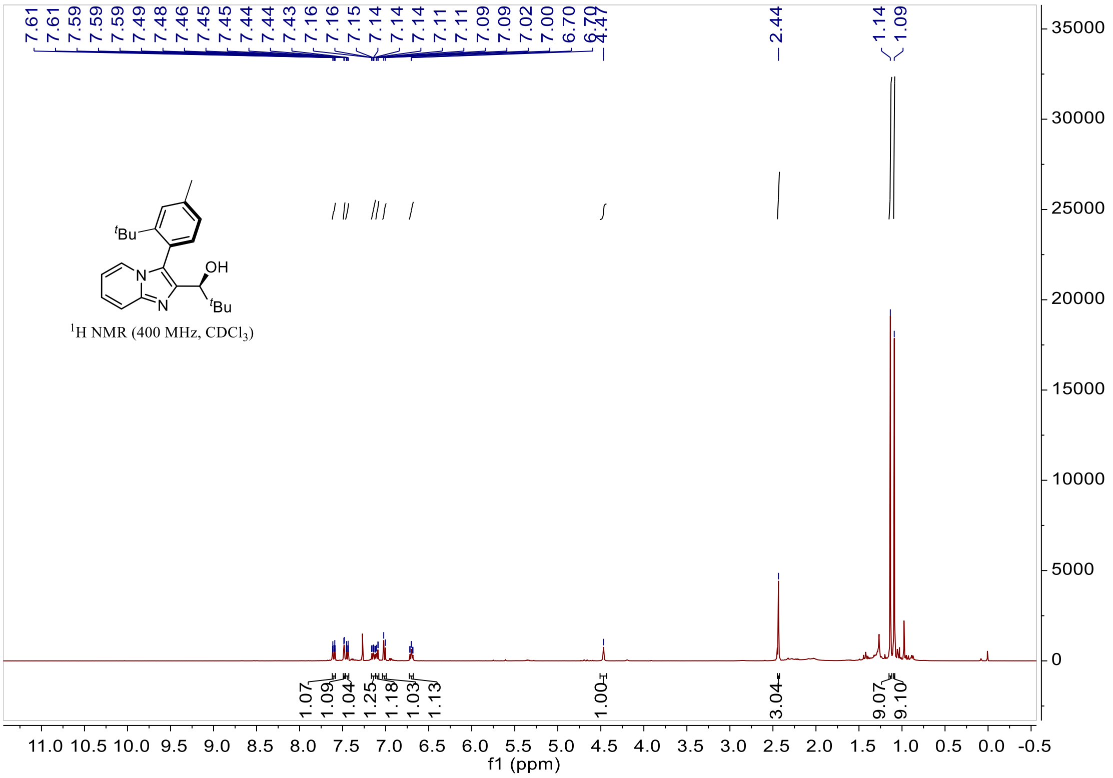

7.61 7.61 7.59 7.59 7.59 7.49 7.48 7.46 7.45 7.44 7.44 7.43 7.16 7.15 7.14 7.14 7.11 7.11 7.09 7.09 7.02 7.00 6.70 6.70 4.47
2.44
1.14 1.09
tBu
OH
N
N
tBu
1H NMR (400 MHz, CDCl3)
1.07 1.09 1.04 1.25 1.18 1.03 1.13
1.00
3.04
9.07 9.10
f1 (ppm)

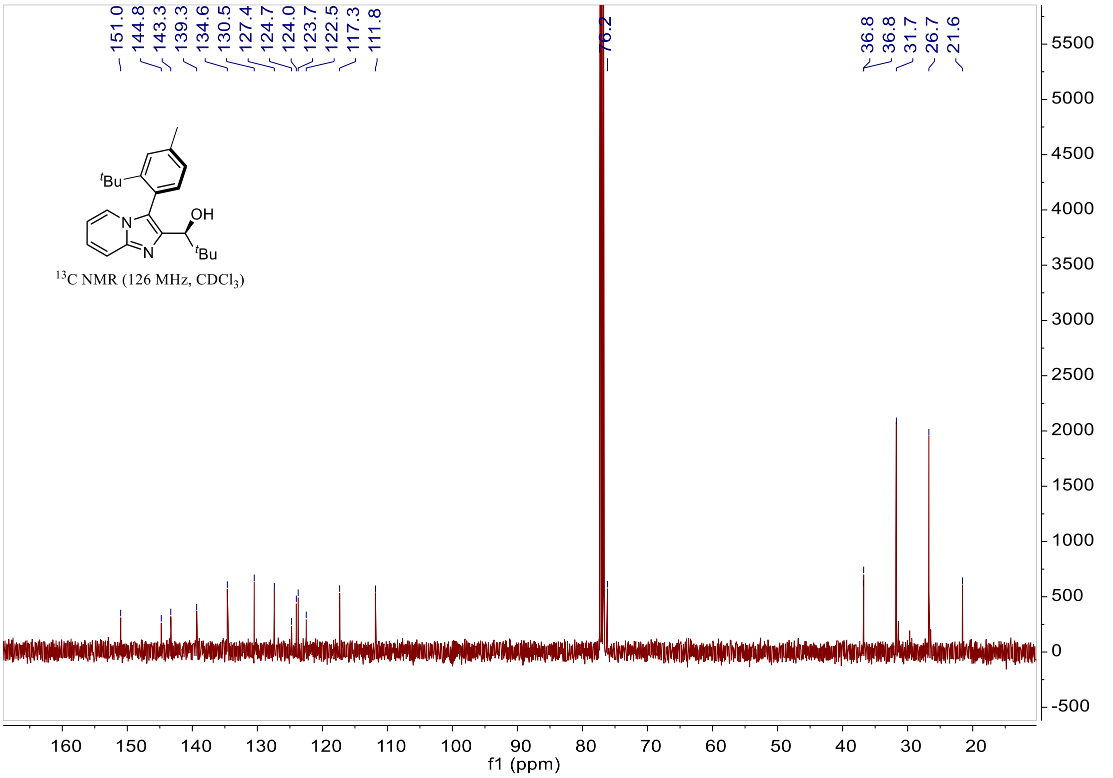

151.0 144.8 143.3 139.3 134.6 130.5 127.4 124.7 124.0 123.7 122.5 117.3 111.8
76.2
36.8 36.8 31.7 26.7 21.6
tBu
OH
N
N
tBu
13C NMR (126 MHz, CDCl3)
f1 (ppm)

Compound **3o**

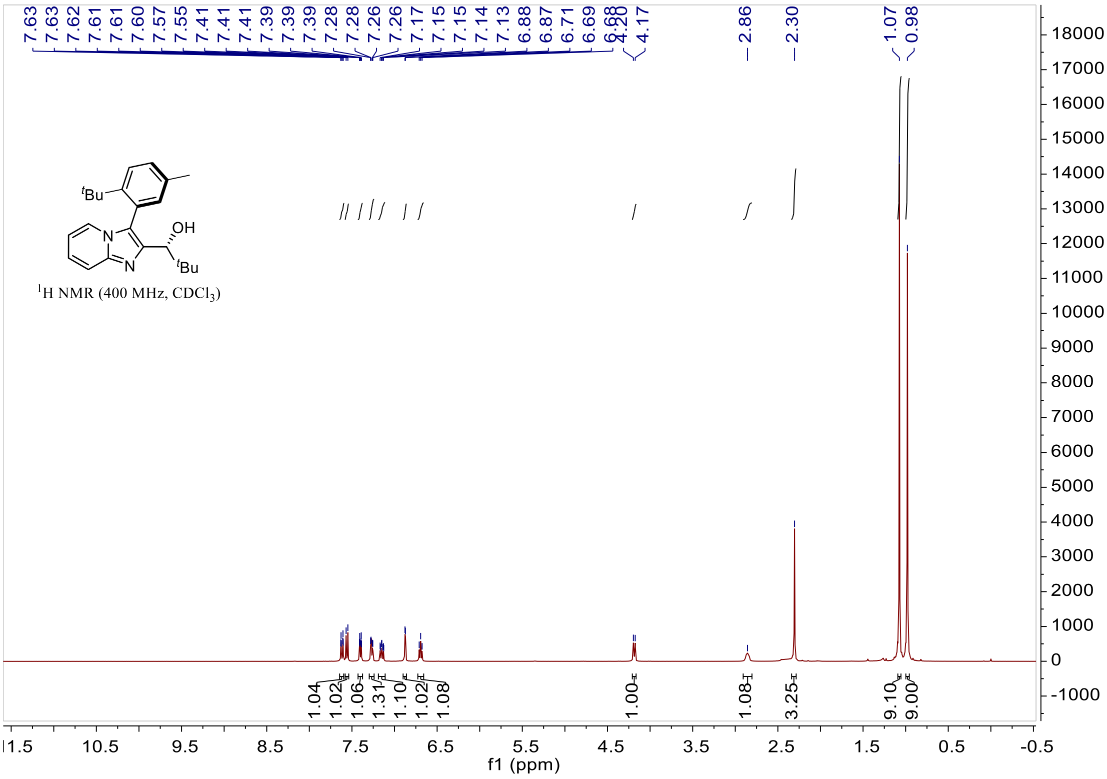

7.63
7.63
7.62
7.61
7.61
7.60
7.57
7.55
7.41
7.41
7.41
7.39
7.39
7.39
7.28
7.28
7.26
7.26
7.17
7.15
7.15
7.14
7.13
6.88
6.87
6.71
6.69
6.68
4.20
4.17
2.86
2.30
1.07
0.98
tBu
OH
N
N
tBu
1H NMR (400 MHz, CDCl3)
1.04
1.02
1.06
1.31
1.10
1.02
1.08
1.00
1.08
3.25
9.10
9.00
f1 (ppm)

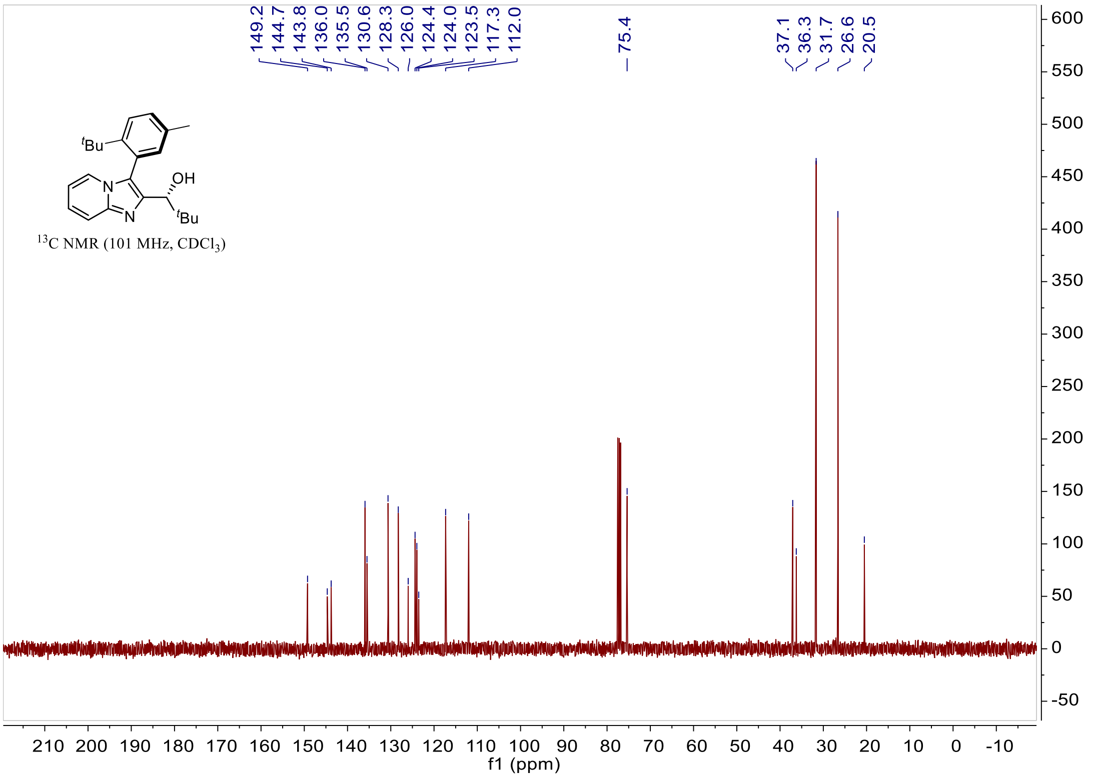

149.2
144.7
143.8
136.0
135.5
130.6
128.3
126.0
124.4
124.0
123.5
117.3
112.0
75.4
37.1
36.3
31.7
26.6
20.5
tBu
OH
N
N
tBu
13C NMR (101 MHz, CDCl3)
f1 (ppm)

Compound **3p**

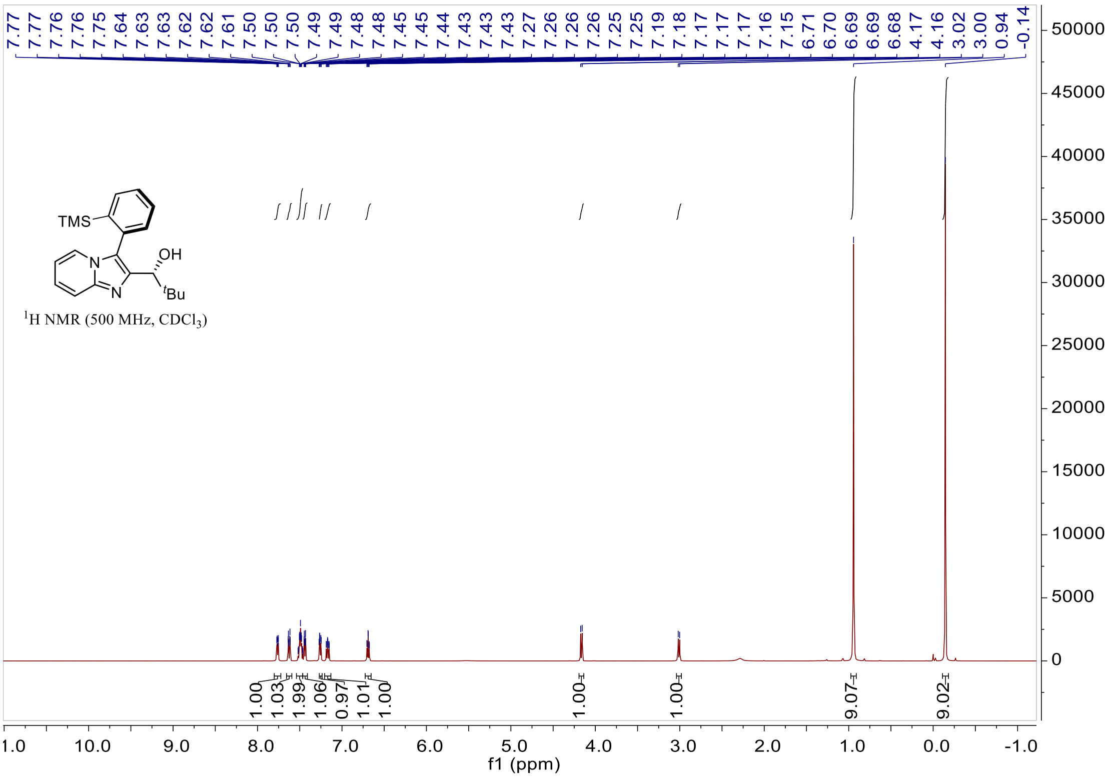
TMS
OH
N
N
^tBu
^1H NMR (500 MHz, $CDCl_3$)
f1 (ppm)

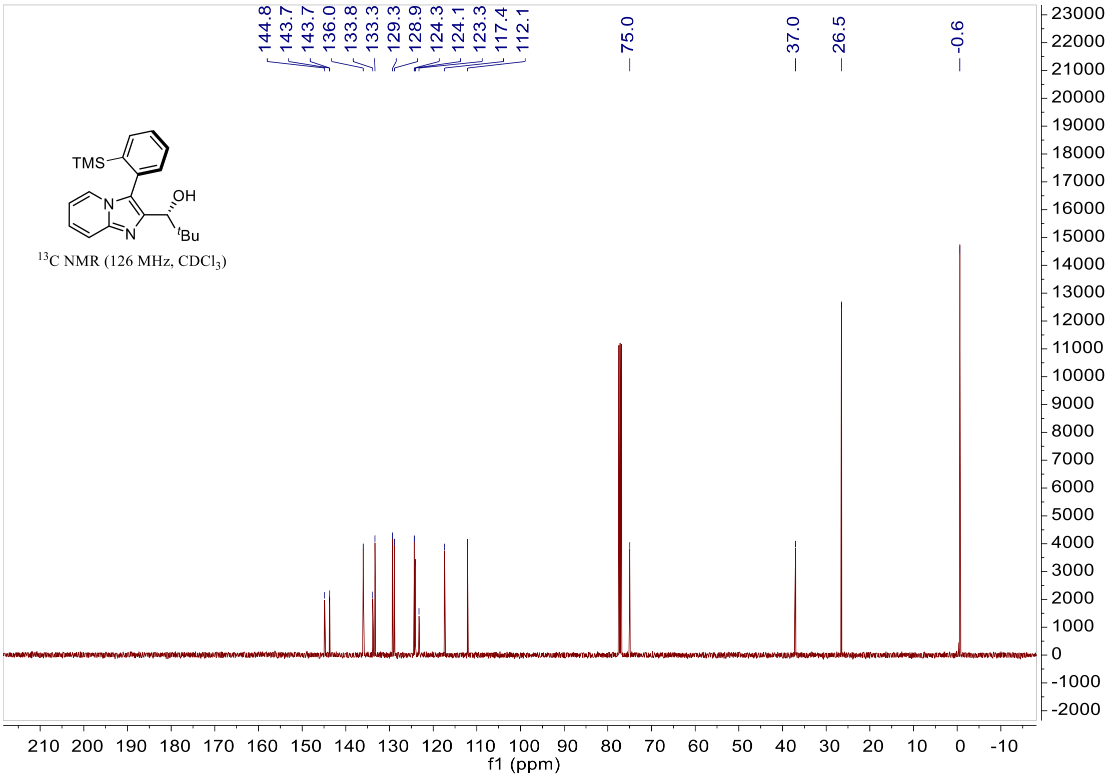
TMS
OH
N
N
^tBu
^{13}C NMR (126 MHz, $CDCl_3$)
f1 (ppm)

Compound **3q**

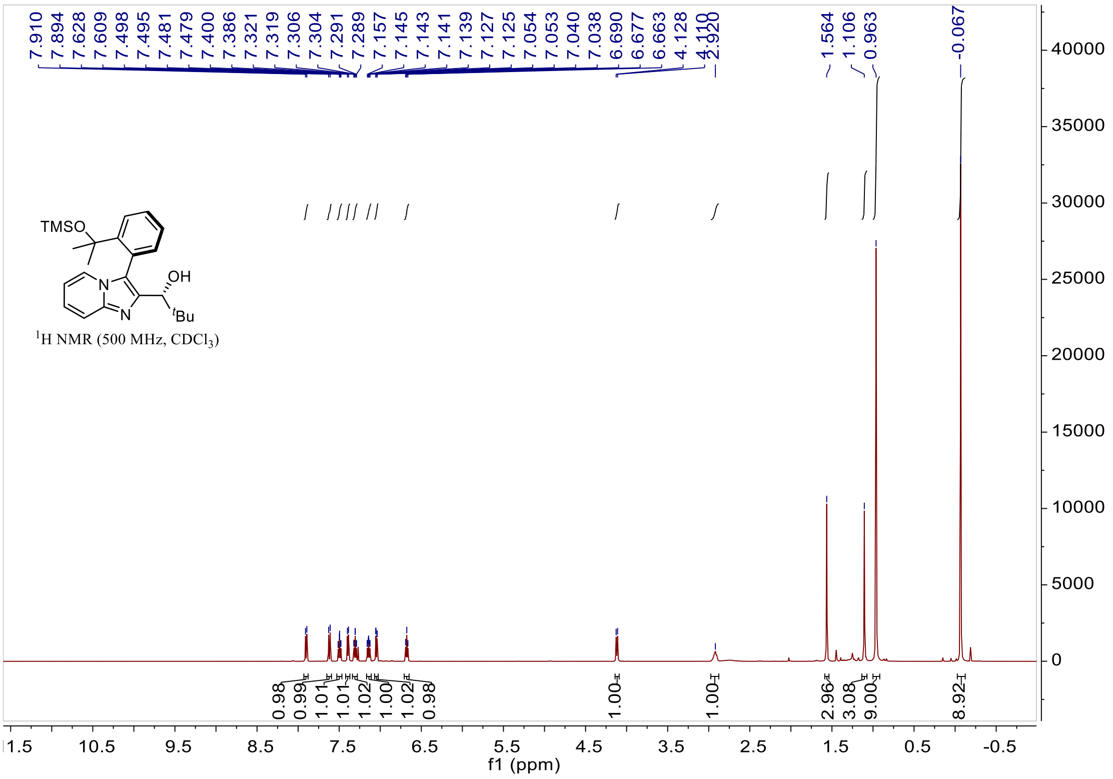

7.910
7.894
7.628
7.609
7.498
7.495
7.481
7.479
7.400
7.386
7.321
7.319
7.306
7.304
7.291
7.289
7.157
7.145
7.143
7.141
7.139
7.127
7.125
7.054
7.053
7.040
7.038
6.690
6.677
6.663
4.128
4.110
2.920
1.564
1.106
0.963
-0.067
TMSO
OH
N
N
tBu
1H NMR (500 MHz, CDCl3)
0.98
0.99
1.01
1.01
1.02
1.00
1.02
0.98
1.00
1.00
2.96
3.08
9.00
8.92
f1 (ppm)

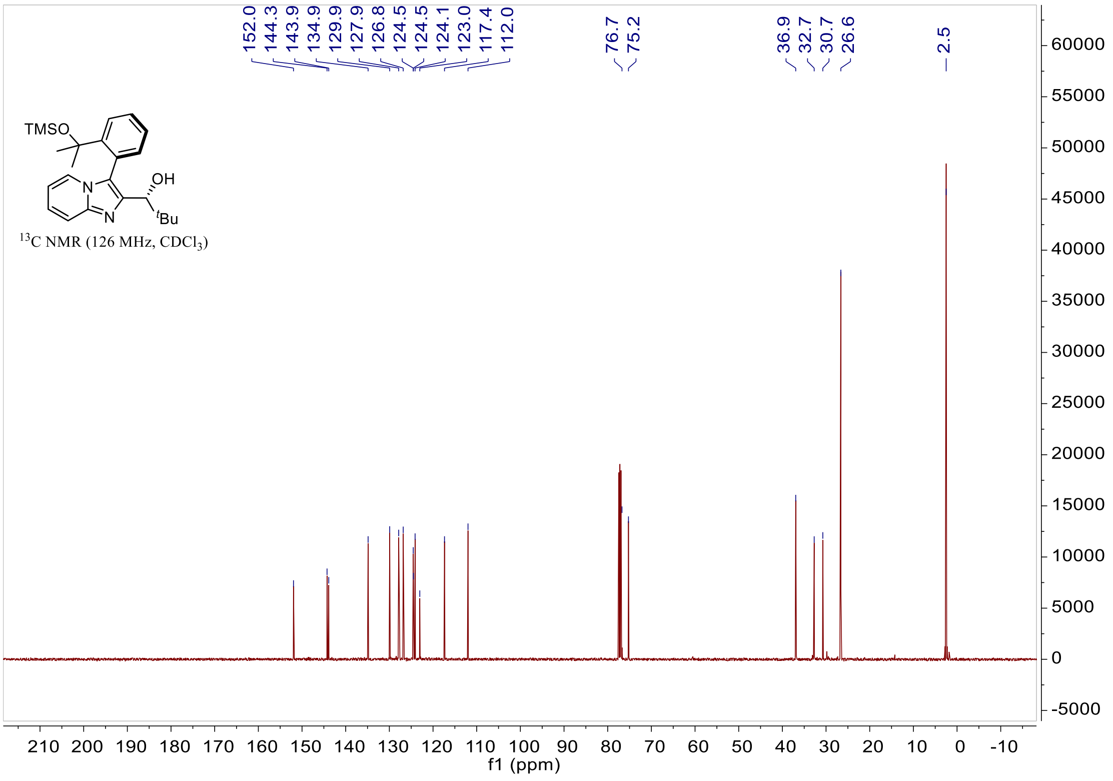

152.0
144.3
143.9
134.9
129.9
127.9
126.8
124.5
124.5
124.1
123.0
117.4
112.0
76.7
75.2
36.9
32.7
30.7
26.6
2.5
TMSO
OH
N
N
tBu
13C NMR (126 MHz, CDCl3)
f1 (ppm)

Compound **3r**

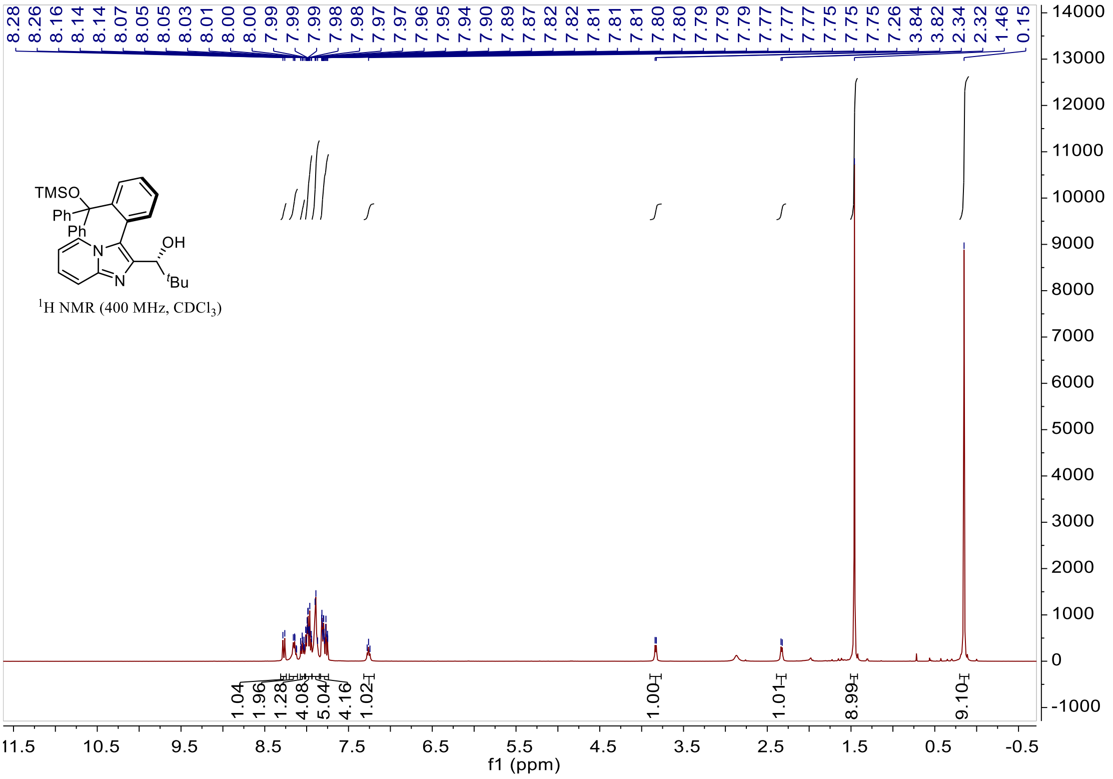
TMSO
Ph
Ph
N
OH
N
tBu
1H NMR (400 MHz, CDCl3)
8.28 8.26 8.16 8.14 8.14 8.07 8.05 8.03 8.01 8.00 7.99 7.99 7.99 7.98 7.97 7.97 7.96 7.95 7.94 7.90 7.89 7.87 7.82 7.82 7.81 7.81 7.80 7.80 7.79 7.79 7.77 7.77 7.77 7.75 7.75 7.75 7.26 3.84 3.82 2.34 2.32 1.46 0.15
1.04 1.96 1.28 4.08 5.04 4.16 1.02 1.00 1.01 8.99 9.10
f1 (ppm)

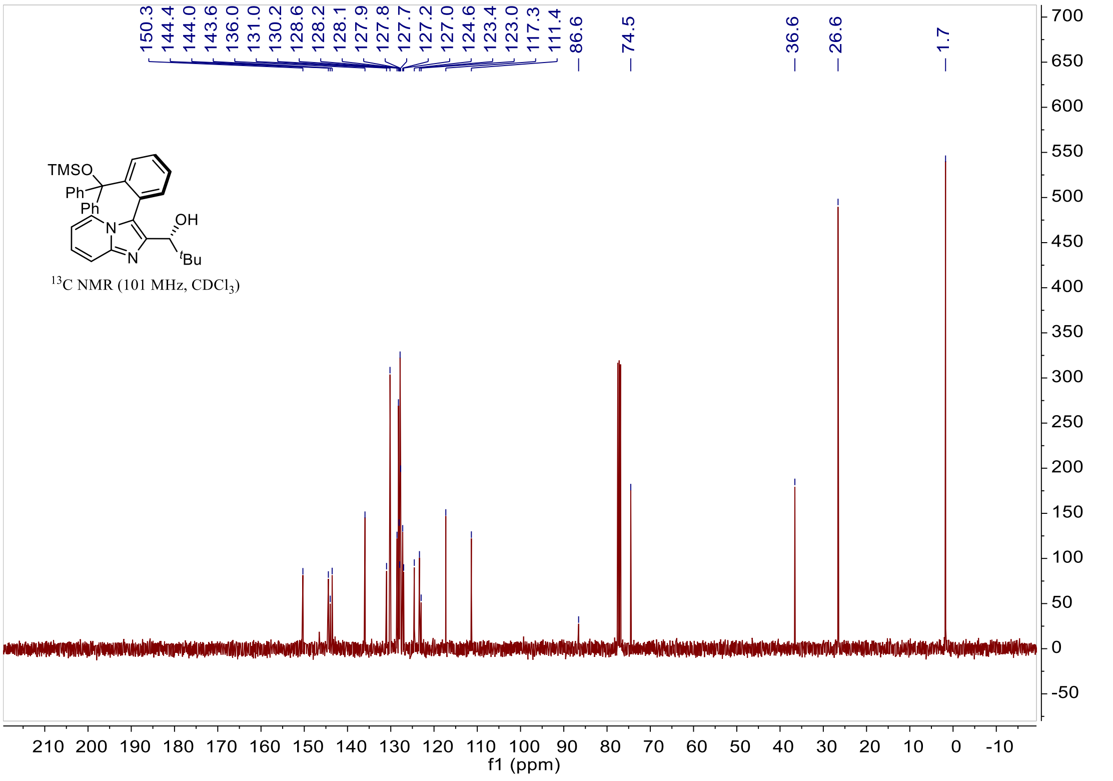
TMSO
Ph
Ph
N
OH
N
tBu
13C NMR (101 MHz, CDCl3)
150.3 144.4 144.0 143.6 136.0 131.0 130.2 128.6 128.2 128.1 127.9 127.8 127.7 127.2 127.0 124.6 123.4 123.0 117.3 111.4 86.6 74.5 36.6 26.6 1.7
f1 (ppm)

Compound **3s**

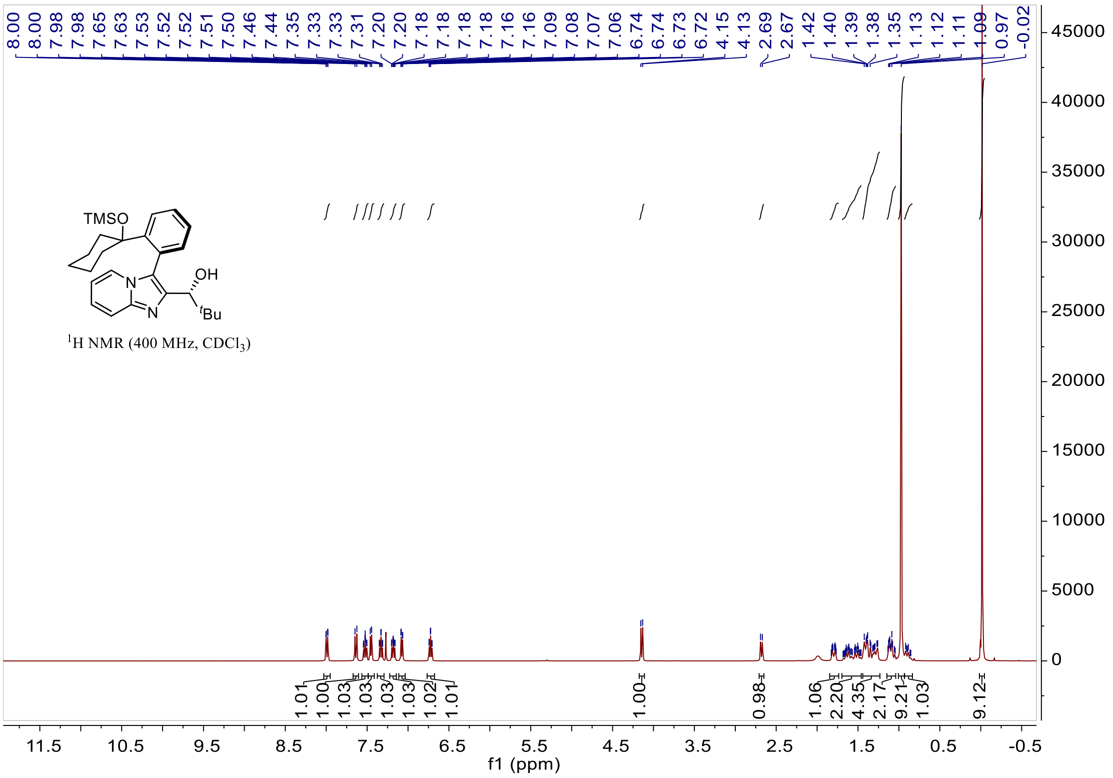

TMSO
OH
tBu
N
N
1H NMR (400 MHz, CDCl3)
8.00 8.00 7.98 7.98 7.65 7.63 7.53 7.52 7.52 7.51 7.50 7.46 7.44 7.35 7.33 7.33 7.31 7.20 7.20 7.18 7.18 7.18 7.16 7.16 7.09 7.08 7.07 7.06 6.74 6.74 6.73 6.72 4.15 4.13 2.69 2.67 1.42 1.40 1.39 1.38 1.35 1.13 1.12 1.11 1.09 0.97 -0.02
1.01 1.00 1.03 1.03 1.03 1.03 1.02 1.01 1.00 0.98 1.06 2.20 4.35 2.17 9.21 1.03 9.12
f1 (ppm)

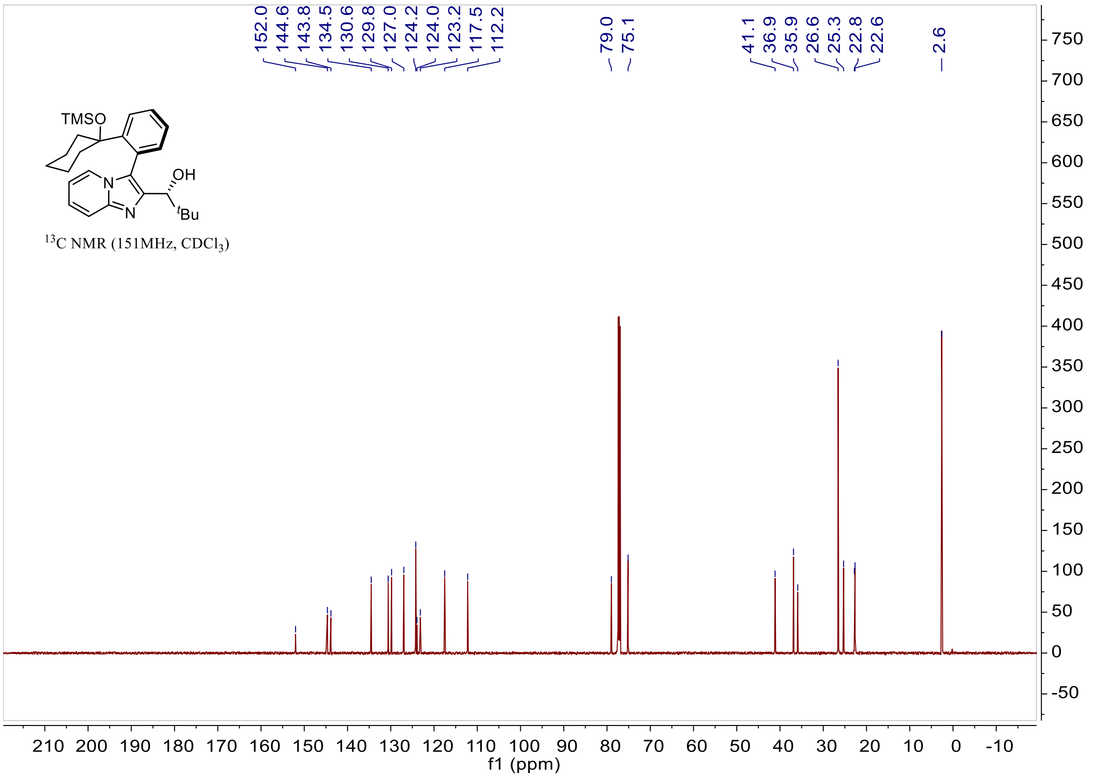

TMSO
OH
tBu
N
N
13C NMR (151MHz, CDCl3)
152.0 144.6 143.8 134.5 130.6 129.8 127.0 124.2 124.0 123.2 117.5 112.2 79.0 75.1 41.1 36.9 35.9 26.6 25.3 22.8 22.6 2.6
f1 (ppm)

Compound **3t**

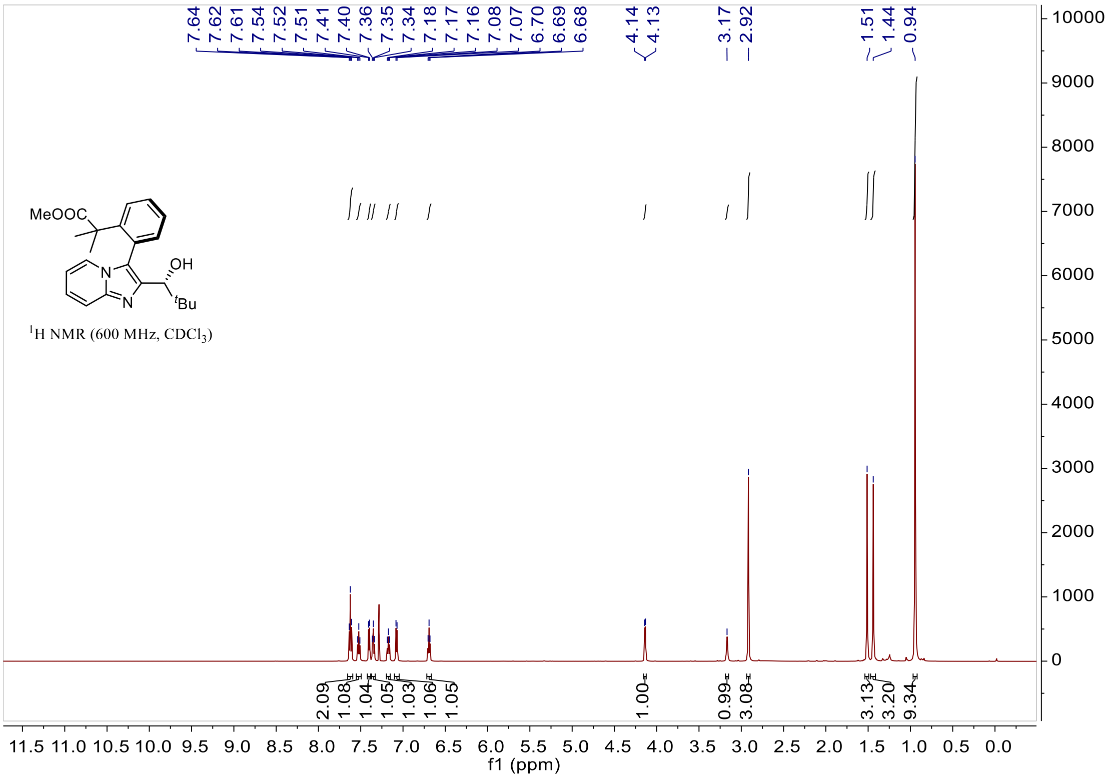
7.64
7.62
7.61
7.54
7.52
7.51
7.41
7.40
7.36
7.35
7.34
7.18
7.17
7.16
7.08
7.07
6.70
6.69
6.68
4.14
4.13
3.17
2.92
1.51
1.44
0.94
MeOOC
OH
N
N
tBu
1H NMR (600 MHz, CDCl3)
2.09
1.08
1.04
1.05
1.03
1.06
1.05
1.00
0.99
3.08
3.13
3.20
9.34
f1 (ppm)

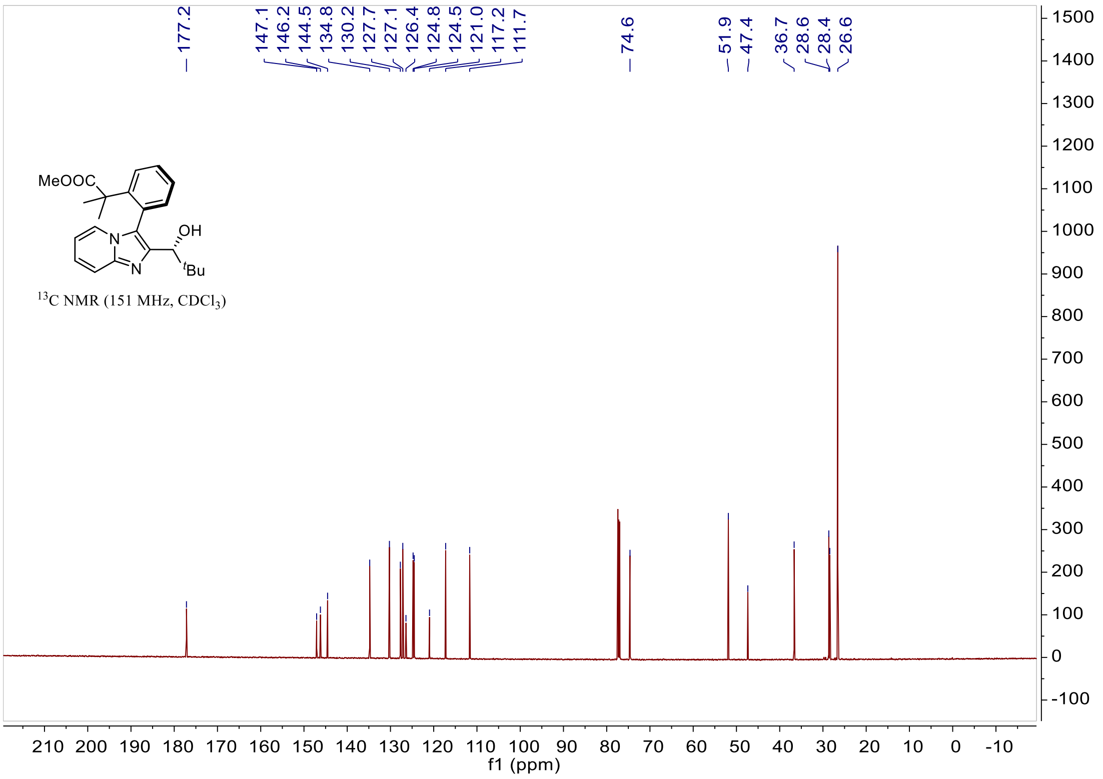
177.2
147.1
146.2
144.5
134.8
130.2
127.7
127.1
126.4
124.8
124.5
121.0
117.2
111.7
74.6
51.9
47.4
36.7
28.6
28.4
26.6
MeOOC
OH
N
N
tBu
13C NMR (151 MHz, CDCl3)
f1 (ppm)

Compound **3u**

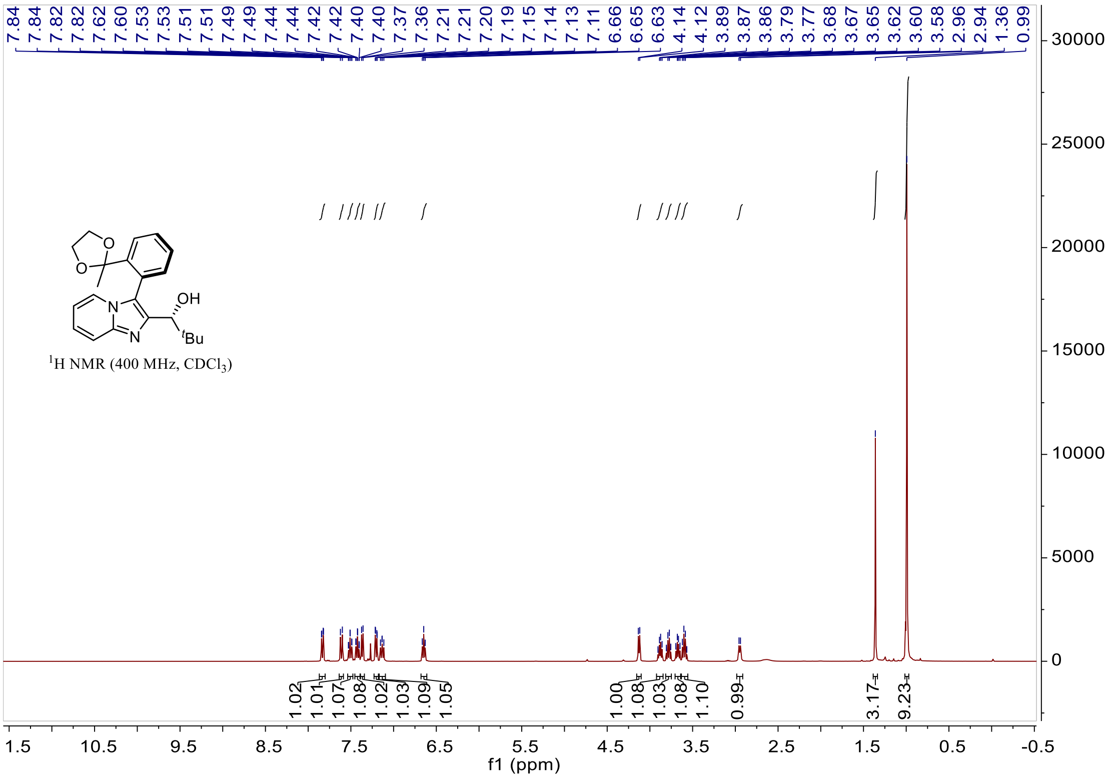

7.84
7.84
7.82
7.82
7.62
7.60
7.53
7.53
7.51
7.51
7.49
7.49
7.44
7.44
7.42
7.42
7.40
7.40
7.37
7.36
7.21
7.21
7.20
7.19
7.15
7.14
7.13
7.11
6.66
6.65
6.63
4.14
4.12
3.89
3.87
3.86
3.79
3.77
3.68
3.67
3.65
3.62
3.60
3.58
2.96
2.94
1.36
0.99
OH
N
N
O
O
tBu
1H NMR (400 MHz, CDCl3)
1.02
1.01
1.07
1.08
1.02
1.03
1.09
1.05
1.00
1.08
1.03
1.08
1.10
0.99
3.17
9.23
f1 (ppm)

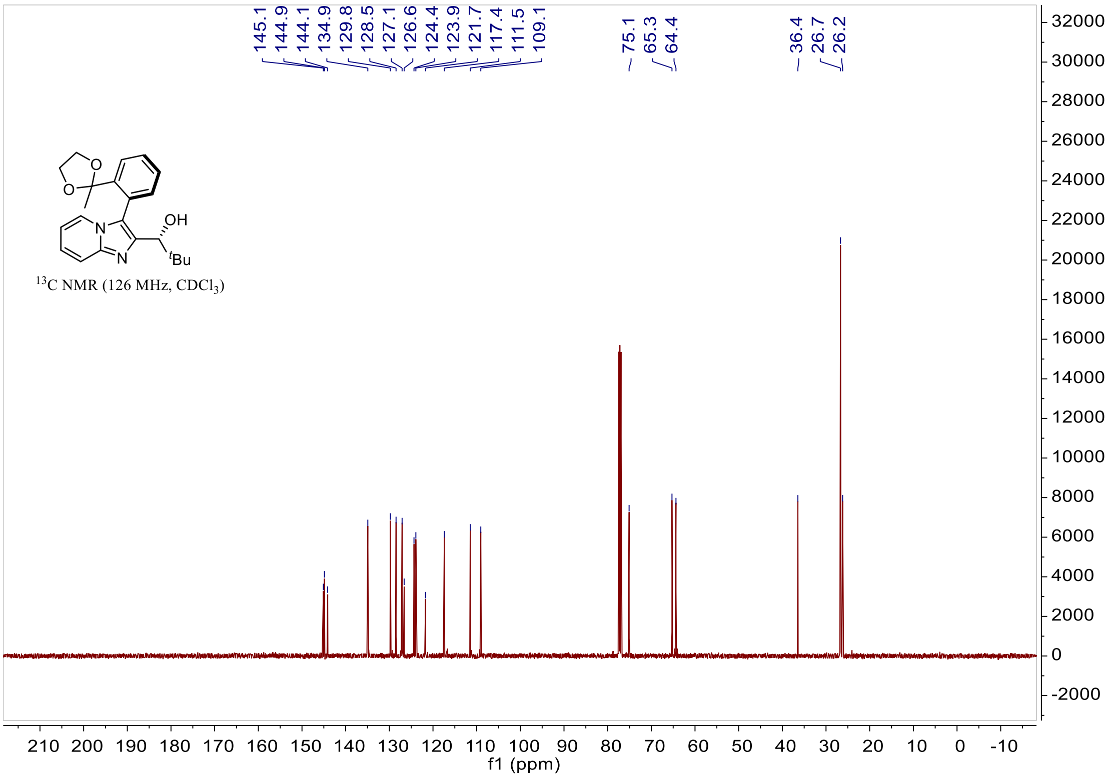

145.1
144.9
144.1
134.9
129.8
128.5
127.1
126.6
124.4
123.9
121.7
117.4
111.5
109.1
75.1
65.3
64.4
36.4
26.7
26.2
OH
N
N
O
O
tBu
13C NMR (126 MHz, CDCl3)
f1 (ppm)

Compound **3v**

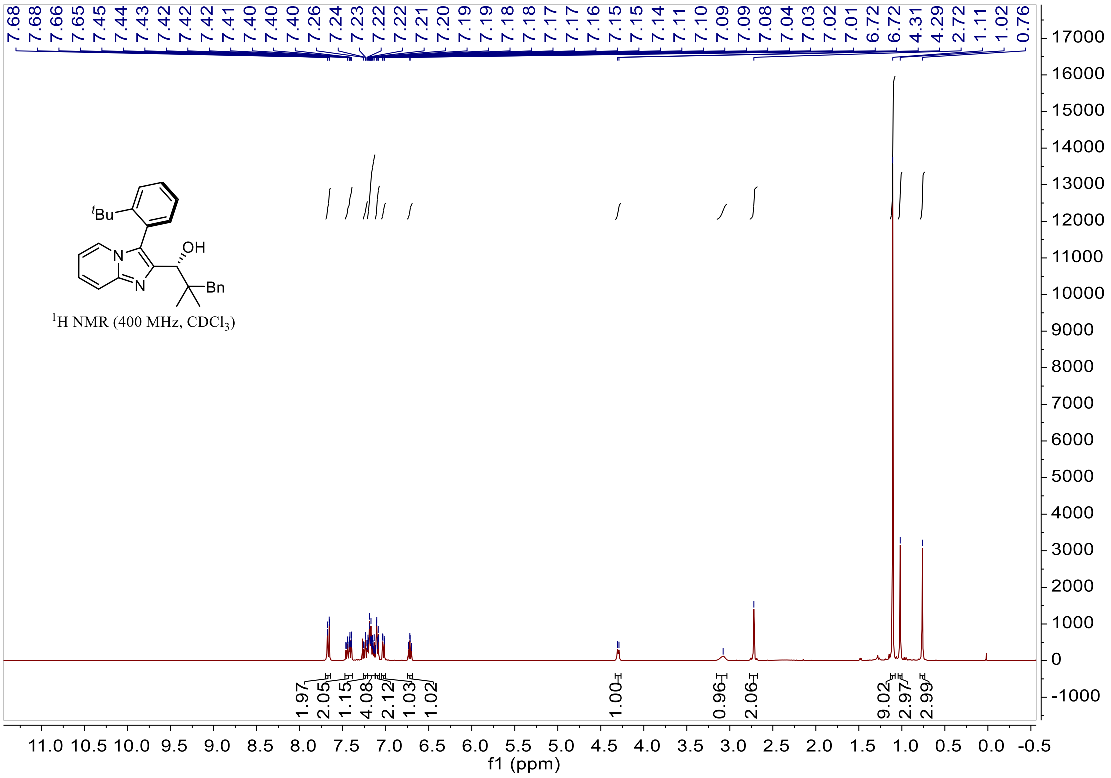
7.68 7.68 7.66 7.65 7.45 7.44 7.43 7.42 7.42 7.42 7.41 7.40 7.40 7.40 7.26 7.24 7.23 7.22 7.22 7.21 7.20 7.19 7.19 7.18 7.18 7.17 7.17 7.16 7.15 7.15 7.14 7.11 7.10 7.09 7.08 7.04 7.03 7.02 7.01 6.72 6.72 4.31 4.29 2.72 1.11 1.02 0.76
tBu
OH
N
N
Bn
1H NMR (400 MHz, CDCl3)
1.97 2.05 1.15 4.08 2.12 1.03 1.02 1.00 0.96 2.06 9.02 2.97 2.99
f1 (ppm)

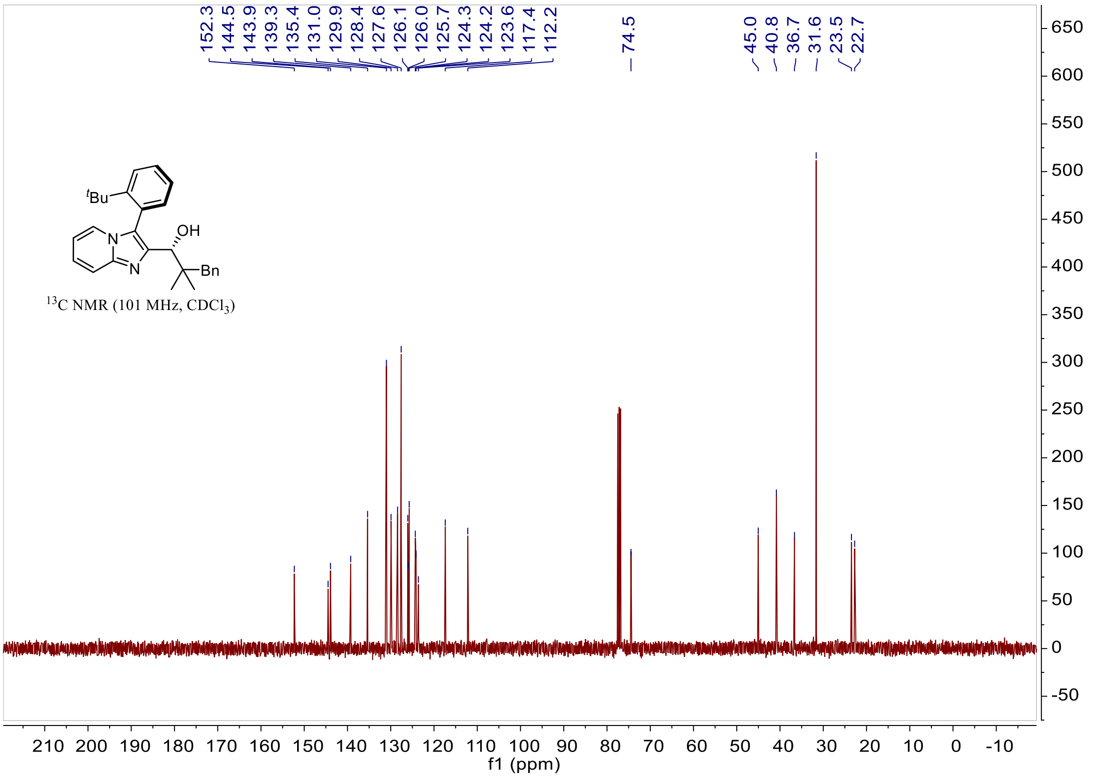
152.3 144.5 143.9 139.3 135.4 131.0 129.9 128.4 127.6 126.1 126.0 125.7 124.3 124.2 123.6 117.4 112.2 74.5 45.0 40.8 36.7 31.6 23.5 22.7
tBu
OH
N
N
Bn
13C NMR (101 MHz, CDCl3)
f1 (ppm)

Compound **3w**

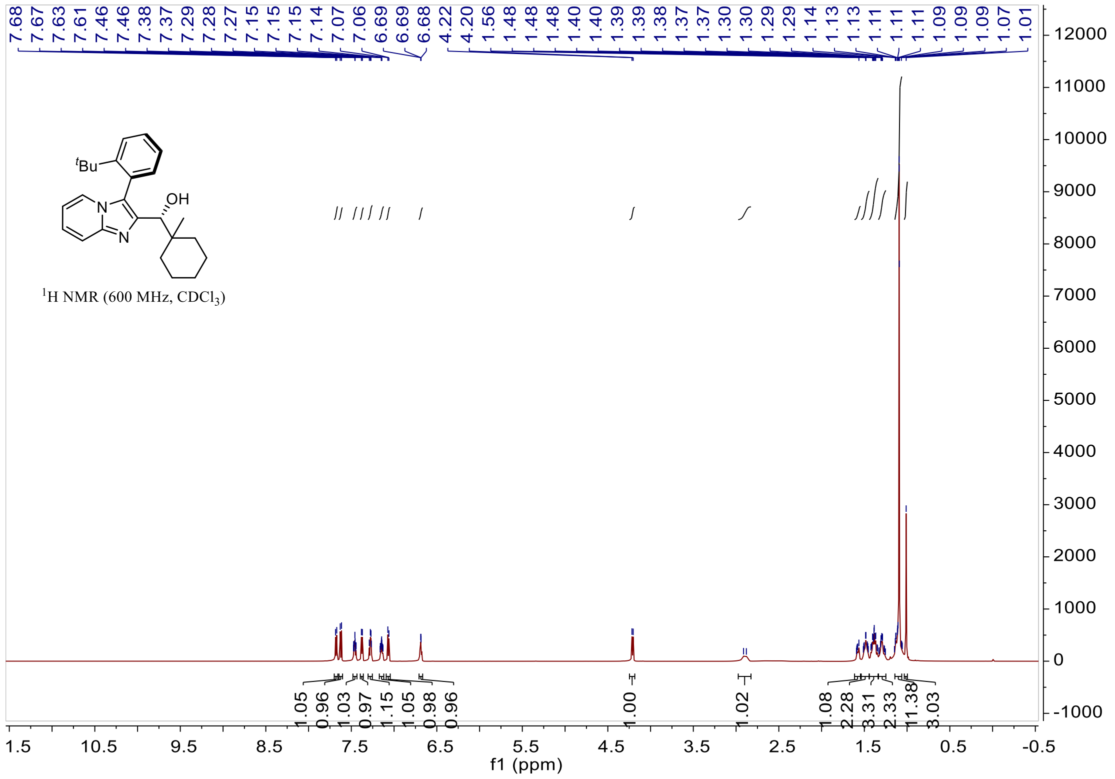
7.68
7.67
7.63
7.61
7.46
7.46
7.38
7.37
7.29
7.28
7.27
7.15
7.15
7.14
7.07
7.06
6.69
6.69
6.68
4.22
4.20
1.56
1.48
1.48
1.40
1.40
1.39
1.39
1.38
1.37
1.37
1.30
1.30
1.29
1.29
1.14
1.13
1.13
1.11
1.11
1.11
1.09
1.09
1.09
1.07
1.01
^tBu
OH
N
N
^1H NMR (600 MHz, $CDCl_3$)
1.05
0.96
1.03
0.97
1.15
1.05
0.98
0.96
1.00
1.02
1.08
2.28
3.31
2.33
11.38
3.03
f1 (ppm)

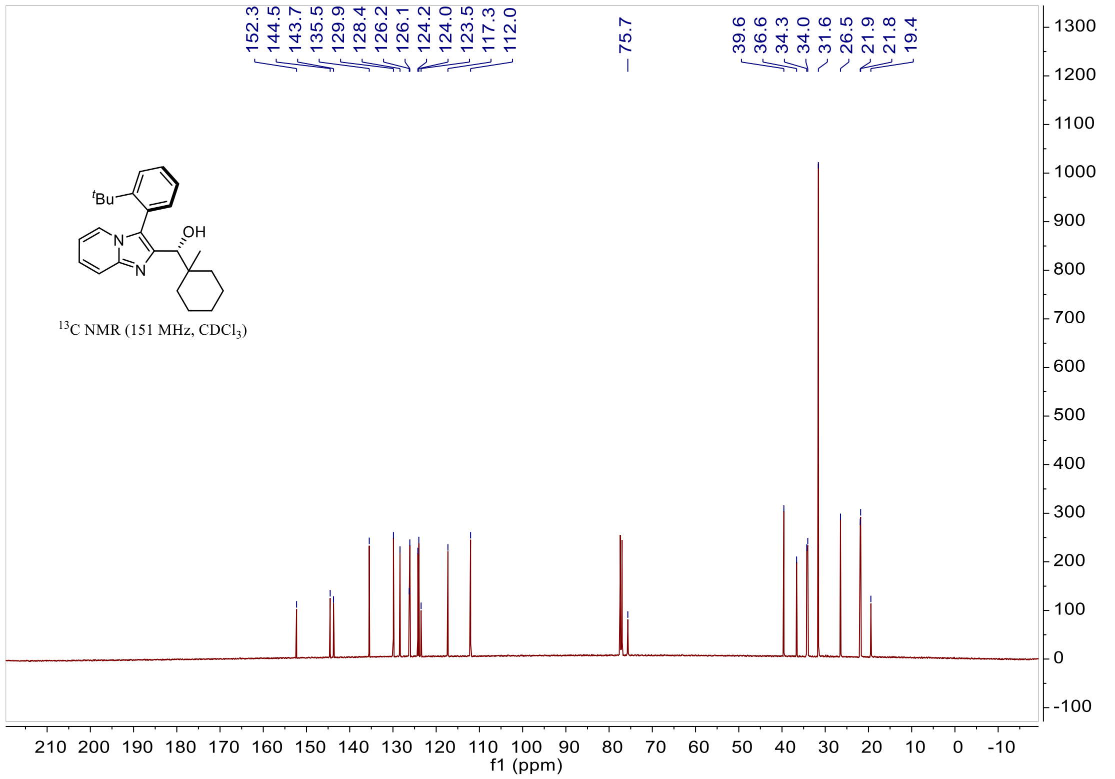
152.3
144.5
143.7
135.5
129.9
128.4
126.2
126.1
124.2
124.0
123.5
117.3
112.0
75.7
39.6
36.6
34.3
34.0
31.6
26.5
21.9
21.8
19.4
^tBu
OH
N
N
^{13}C NMR (151 MHz, $CDCl_3$)
f1 (ppm)

Compound **2a**

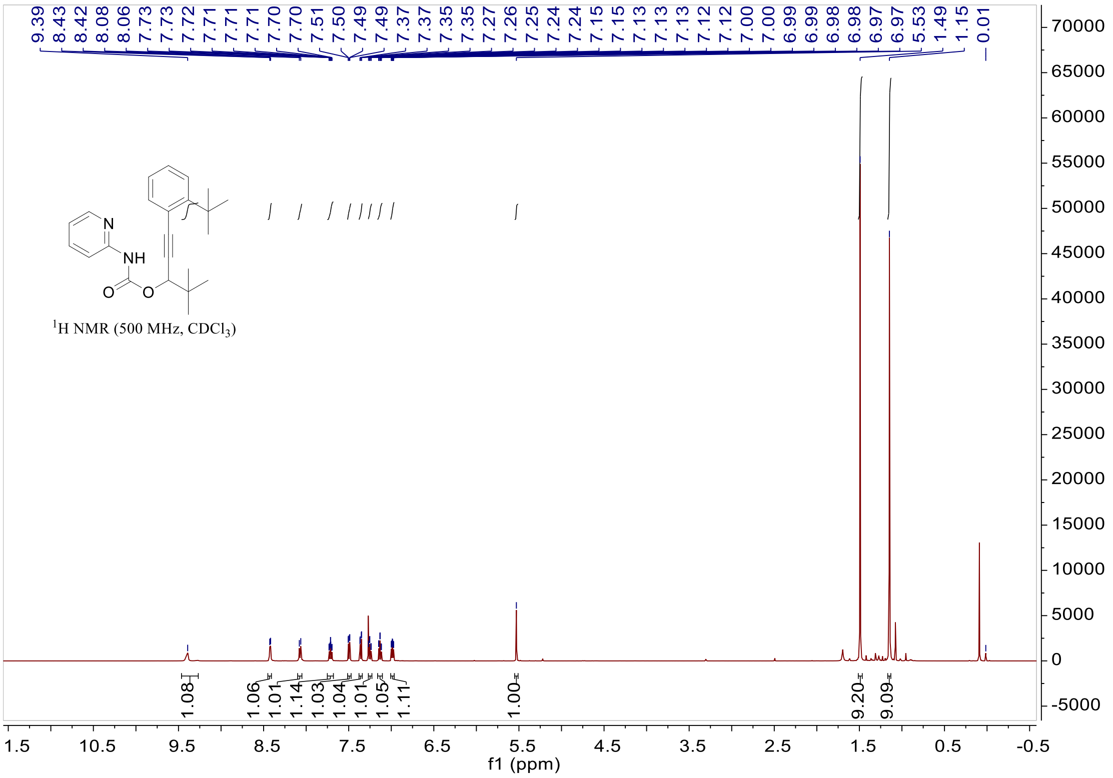

9.39
8.43
8.42
8.08
8.06
7.73
7.73
7.72
7.71
7.71
7.70
7.70
7.51
7.50
7.49
7.49
7.37
7.37
7.35
7.27
7.26
7.25
7.24
7.24
7.15
7.15
7.13
7.13
7.12
7.12
7.00
7.00
6.99
6.99
6.98
6.98
6.97
6.97
5.53
1.49
1.15
0.01
N
NH
O
O
^{1}H NMR (500 MHz, $CDCl_3$)
1.08
1.06
1.01
1.14
1.03
1.04
1.01
1.05
1.11
1.00
9.20
9.09
f1 (ppm)

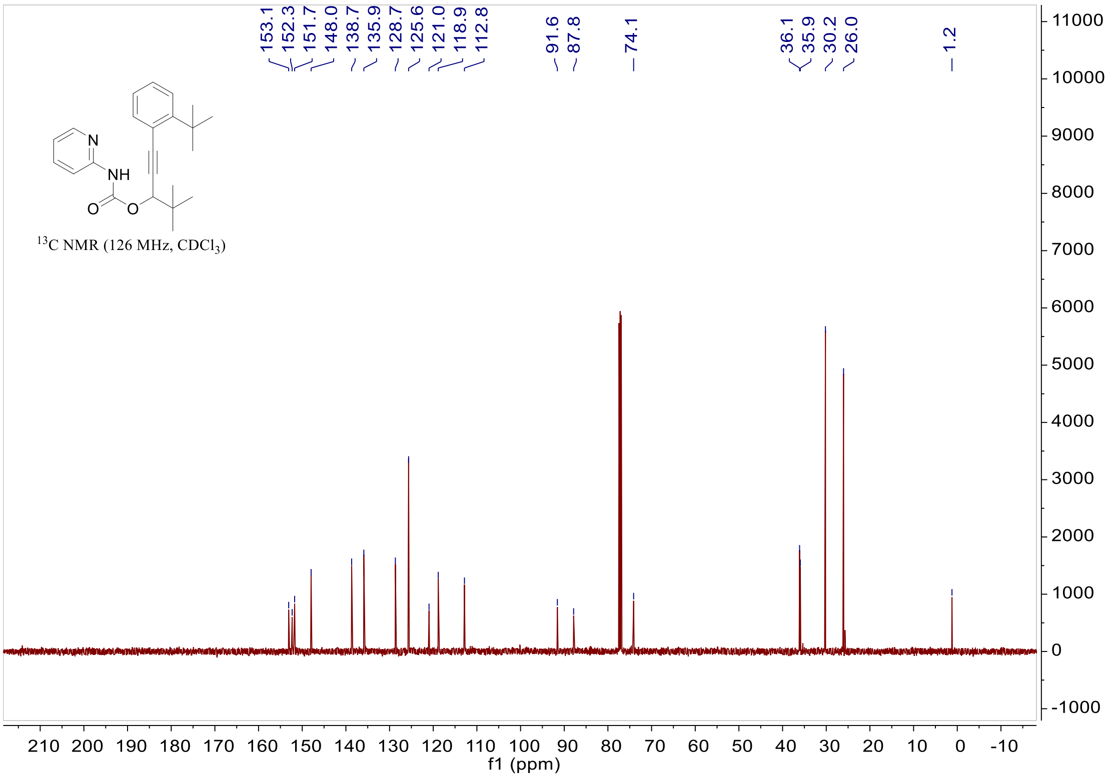

153.1
152.3
151.7
148.0
138.7
135.9
128.7
125.6
121.0
118.9
112.8
91.6
87.8
74.1
36.1
35.9
30.2
26.0
1.2
N
NH
O
O
^{13}C NMR (126 MHz, $CDCl_3$)
f1 (ppm)

Compound **2b**

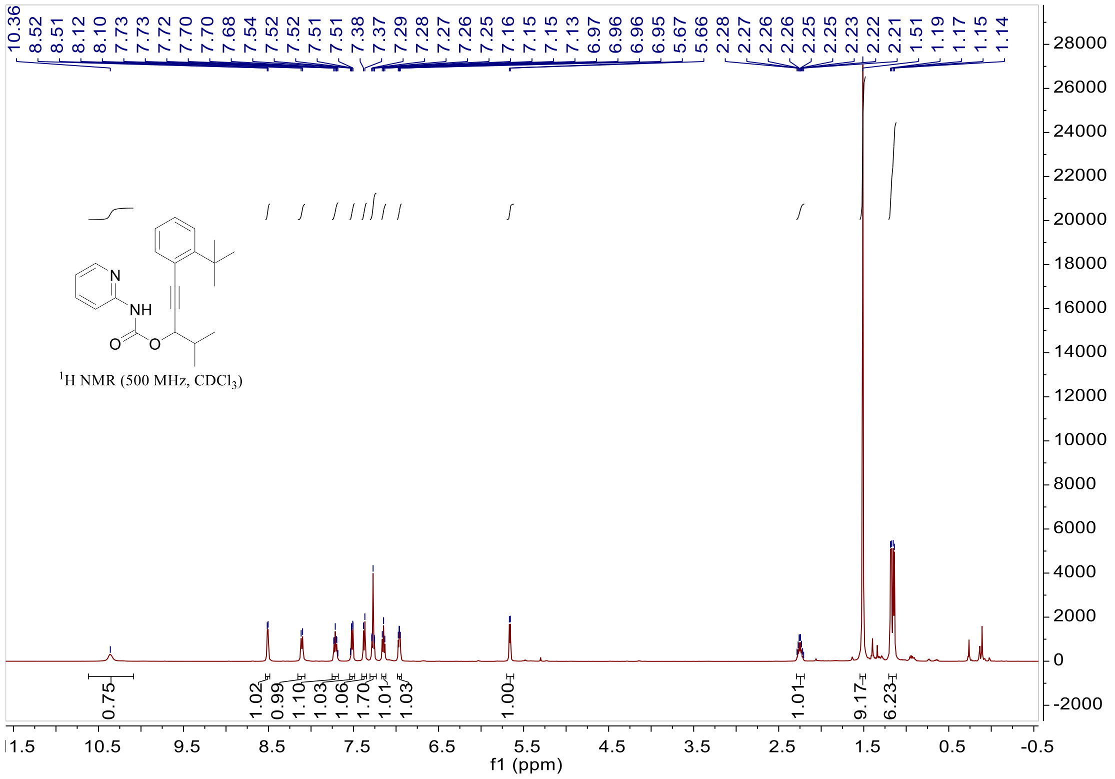

10.36 8.52 8.51 8.12 8.10 7.73 7.73 7.72 7.70 7.70 7.68 7.54 7.52 7.52 7.51 7.51 7.38 7.37 7.29 7.28 7.27 7.26 7.25 7.16 7.15 7.15 7.13 6.97 6.96 6.96 6.95 5.67 5.66 2.28 2.27 2.26 2.26 2.25 2.25 2.23 2.22 2.21 1.51 1.19 1.17 1.15 1.14
N
NH
O
O
1H NMR (500 MHz, CDCl3)
0.75 1.02 0.99 1.10 1.03 1.06 1.70 1.01 1.03 1.00 1.01 9.17 6.23
f1 (ppm)

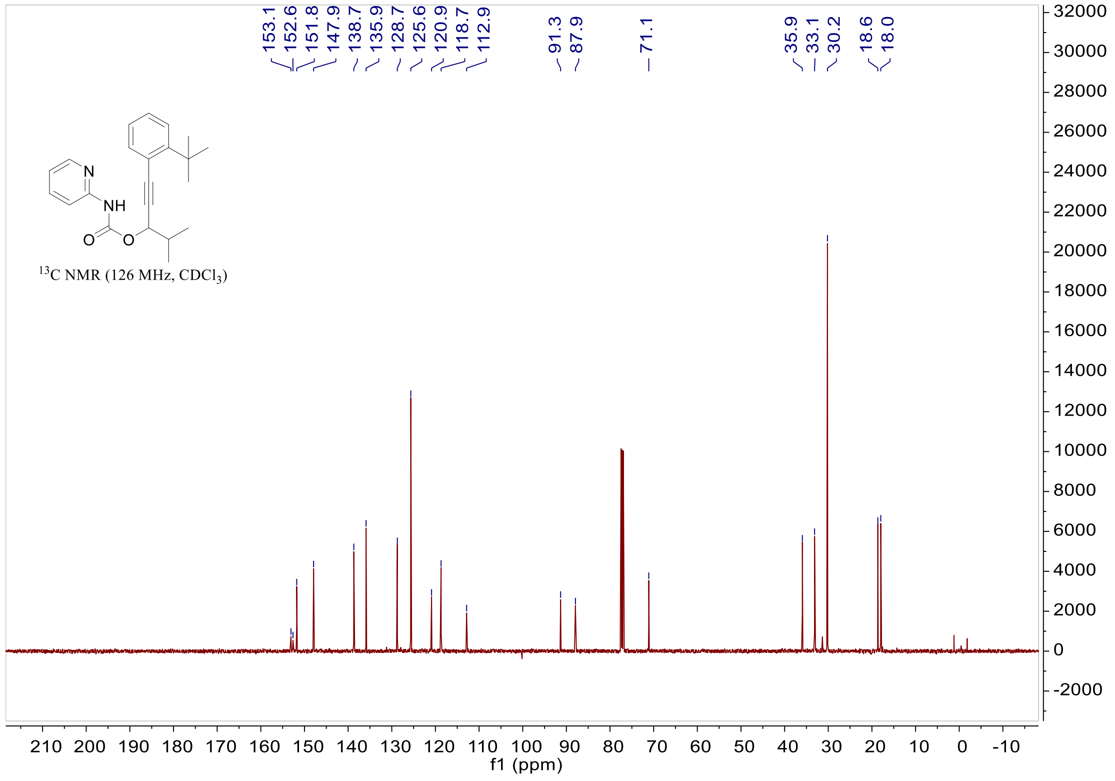

153.1 152.6 151.8 147.9 138.7 135.9 128.7 125.6 120.9 118.7 112.9 91.3 87.9 71.1 35.9 33.1 30.2 18.6 18.0
N
NH
O
O
13C NMR (126 MHz, CDCl3)
f1 (ppm)

Compound **2c**

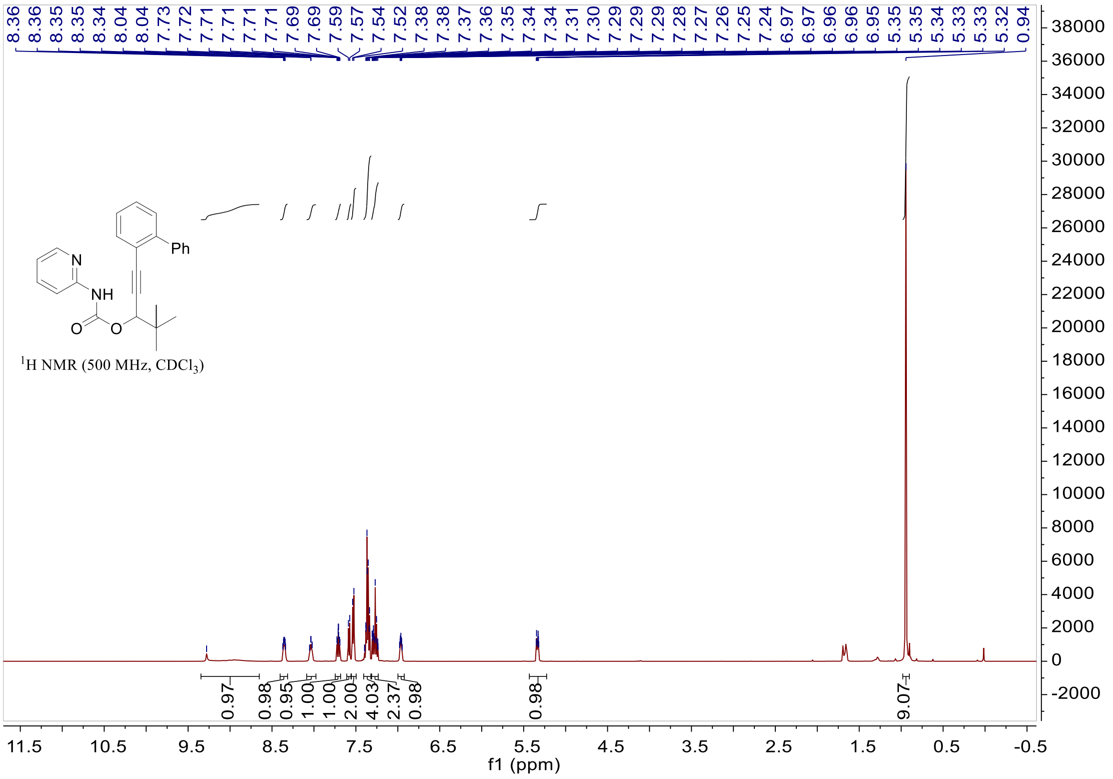

8.36 8.36 8.35 8.34 8.04 8.04 7.73 7.72 7.71 7.71 7.71 7.71 7.69 7.69 7.59 7.57 7.54 7.52 7.38 7.37 7.36 7.35 7.34 7.34 7.31 7.30 7.29 7.29 7.29 7.28 7.27 7.26 7.25 7.24 6.97 6.97 6.96 6.96 6.95 5.35 5.35 5.34 5.33 5.33 5.32 0.94
Ph
N
NH
O
O
¹H NMR (500 MHz, CDCl₃)
0.97 0.98 0.95 1.00 1.00 2.00 4.03 2.37 0.98 0.98 9.07
f1 (ppm)

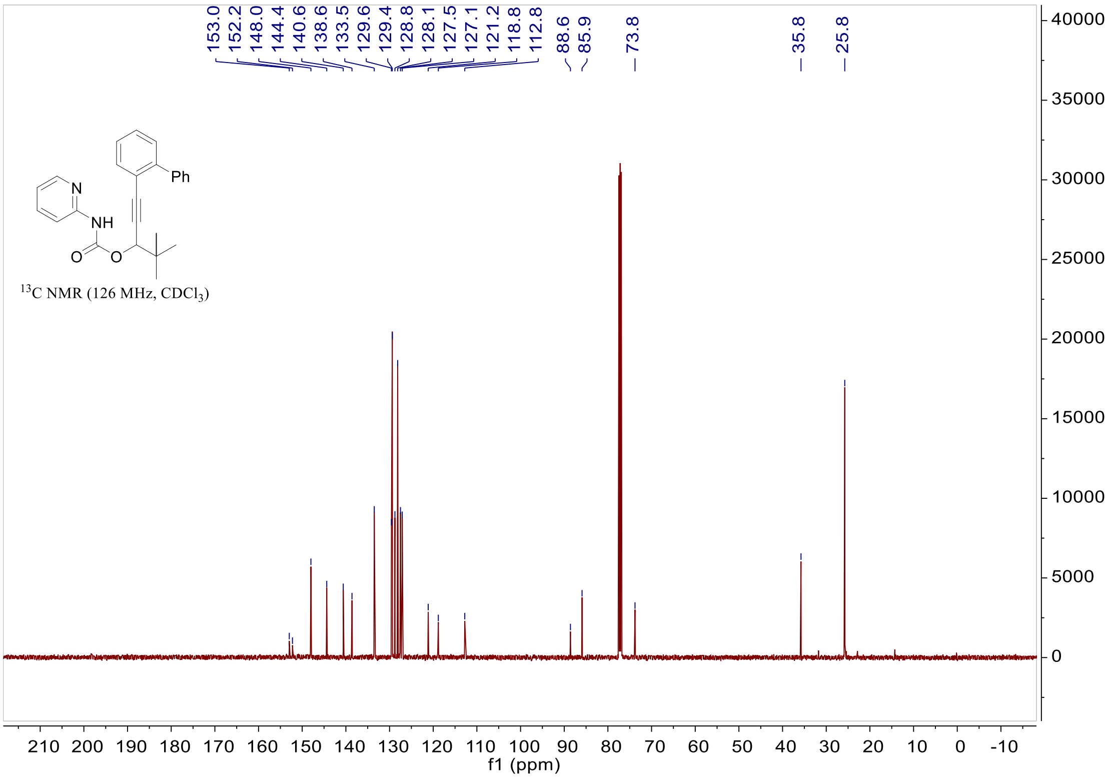

153.0 152.2 148.0 144.4 140.6 138.6 133.5 129.6 129.4 128.8 128.1 127.5 127.1 121.2 118.8 112.8 88.6 85.9 73.8 35.8 25.8
Ph
N
NH
O
O
¹³C NMR (126 MHz, CDCl₃)
f1 (ppm)

Compound **2d**

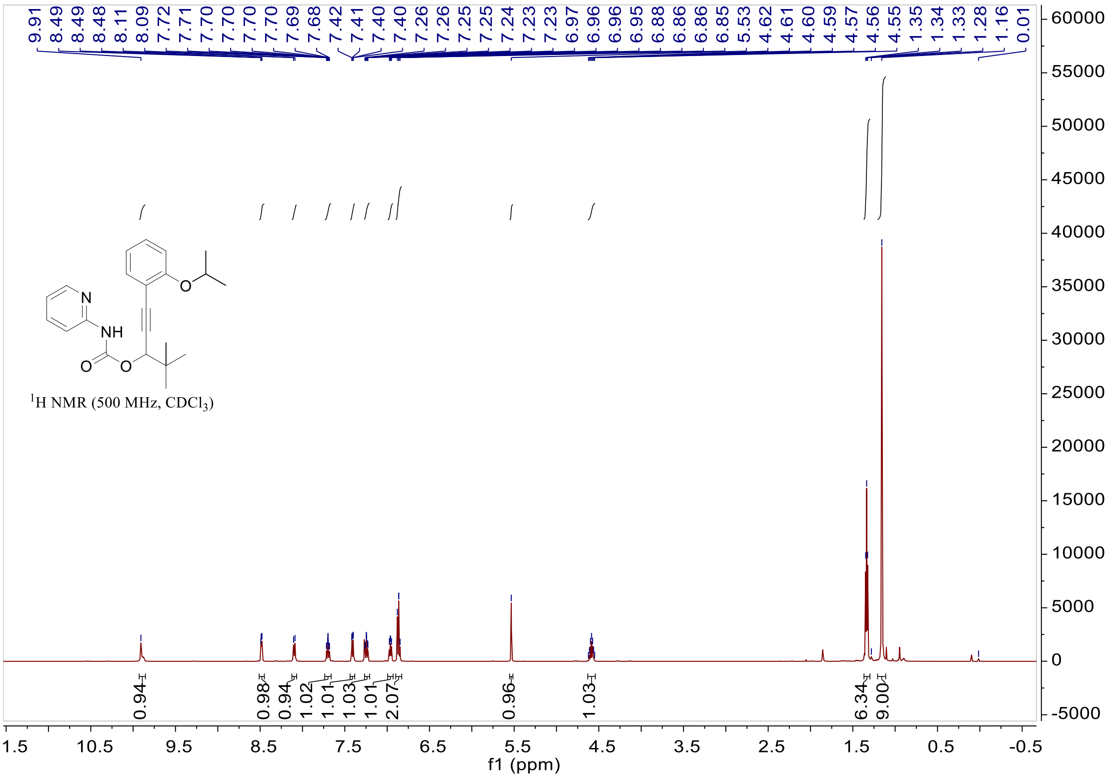
9.91
8.49
8.49
8.48
8.11
8.09
7.72
7.71
7.70
7.70
7.70
7.69
7.68
7.42
7.41
7.40
7.40
7.26
7.26
7.25
7.25
7.24
7.23
7.23
6.97
6.96
6.96
6.95
6.88
6.86
6.86
6.85
5.53
4.62
4.61
4.60
4.59
4.57
4.56
4.55
1.35
1.34
1.33
1.28
1.16
0.01
0.94
0.98
0.94
1.02
1.01
1.03
1.01
2.07
0.96
1.03
6.34
9.00
N
NH
O
O
O
1H NMR (500 MHz, CDCl3)
f1 (ppm)

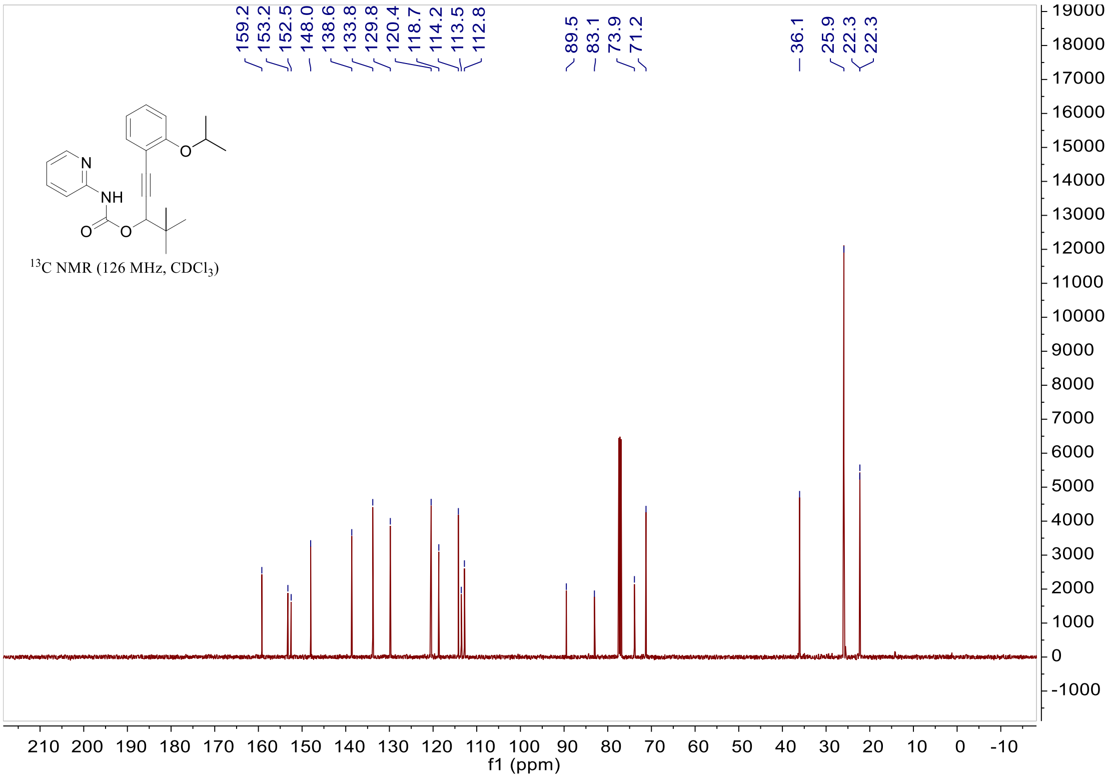
159.2
153.2
152.5
148.0
138.6
133.8
129.8
120.4
118.7
114.2
113.5
112.8
89.5
83.1
73.9
71.2
36.1
25.9
22.3
22.3
N
NH
O
O
O
13C NMR (126 MHz, CDCl3)
f1 (ppm)

Compound **2e**

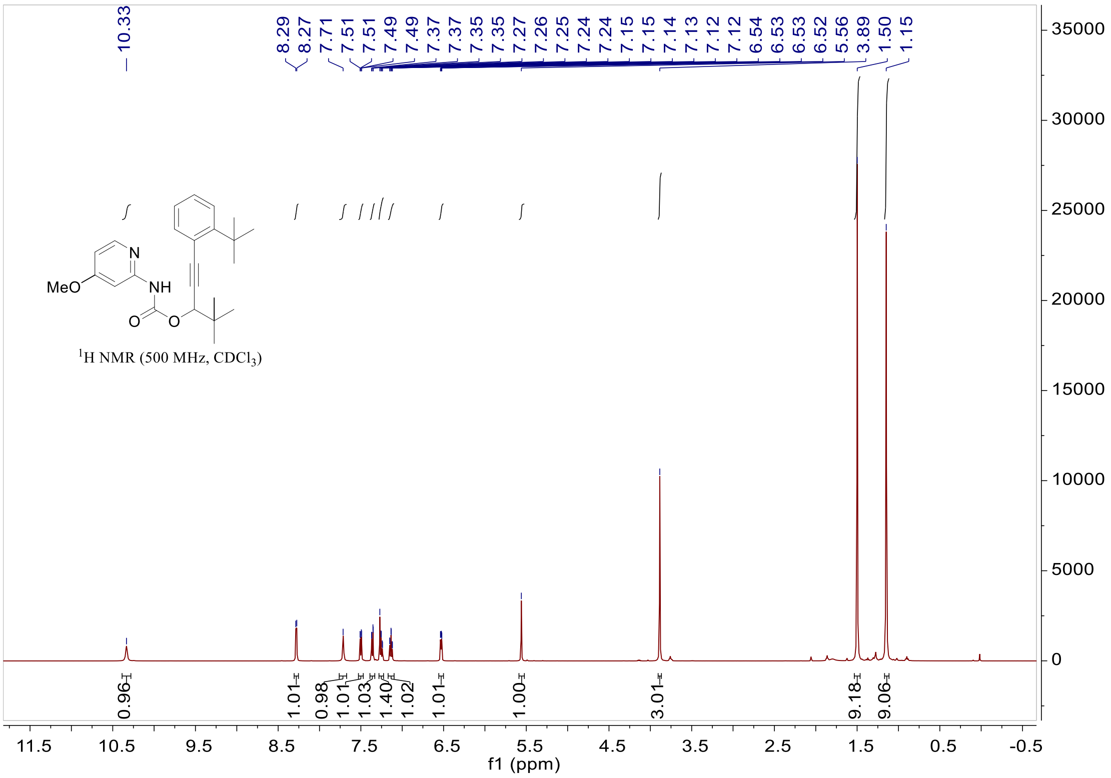
10.33
8.29 8.27 7.71 7.51 7.51 7.49 7.37 7.37 7.35 7.35 7.27 7.26 7.25 7.24 7.24 7.15 7.15 7.14 7.13 7.12 7.12 6.54 6.53 6.52 5.56 3.89 1.50 1.15
MeO
N
NH
O
O
1H NMR (500 MHz, CDCl3)
0.96 1.01 0.98 1.01 1.03 1.40 1.02 1.01 1.00 3.01 9.18 9.06
35000 30000 25000 20000 15000 10000 5000 0
11.5 10.5 9.5 8.5 7.5 6.5 5.5 4.5 3.5 2.5 1.5 0.5 -0.5
f1 (ppm)

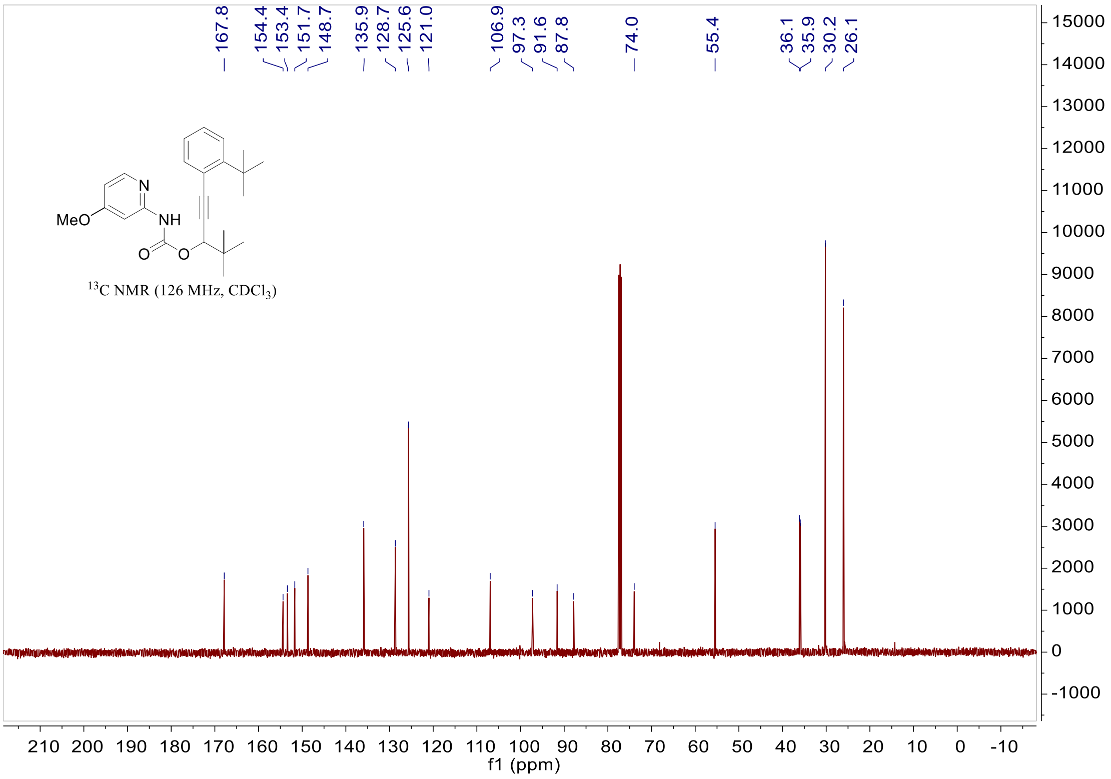
167.8 154.4 153.4 151.7 148.7 135.9 128.7 125.6 121.0 106.9 97.3 91.6 87.8 74.0 55.4 36.1 35.9 30.2 26.1
MeO
N
NH
O
O
13C NMR (126 MHz, CDCl3)
15000 14000 13000 12000 11000 10000 9000 8000 7000 6000 5000 4000 3000 2000 1000 0 -1000
210 200 190 180 170 160 150 140 130 120 110 100 90 80 70 60 50 40 30 20 10 0 -10
f1 (ppm)

Compound **2f**

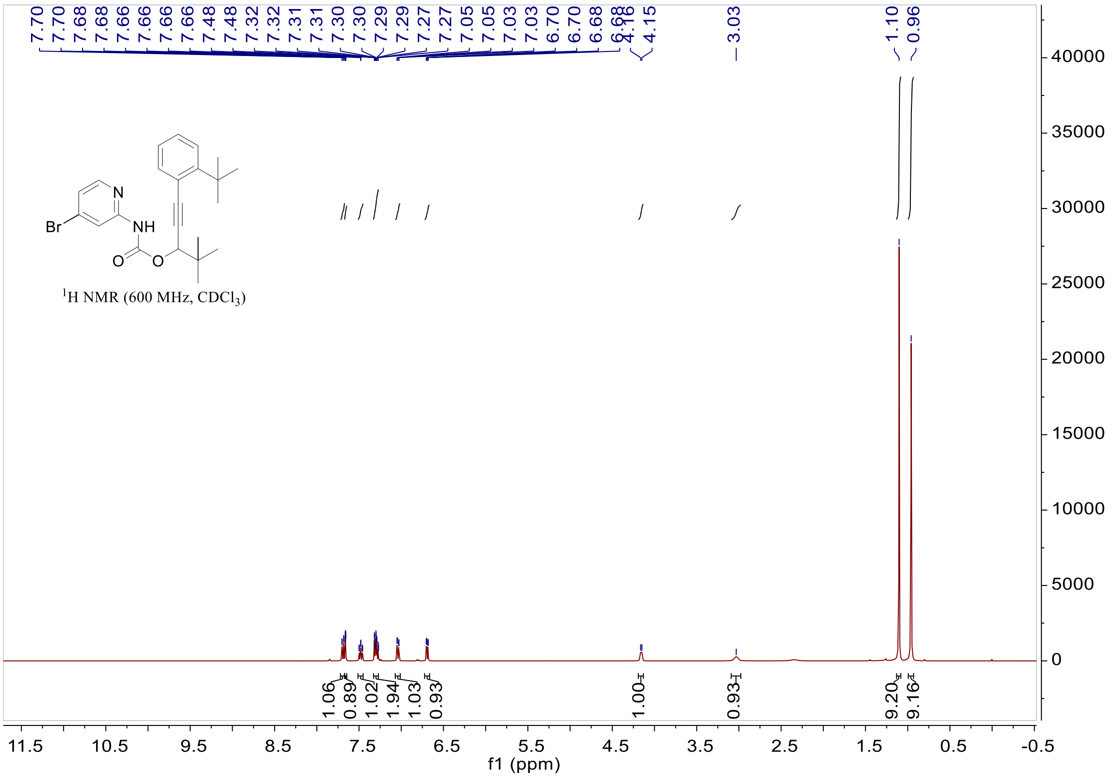

^{1}H NMR (600 MHz, $CDCl_3$)

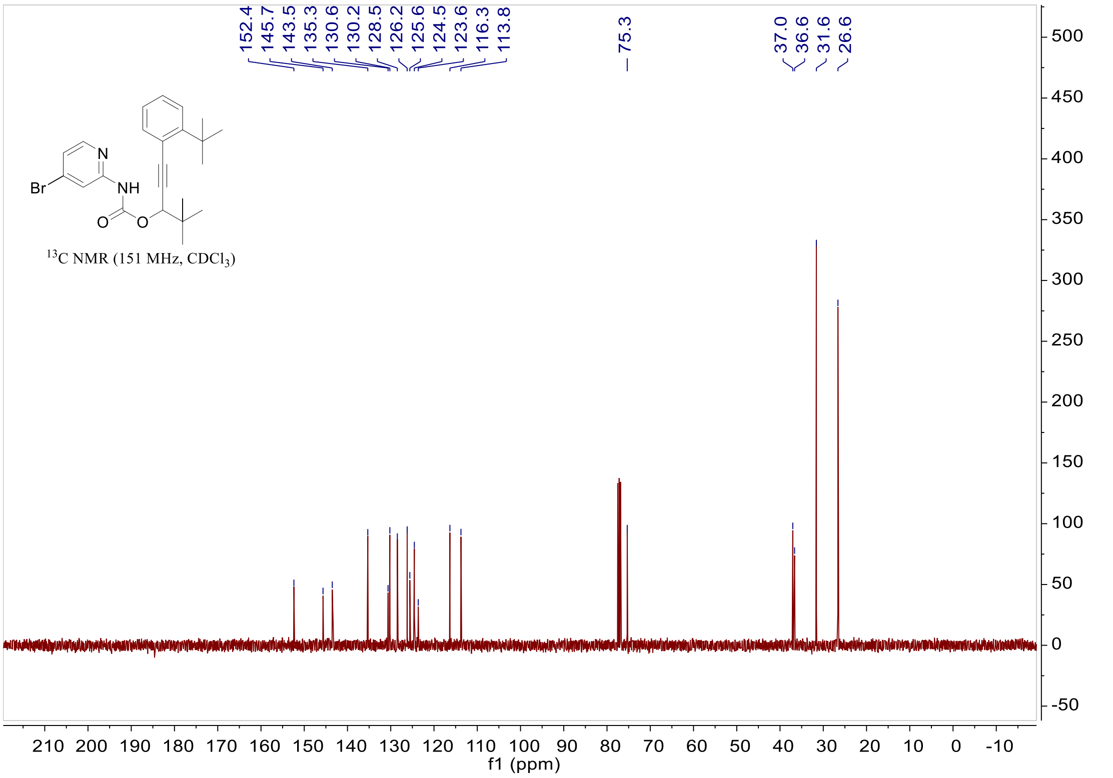

^{13}C NMR (151 MHz, $CDCl_3$)

Compound **2g**

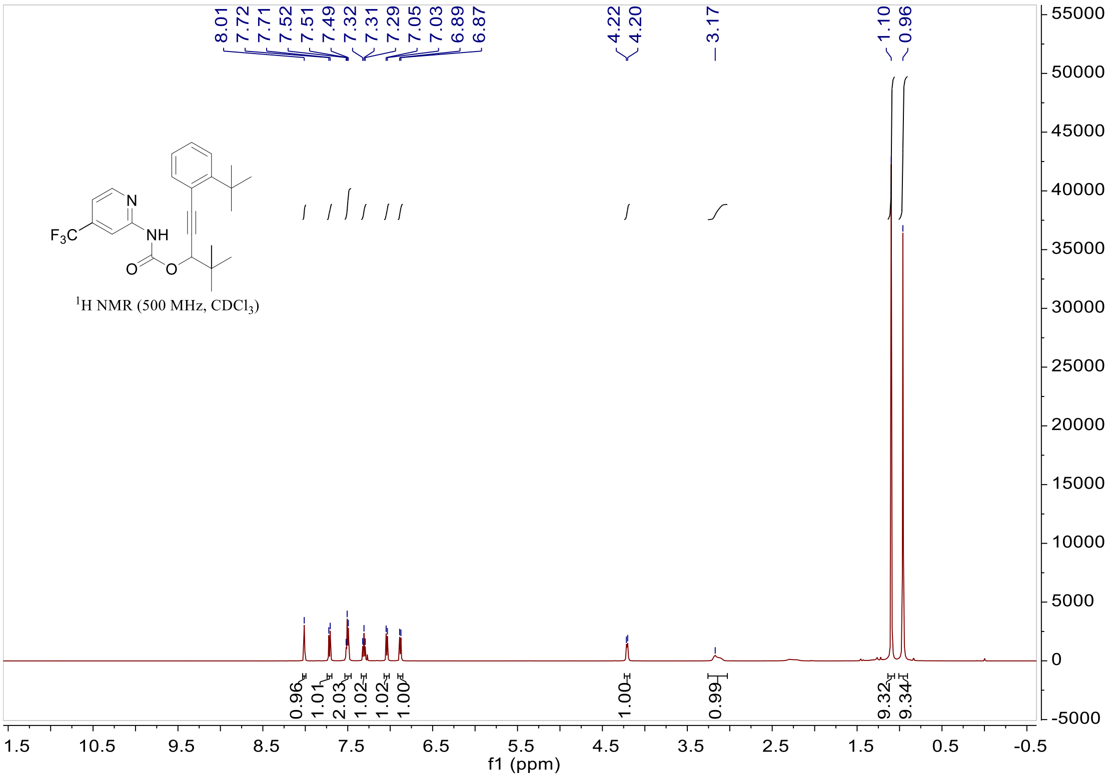
8.01
7.72
7.71
7.52
7.51
7.49
7.32
7.31
7.29
7.05
7.03
6.89
6.87
4.22
4.20
3.17
1.10
0.96
F3C
N
NH
O
O
1H NMR (500 MHz, CDCl3)
0.96
1.01
2.03
1.02
1.02
1.00
1.00
0.99
9.32
9.34
55000
50000
45000
40000
35000
30000
25000
20000
15000
10000
5000
0
-5000
1.5
10.5
9.5
8.5
7.5
6.5
5.5
4.5
3.5
2.5
1.5
0.5
-0.5
f1 (ppm)

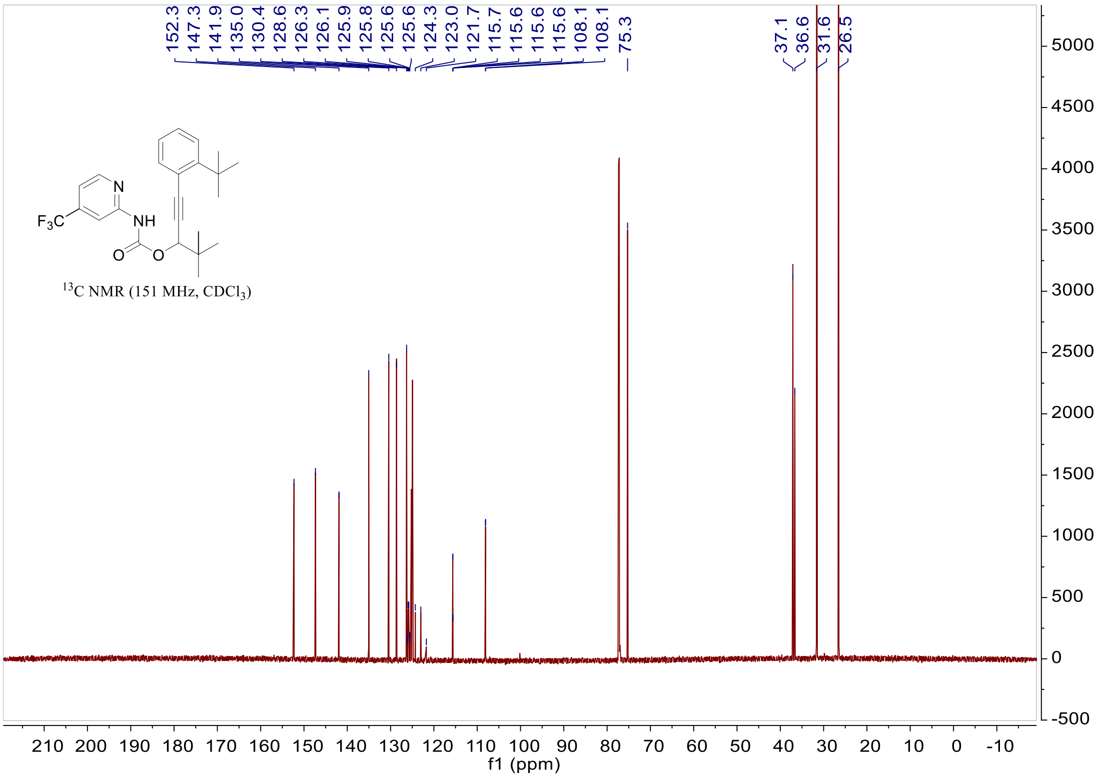
152.3
147.3
141.9
135.0
130.4
128.6
126.3
126.1
125.9
125.8
125.6
125.6
124.3
123.0
121.7
115.7
115.6
115.6
115.6
108.1
108.1
75.3
37.1
36.6
31.6
26.5
F3C
N
NH
O
O
13C NMR (151 MHz, CDCl3)
5000
4500
4000
3500
3000
2500
2000
1500
1000
500
0
-500
210
200
190
180
170
160
150
140
130
120
110
100
90
80
70
60
50
40
30
20
10
0
-10
f1 (ppm)

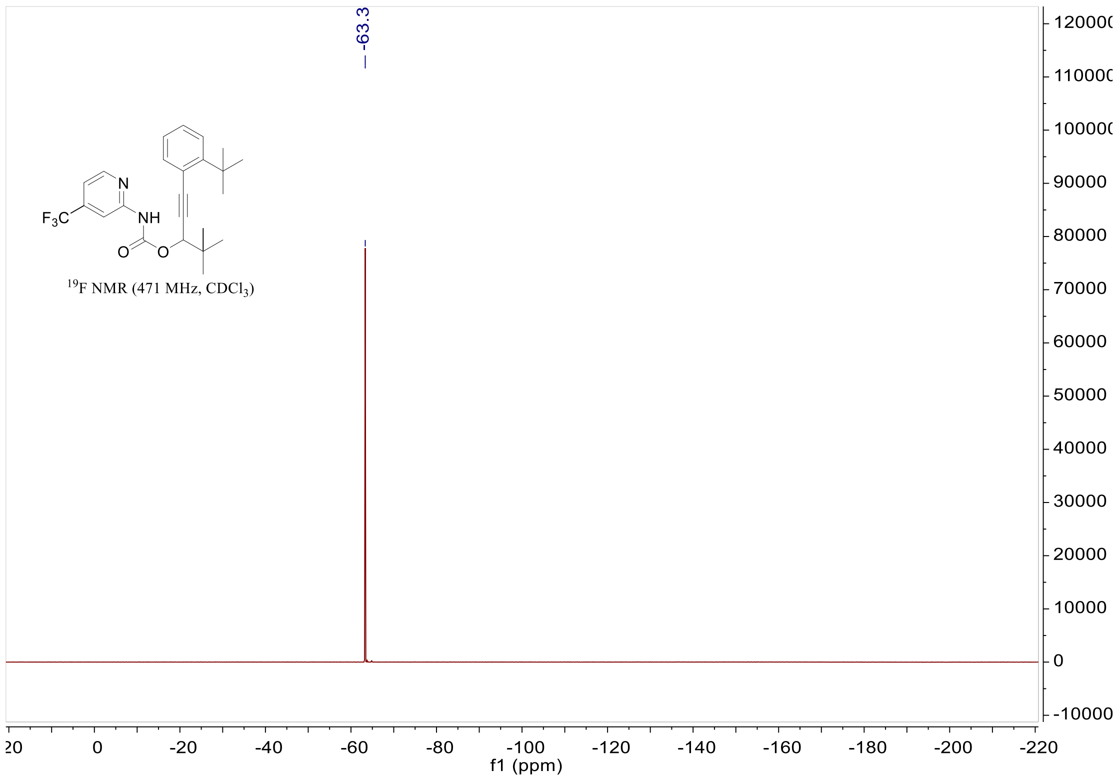
-63.3
F3C
N
NH
O
O
19F NMR (471 MHz, CDCl3)
120000
110000
100000
90000
80000
70000
60000
50000
40000
30000
20000
10000
0
-10000
20
0
-20
-40
-60
-80
-100
-120
-140
-160
-180
-200
-220
f1 (ppm)

Compound **2h**

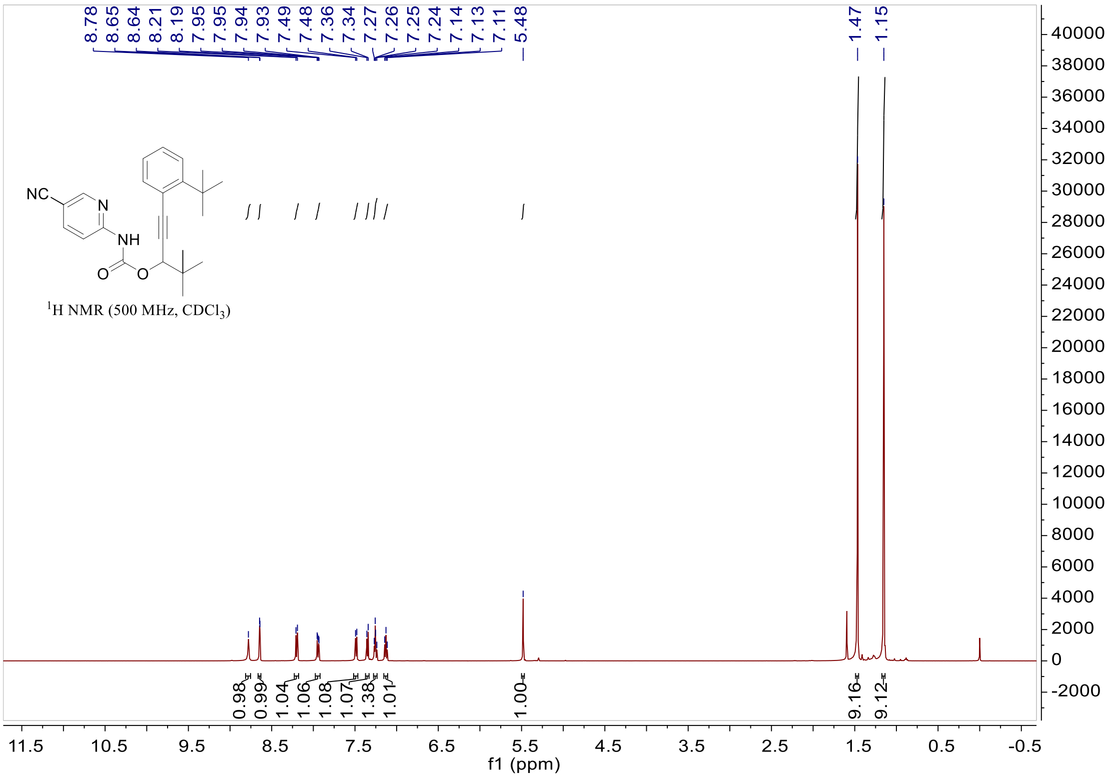
8.78
8.65
8.64
8.21
8.19
7.95
7.95
7.94
7.93
7.49
7.48
7.36
7.34
7.27
7.26
7.25
7.24
7.14
7.13
7.11
5.48
1.47
1.15
NC
N
NH
O
O
¹H NMR (500 MHz, CDCl₃)
0.98
0.99
1.04
1.06
1.08
1.07
1.38
1.01
1.00
9.16
9.12
f1 (ppm)

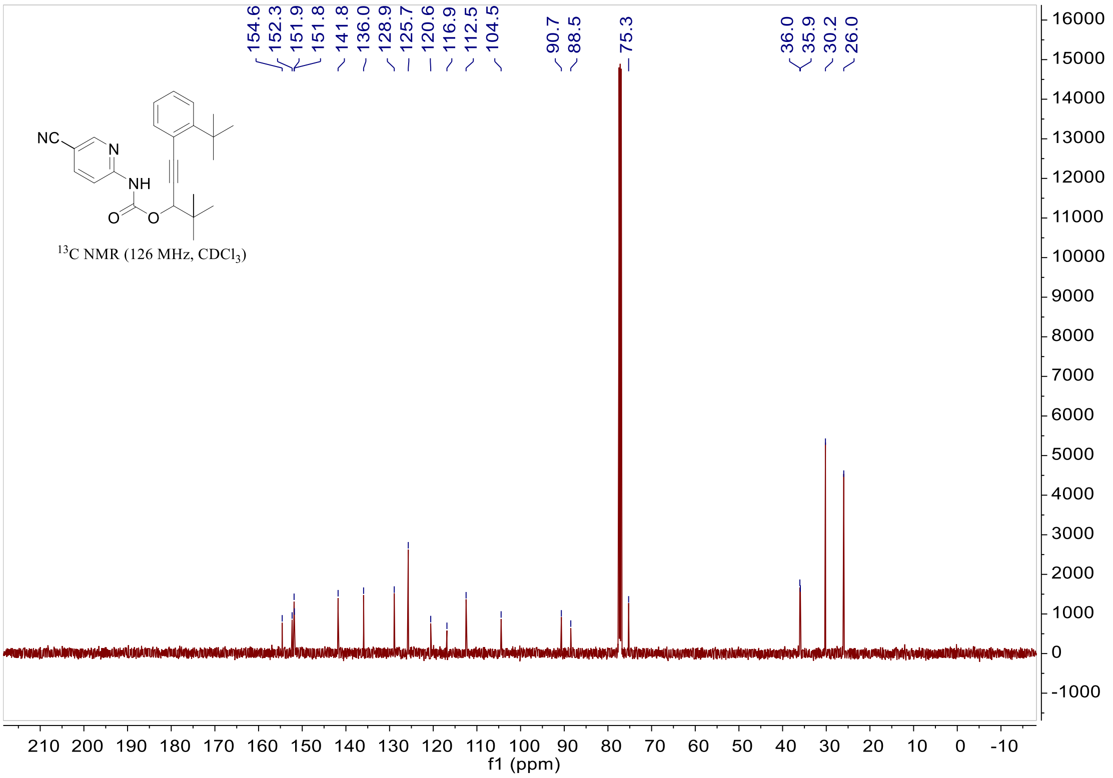
154.6
152.3
151.9
151.8
141.8
136.0
128.9
125.7
120.6
116.9
112.5
104.5
90.7
88.5
75.3
36.0
35.9
30.2
26.0
NC
N
NH
O
O
¹³C NMR (126 MHz, CDCl₃)
f1 (ppm)

Compound **2i**

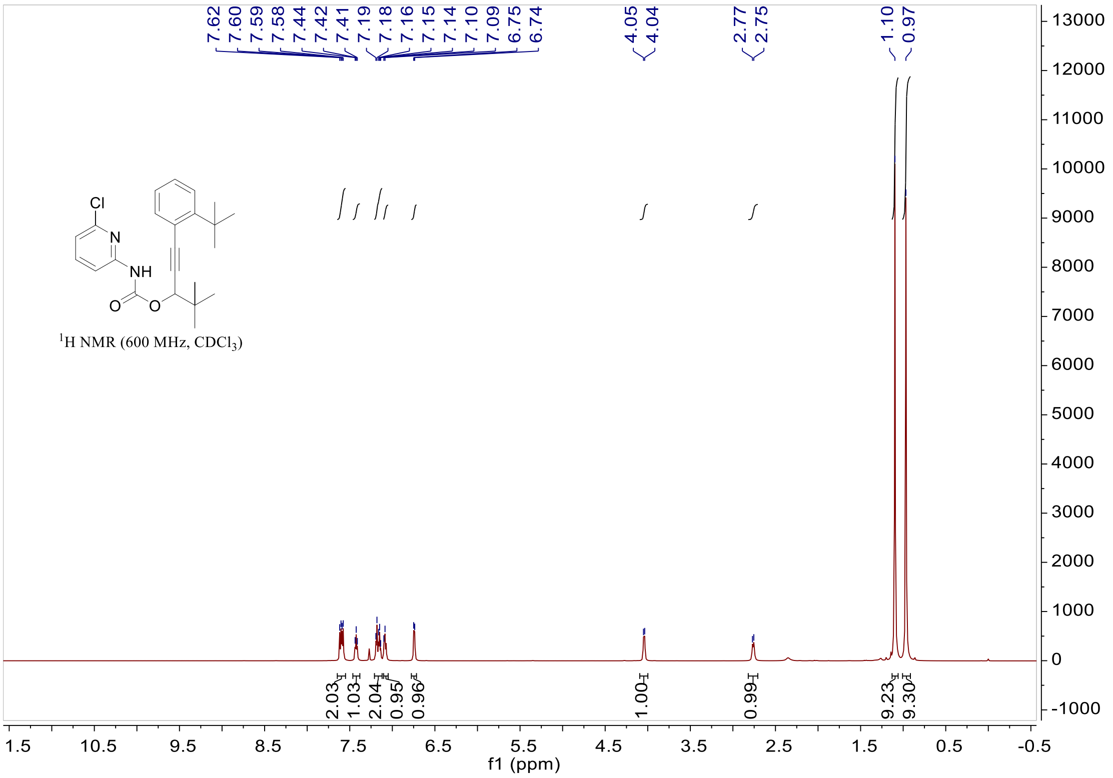

7.62
7.60
7.59
7.58
7.44
7.42
7.41
7.19
7.18
7.16
7.15
7.14
7.10
7.09
6.75
6.74
4.05
4.04
2.77
2.75
1.10
0.97
Cl
N
NH
O
O
^{1}H NMR (600 MHz, CDCl$_3$)
2.03
1.03
2.04
0.95
0.96
1.00
0.99
9.23
9.30
f1 (ppm)

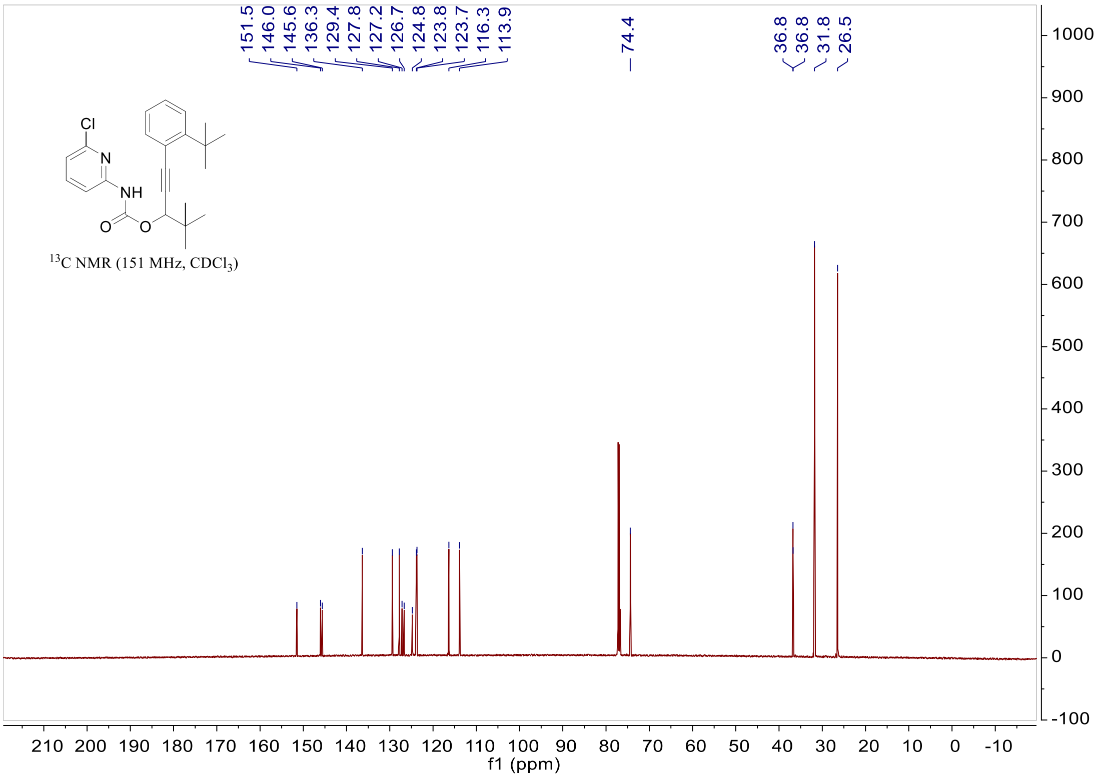

151.5
146.0
145.6
136.3
129.4
127.8
127.2
126.7
124.8
123.8
123.7
116.3
113.9
74.4
36.8
36.8
31.8
26.5
Cl
N
NH
O
O
^{13}C NMR (151 MHz, CDCl$_3$)
f1 (ppm)

Compound **2j**

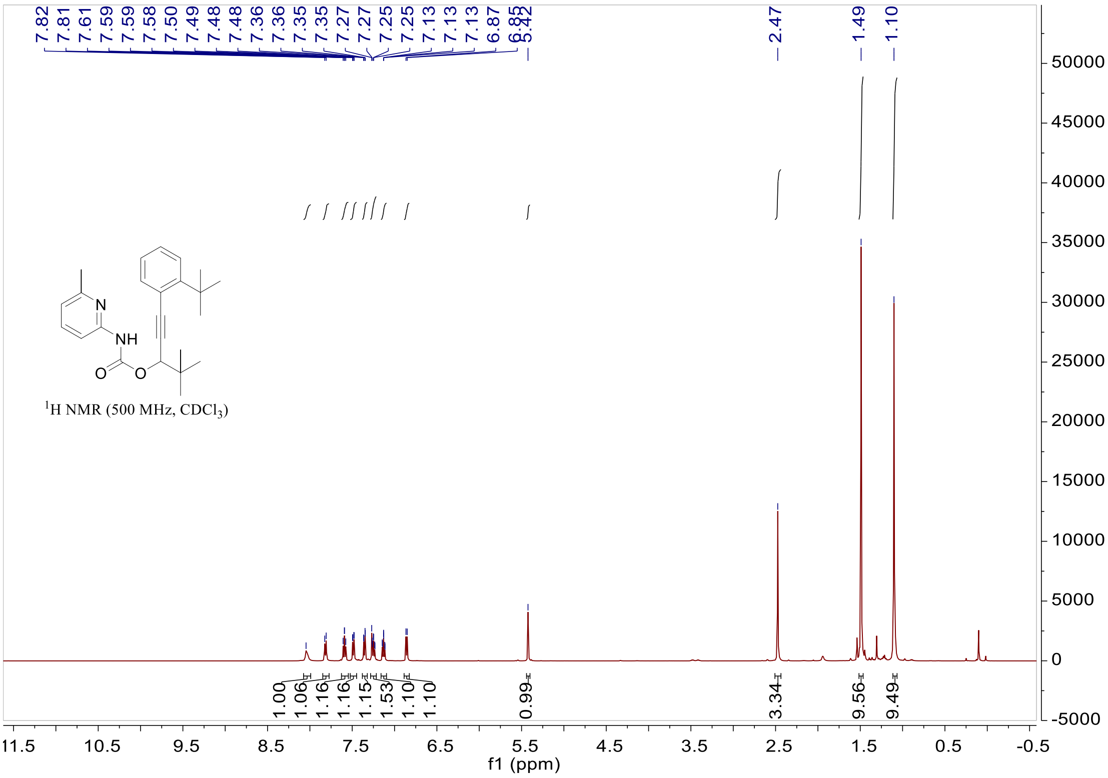
7.82
7.81
7.61
7.59
7.59
7.58
7.50
7.49
7.48
7.48
7.36
7.35
7.35
7.27
7.27
7.25
7.25
7.13
7.13
7.13
6.87
6.85
5.42
2.47
1.49
1.10
N
NH
O
O
^{1}H NMR (500 MHz, $CDCl_3$)
1.00
1.06
1.16
1.16
1.15
1.53
1.10
1.10
0.99
3.34
9.56
9.49
f1 (ppm)

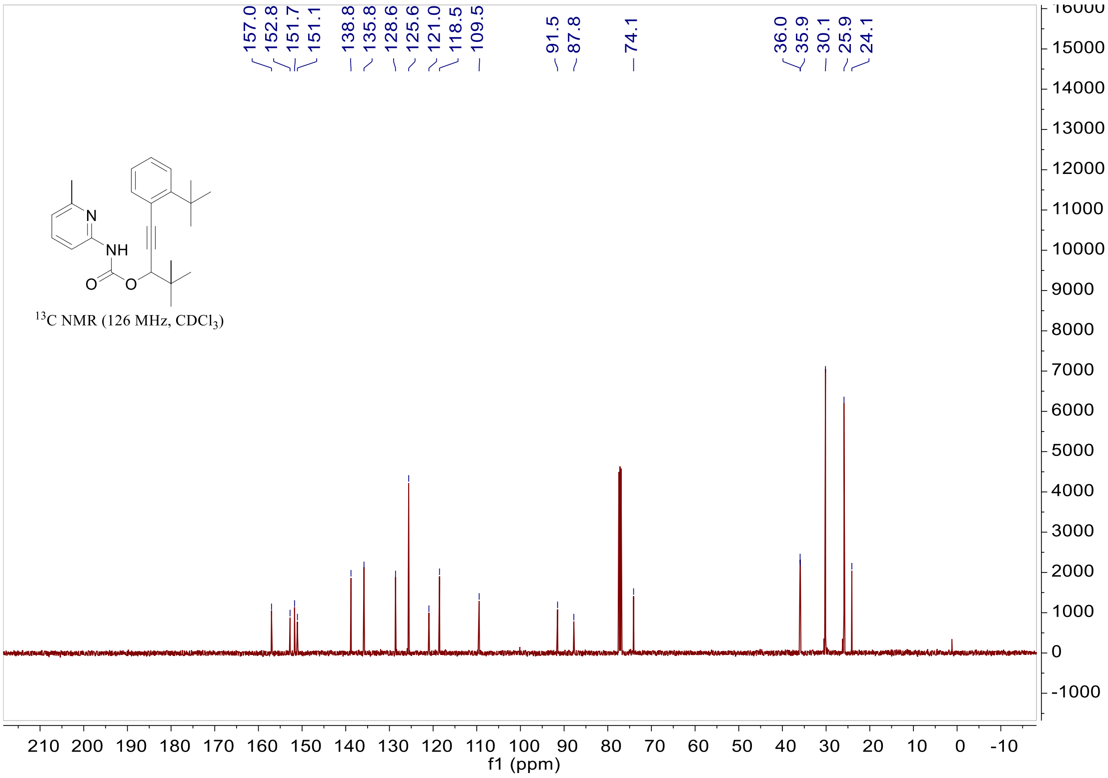
157.0
152.8
151.7
151.1
138.8
135.8
128.6
125.6
121.0
118.5
109.5
91.5
87.8
74.1
36.0
35.9
30.1
25.9
24.1
N
NH
O
O
^{13}C NMR (126 MHz, $CDCl_3$)
f1 (ppm)

Compound **2k**

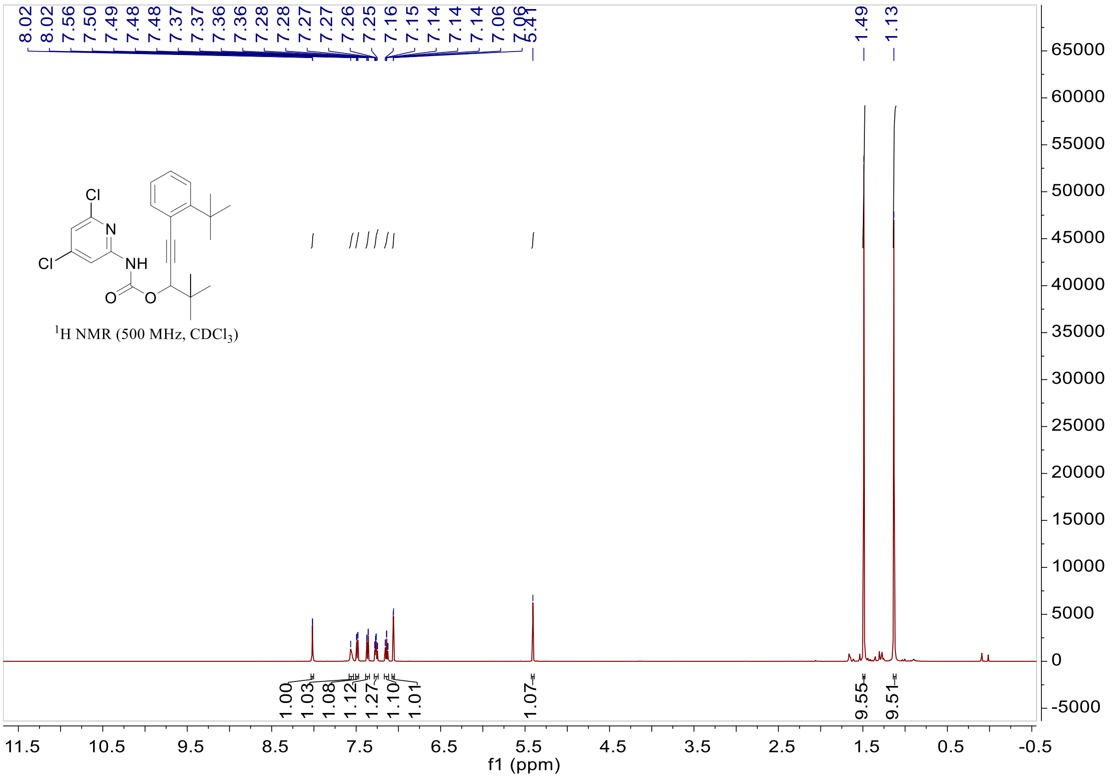

8.02
8.02
7.56
7.50
7.49
7.48
7.48
7.37
7.37
7.36
7.36
7.28
7.28
7.27
7.27
7.26
7.25
7.16
7.15
7.14
7.14
7.14
7.14
7.06
7.06
5.41
1.49
1.13
Cl
N
Cl
NH
O
O
^{1}H NMR (500 MHz, CDCl$_3$)
1.00
1.03
1.08
1.12
1.27
1.10
1.01
1.07
9.55
9.51
f1 (ppm)

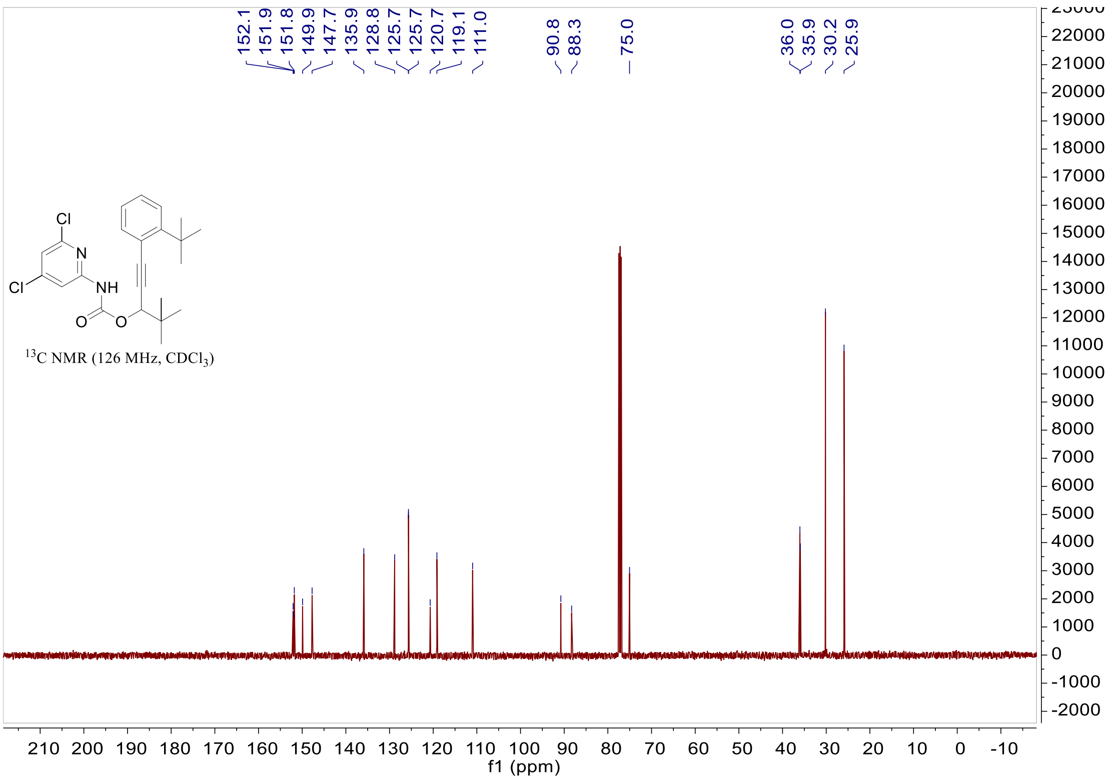

152.1
151.9
151.8
149.9
147.7
135.9
128.8
125.7
125.7
120.7
119.1
111.0
90.8
88.3
75.0
36.0
35.9
30.2
25.9
Cl
N
Cl
NH
O
O
^{13}C NMR (126 MHz, CDCl$_3$)
f1 (ppm)

Compound **2l**

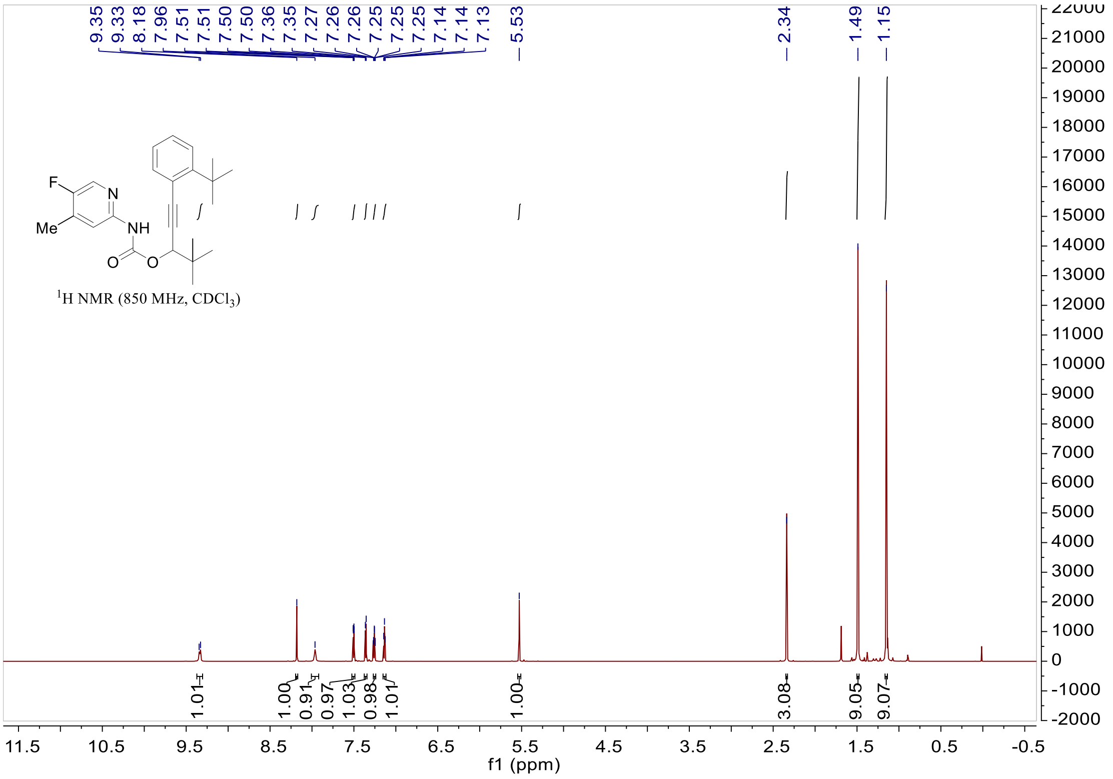

9.35
9.33
8.18
7.96
7.51
7.51
7.50
7.36
7.35
7.27
7.26
7.26
7.25
7.25
7.14
7.14
7.13
5.53
2.34
1.49
1.15
F
N
Me
NH
O
O
^{1}H NMR (850 MHz, $CDCl_3$)
1.01
1.00
0.91
0.97
1.03
0.98
1.01
1.00
3.08
9.05
9.07
f1 (ppm)

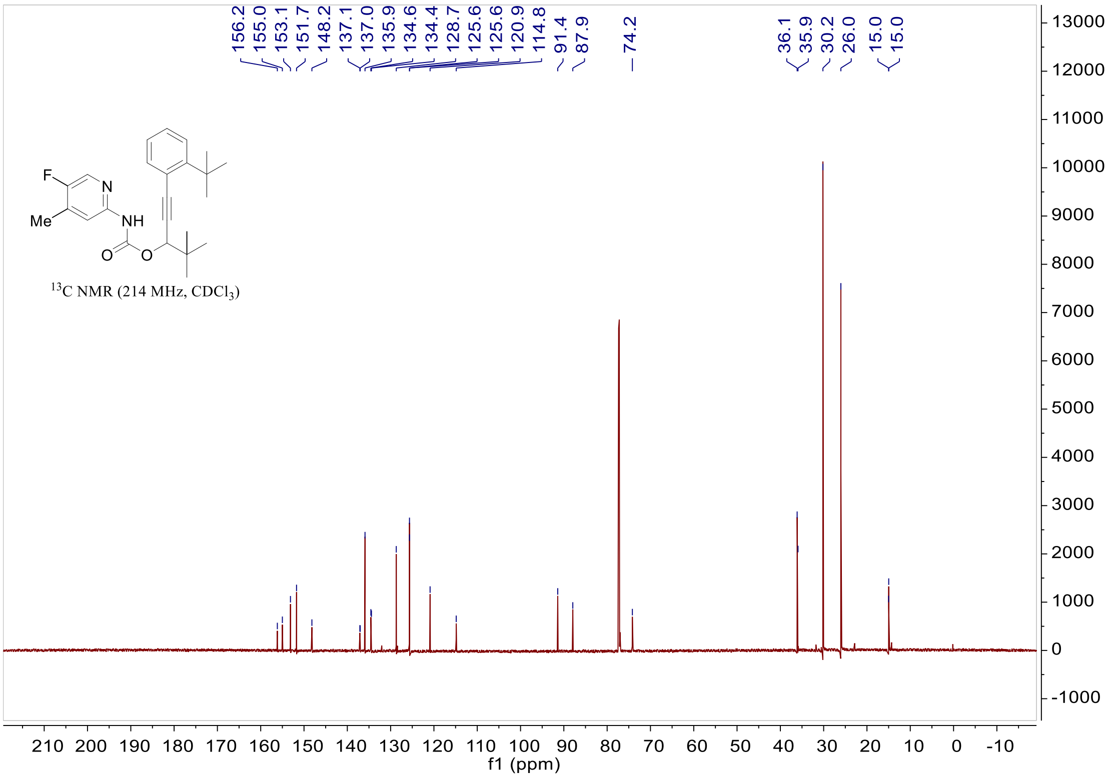

156.2
155.0
153.1
151.7
148.2
137.1
137.0
135.9
134.6
134.4
128.7
125.6
125.6
120.9
114.8
91.4
87.9
74.2
36.1
35.9
30.2
26.0
15.0
15.0
F
N
Me
NH
O
O
^{13}C NMR (214 MHz, $CDCl_3$)
f1 (ppm)

Compound **2m**

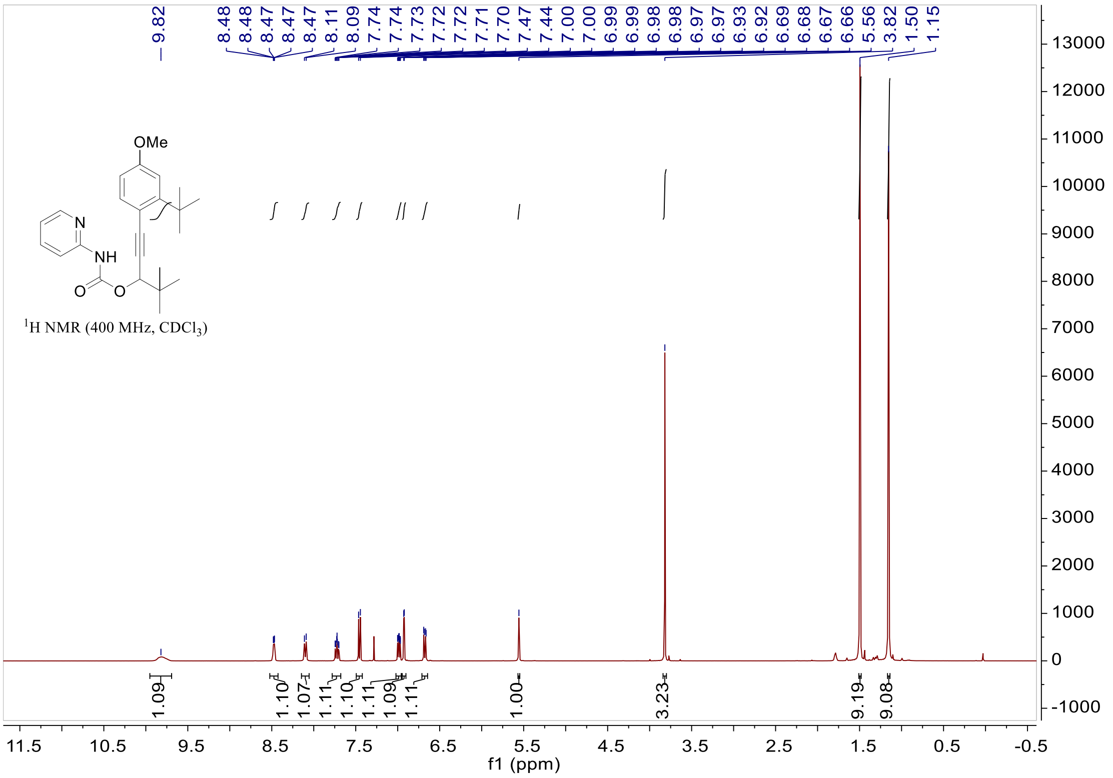
9.82
8.48
8.48
8.47
8.47
8.47
8.11
8.09
7.74
7.74
7.73
7.72
7.72
7.71
7.70
7.47
7.44
7.00
7.00
6.99
6.99
6.98
6.98
6.97
6.97
6.93
6.92
6.69
6.68
6.67
6.66
5.56
3.82
1.50
1.15
OMe
N
NH
O
O
^{1}H NMR (400 MHz, CDCl$_3$)
1.09
1.10
1.07
1.11
1.10
1.11
1.09
1.11
1.00
3.23
9.19
9.08
f1 (ppm)

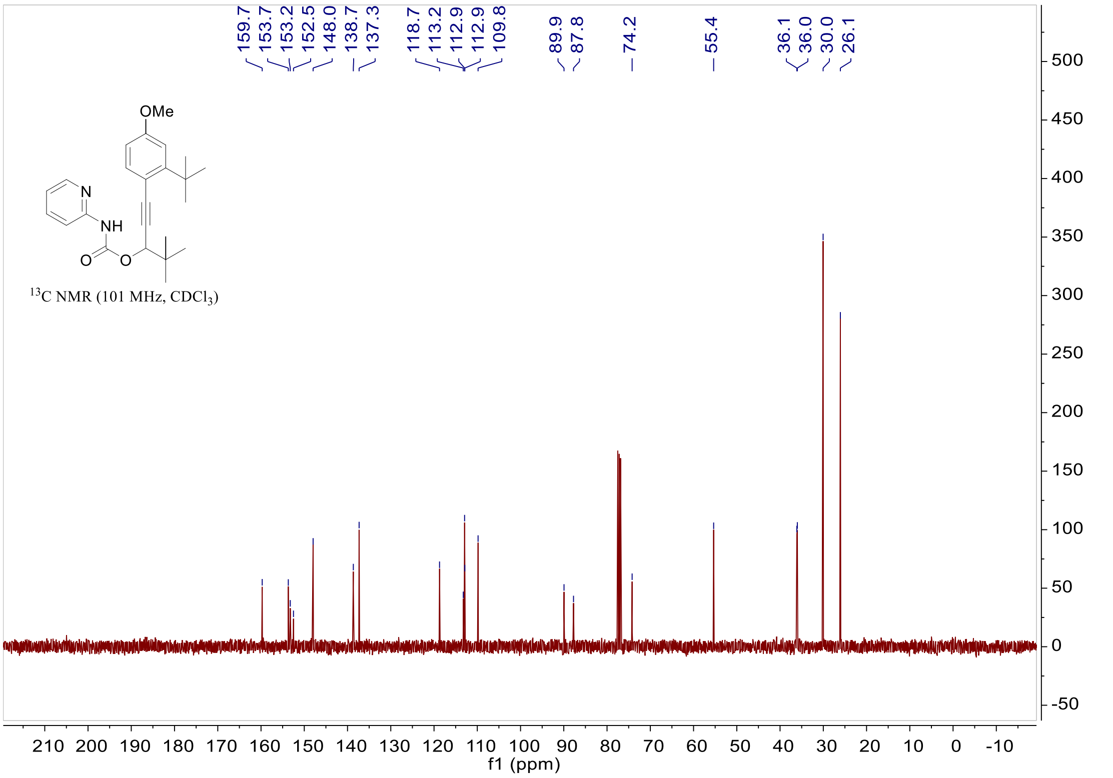
159.7
153.7
153.2
152.5
148.0
138.7
137.3
118.7
113.2
112.9
112.9
109.8
89.9
87.8
74.2
55.4
36.1
36.0
30.0
26.1
OMe
N
NH
O
O
^{13}C NMR (101 MHz, CDCl$_3$)
f1 (ppm)

Compound **2n**

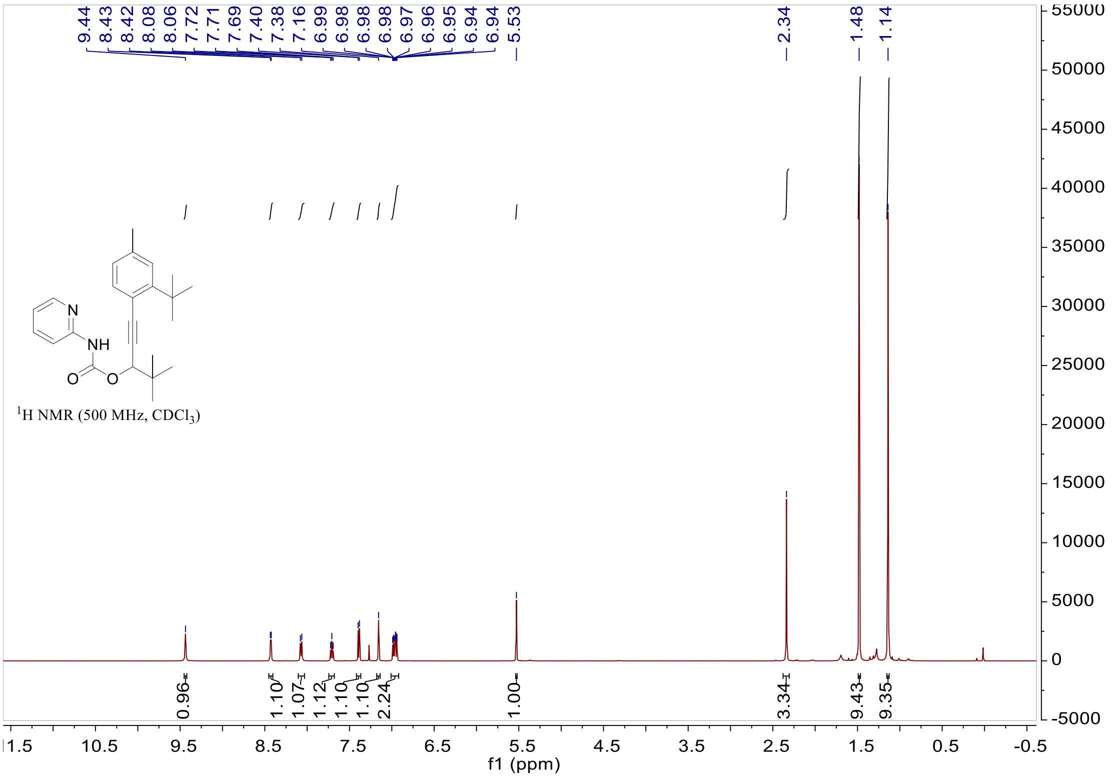

9.44
8.43
8.42
8.08
8.06
7.72
7.71
7.69
7.40
7.38
7.16
6.99
6.98
6.98
6.98
6.97
6.96
6.95
6.94
6.94
5.53
2.34
1.48
1.14
N
NH
O
O
^{1}H NMR (500 MHz, $CDCl_3$)
0.96
1.10
1.07
1.12
1.10
1.10
2.24
1.00
3.34
9.43
9.35
f1 (ppm)

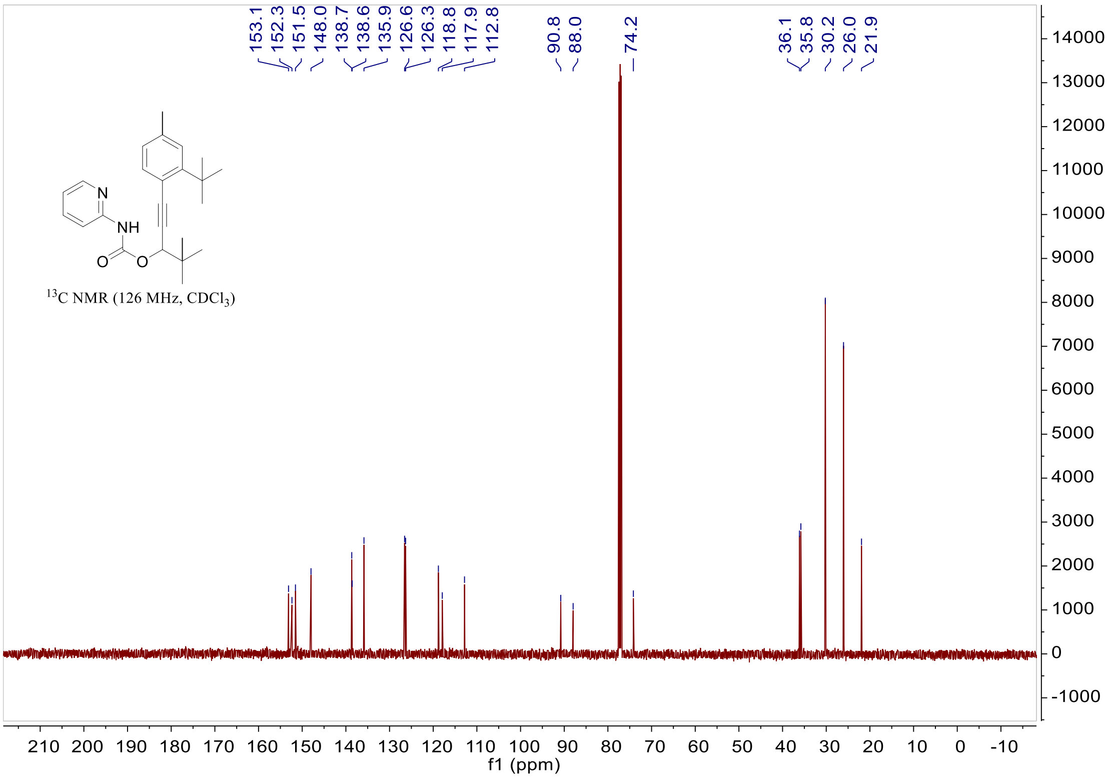

153.1
152.3
151.5
148.0
138.7
138.6
135.9
126.6
126.3
118.8
117.9
112.8
90.8
88.0
74.2
36.1
35.8
30.2
26.0
21.9
N
NH
O
O
^{13}C NMR (126 MHz, $CDCl_3$)
f1 (ppm)

Compound **2o**

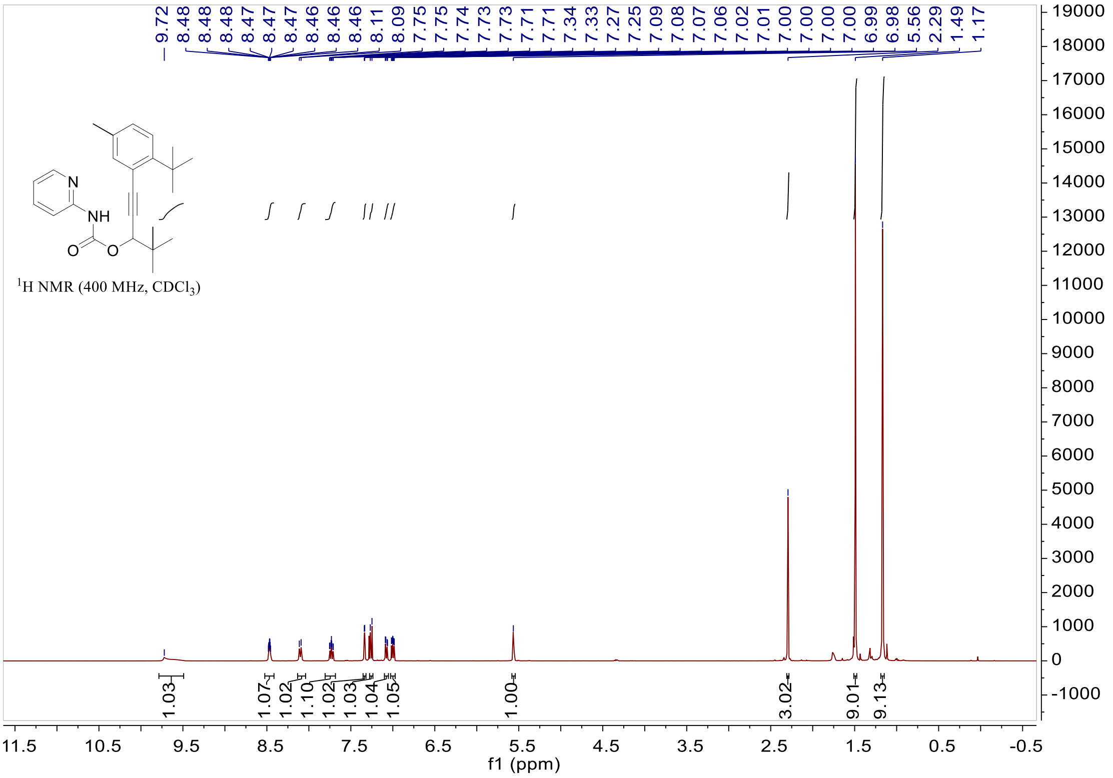

9.72 8.48 8.48 8.48 8.47 8.47 8.47 8.46 8.46 8.11 8.09 7.75 7.75 7.74 7.73 7.73 7.71 7.71 7.34 7.33 7.27 7.25 7.09 7.08 7.07 7.06 7.02 7.01 7.00 7.00 7.00 7.00 6.99 6.98 5.56 2.29 1.49 1.17
N
NH
O
O
^{1}H NMR (400 MHz, CDCl$_3$)
1.03 1.07 1.02 1.10 1.02 1.03 1.04 1.05 1.00 3.02 9.01 9.13
f1 (ppm)

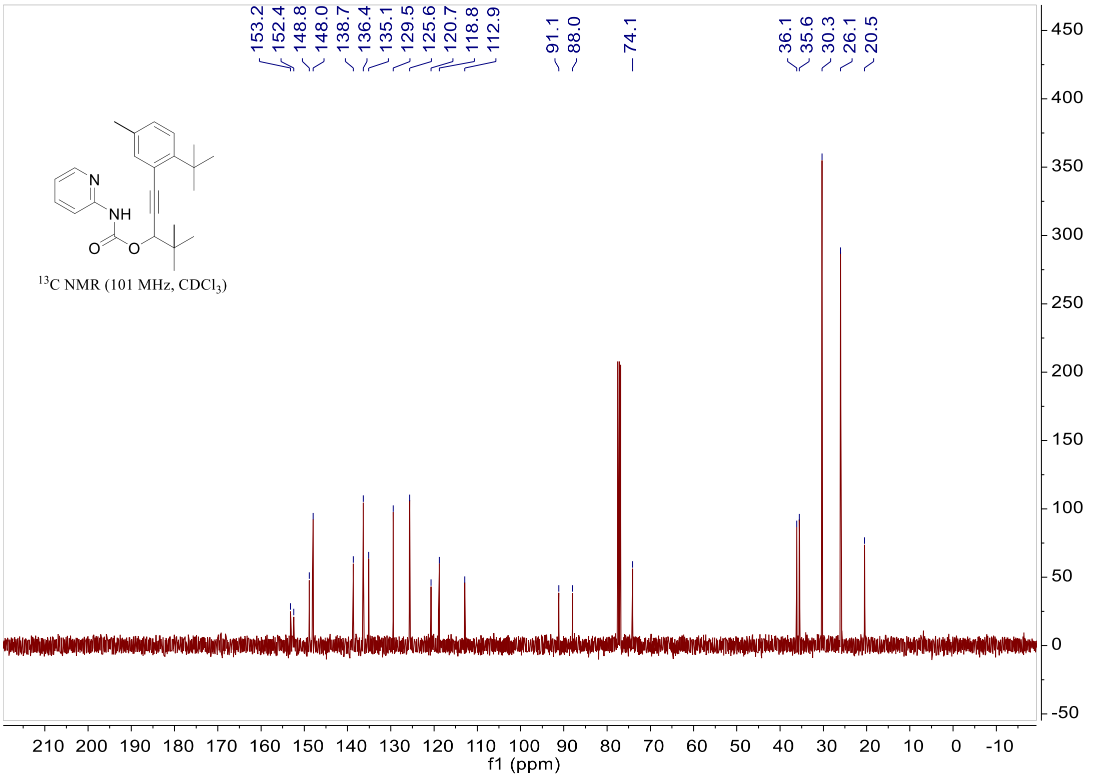

153.2 152.4 148.8 148.0 138.7 136.4 135.1 129.5 125.6 120.7 118.8 112.9 91.1 88.0 74.1 36.1 35.6 30.3 26.1 20.5
N
NH
O
O
^{13}C NMR (101 MHz, CDCl$_3$)
f1 (ppm)

Compound **2p**

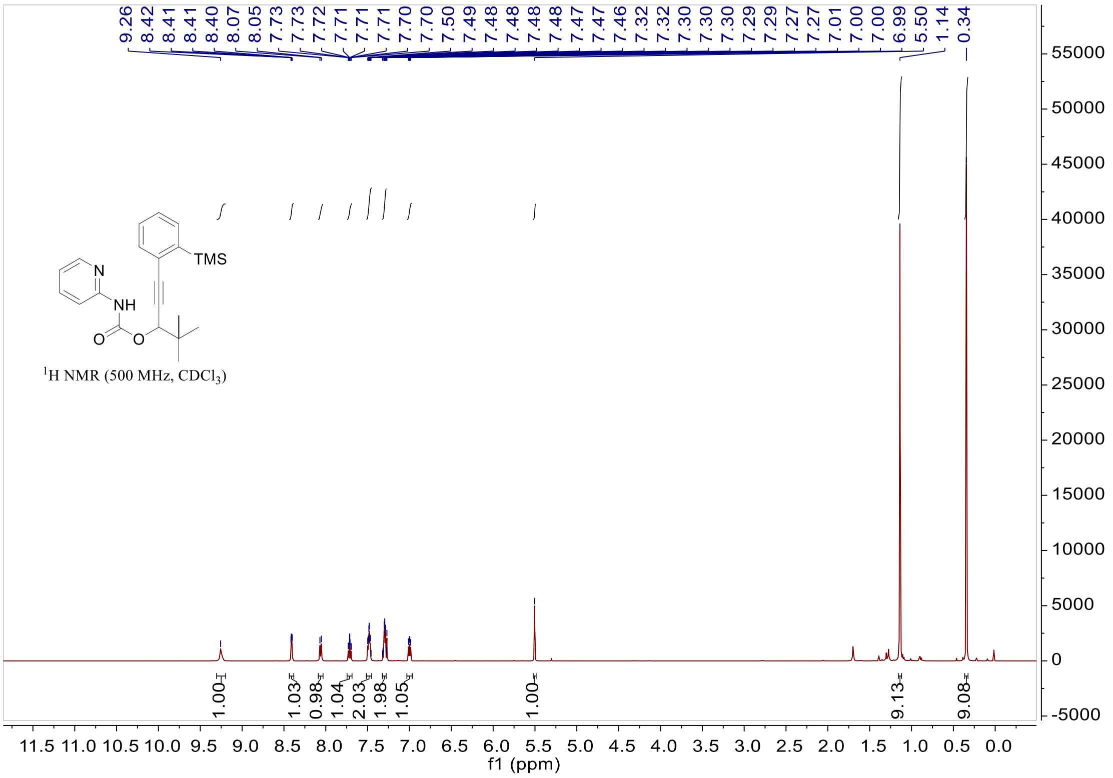
9.26 8.42 8.41 8.40 8.07 8.05 7.73 7.72 7.71 7.71 7.71 7.70 7.70 7.50 7.49 7.48 7.48 7.48 7.48 7.47 7.47 7.46 7.32 7.32 7.30 7.30 7.30 7.29 7.29 7.27 7.27 7.01 7.00 7.00 6.99 5.50 1.14 0.34
1.00 1.03 0.98 1.04 2.03 1.98 1.05 1.00 9.13 9.08
N
NH
O
O
TMS
^{1}H NMR (500 MHz, $CDCl_3$)
f1 (ppm)

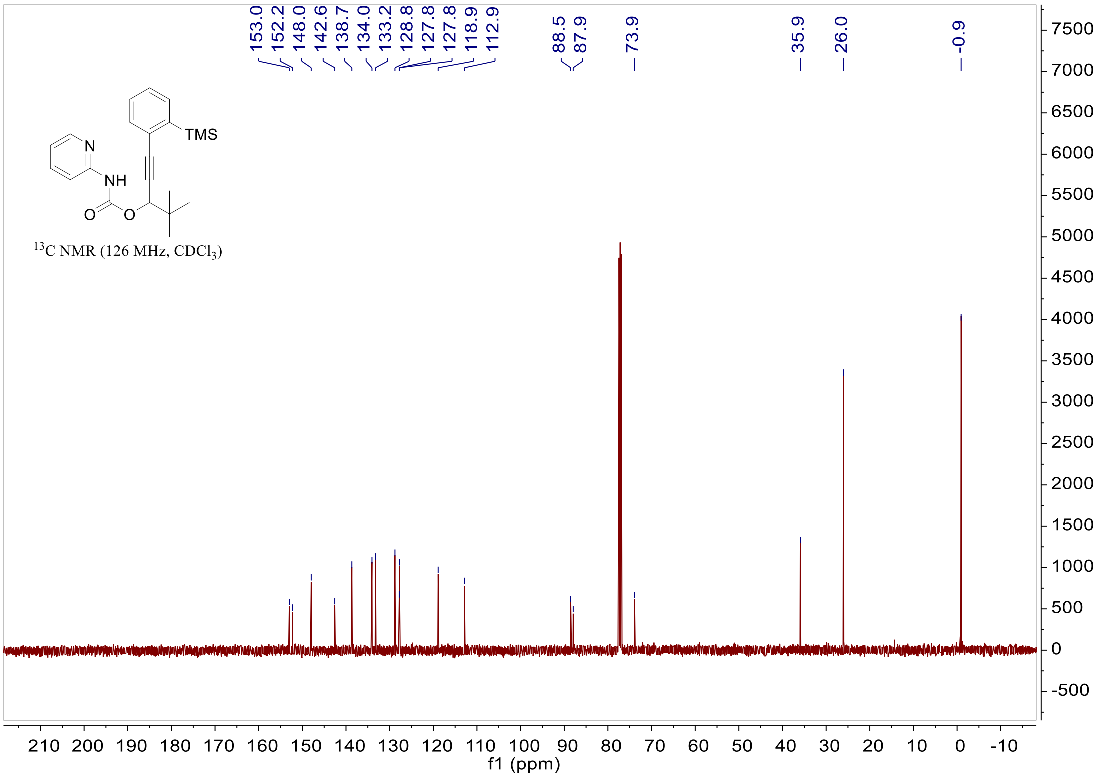
153.0 152.2 148.0 142.6 138.7 134.0 133.2 128.8 127.8 127.8 118.9 112.9 88.5 87.9 73.9 35.9 26.0 -0.9
N
NH
O
O
TMS
^{13}C NMR (126 MHz, $CDCl_3$)
f1 (ppm)

Compound **2q**

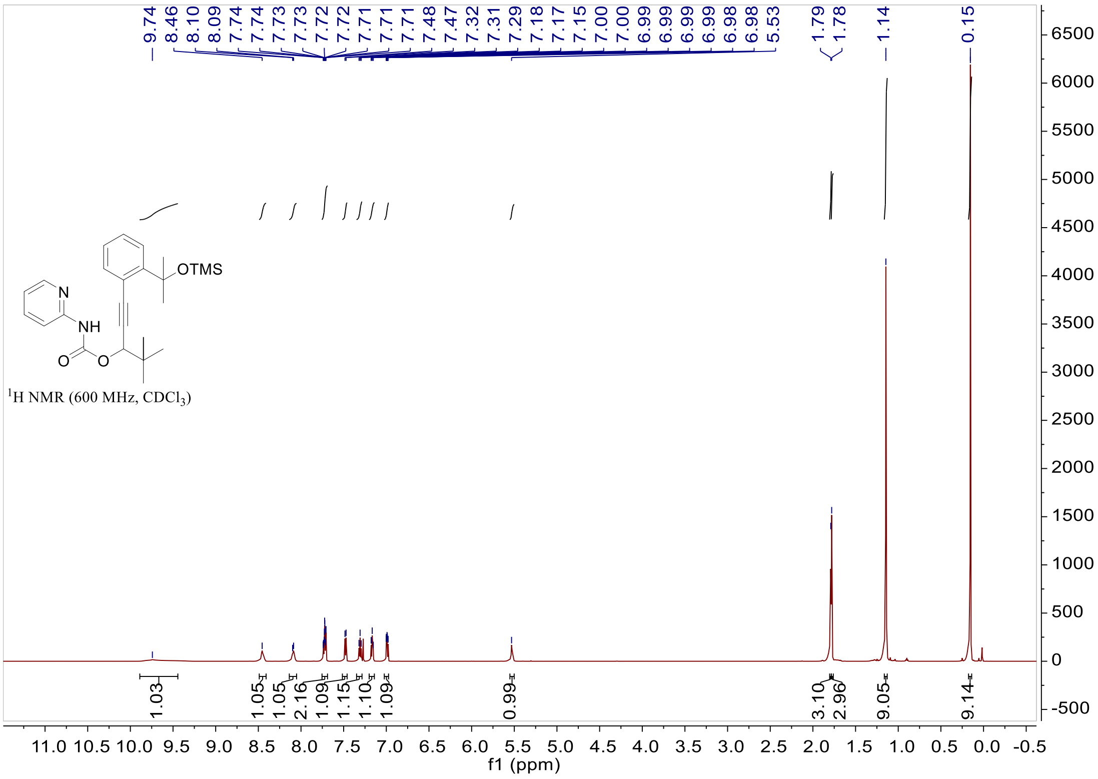

OTMS
N
NH
O
O
^{1}H NMR (600 MHz, CDCl$_3$)
9.74
8.46
8.10
8.09
7.74
7.74
7.73
7.72
7.72
7.71
7.71
7.71
7.48
7.47
7.32
7.31
7.29
7.18
7.17
7.15
7.00
7.00
6.99
6.99
6.99
6.98
6.98
5.53
1.79
1.78
1.14
0.15
1.03
1.05
1.05
2.16
1.09
1.15
1.10
1.09
0.99
3.10
2.96
9.05
9.14
f1 (ppm)

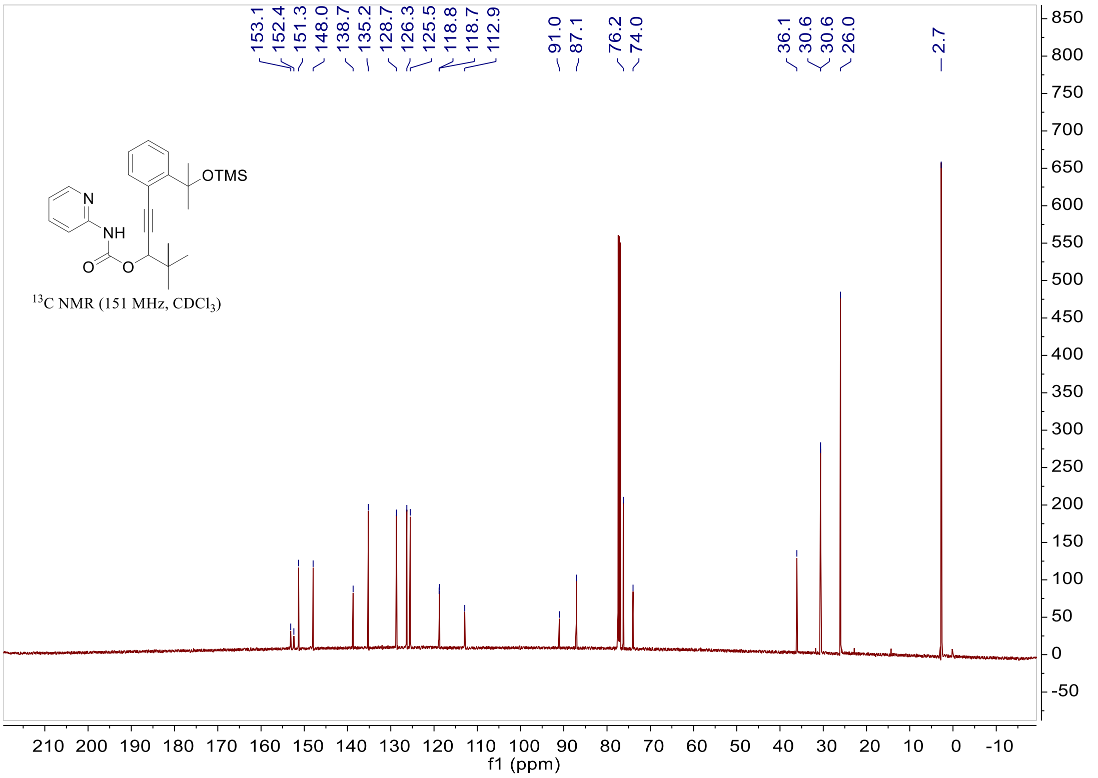

OTMS
N
NH
O
O
^{13}C NMR (151 MHz, CDCl$_3$)
153.1
152.4
151.3
148.0
138.7
135.2
128.7
126.3
125.5
118.8
118.7
112.9
91.0
87.1
76.2
74.0
36.1
30.6
30.6
26.0
2.7
f1 (ppm)

Compound **2r**

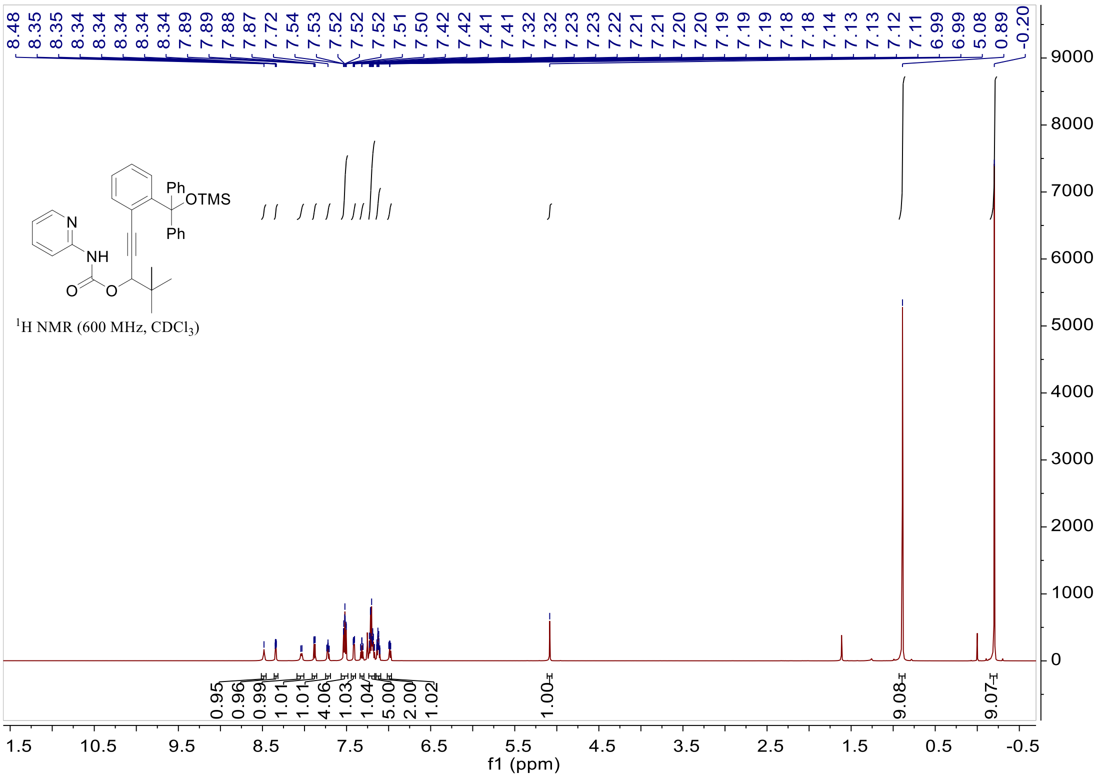
8.48 8.35 8.35 8.34 8.34 8.34 8.34 8.34 7.89 7.89 7.88 7.87 7.72 7.54 7.53 7.52 7.52 7.52 7.51 7.50 7.42 7.42 7.41 7.41 7.32 7.32 7.23 7.23 7.22 7.21 7.21 7.20 7.20 7.19 7.19 7.19 7.18 7.18 7.14 7.13 7.13 7.12 7.11 6.99 6.99 5.08 0.89 -0.20
Ph
OTMS
Ph
N
NH
O
O
¹H NMR (600 MHz, CDCl₃)
0.95 0.96 0.99 1.01 1.01 4.06 1.03 1.04 5.00 2.00 1.02
1.00
9.08
9.07
f1 (ppm)

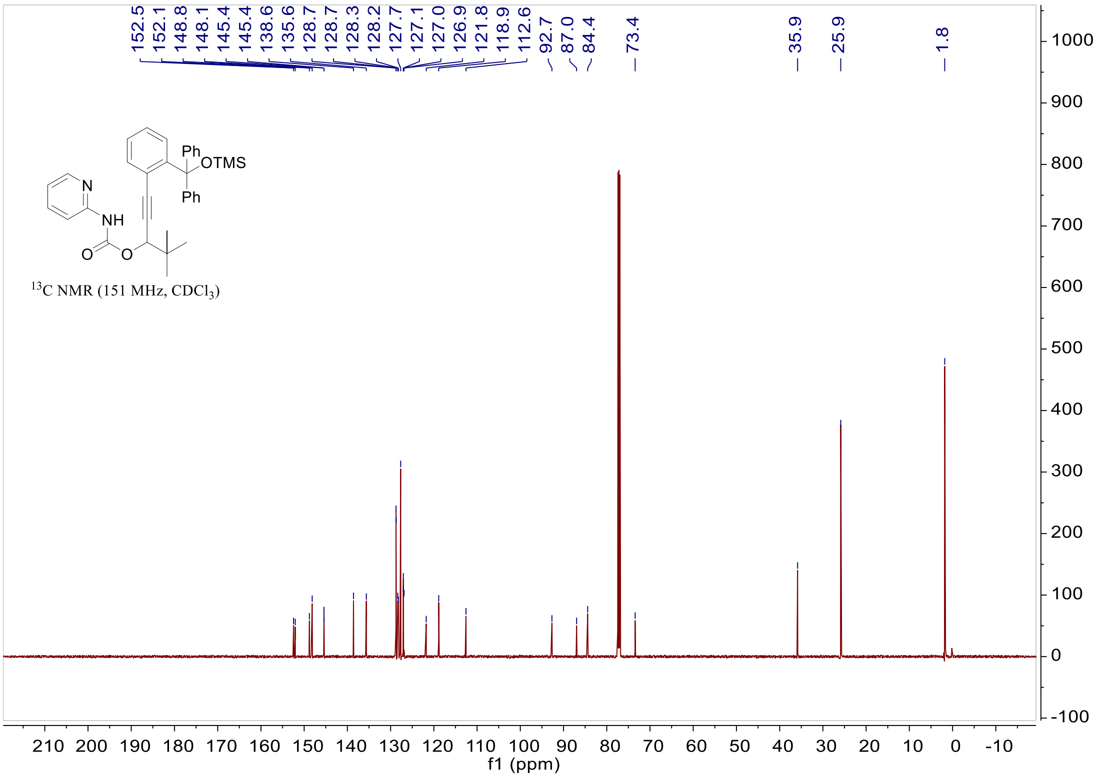
152.5 152.1 148.8 148.1 145.4 145.4 138.6 135.6 128.7 128.7 128.3 128.2 127.7 127.1 127.0 126.9 121.8 118.9 112.6
92.7 87.0 84.4
73.4
35.9
25.9
1.8
Ph
OTMS
Ph
N
NH
O
O
¹³C NMR (151 MHz, CDCl₃)
f1 (ppm)

Compound **2s**

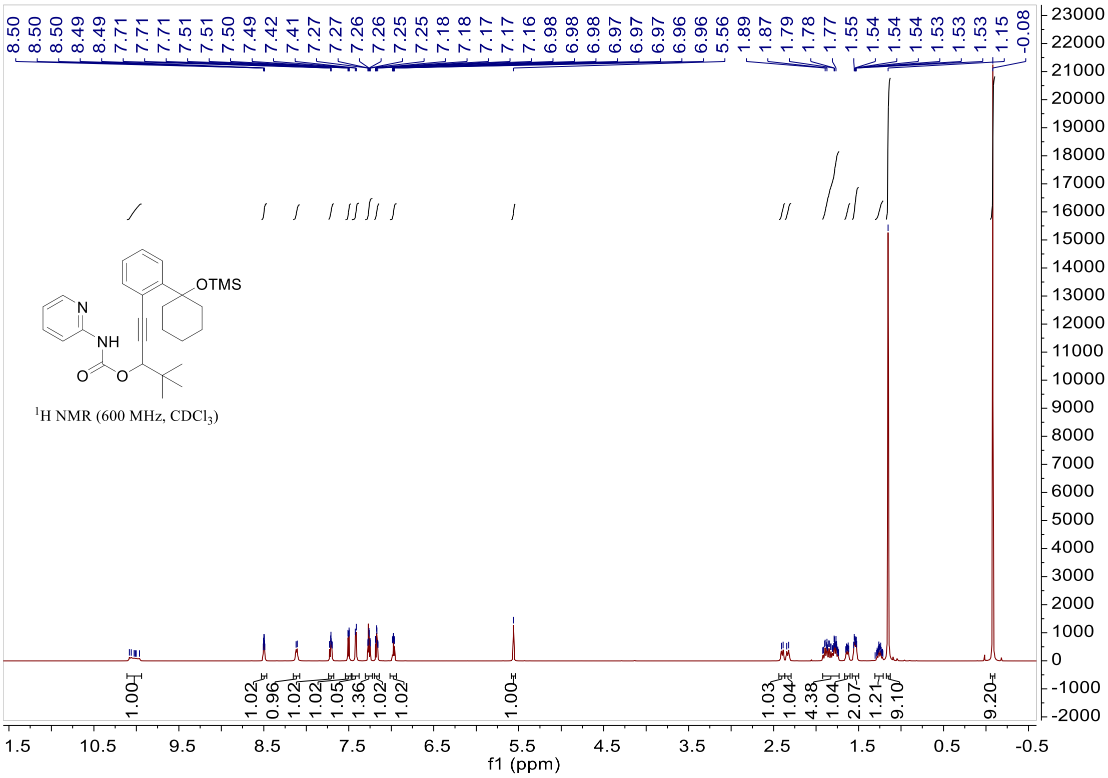

8.50
8.50
8.50
8.49
8.49
7.71
7.71
7.71
7.71
7.51
7.51
7.50
7.49
7.42
7.41
7.27
7.27
7.26
7.26
7.25
7.25
7.18
7.18
7.17
7.17
7.16
6.98
6.98
6.98
6.97
6.97
6.96
6.96
5.56
1.89
1.87
1.79
1.78
1.77
1.55
1.54
1.54
1.54
1.53
1.53
1.53
1.15
-0.08
OTMS
N
NH
O
O
^{1}H NMR (600 MHz, CDCl$_3$)
1.00
1.02
0.96
1.02
1.02
1.05
1.36
1.02
1.02
1.00
1.03
1.04
4.38
1.04
2.07
1.21
9.10
9.20
f1 (ppm)

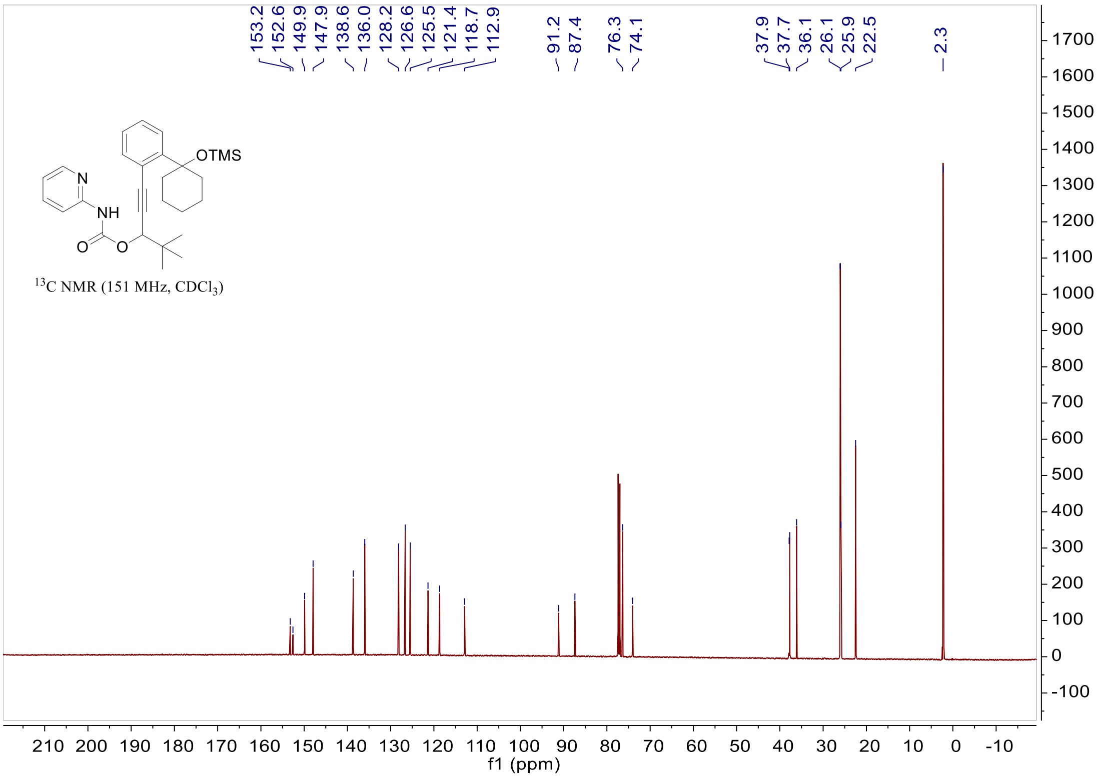

153.2
152.6
149.9
147.9
138.6
136.0
128.2
126.6
125.5
121.4
118.7
112.9
91.2
87.4
76.3
74.1
37.9
37.7
36.1
26.1
25.9
22.5
2.3
OTMS
N
NH
O
O
^{13}C NMR (151 MHz, CDCl$_3$)
f1 (ppm)

Compound **2t**

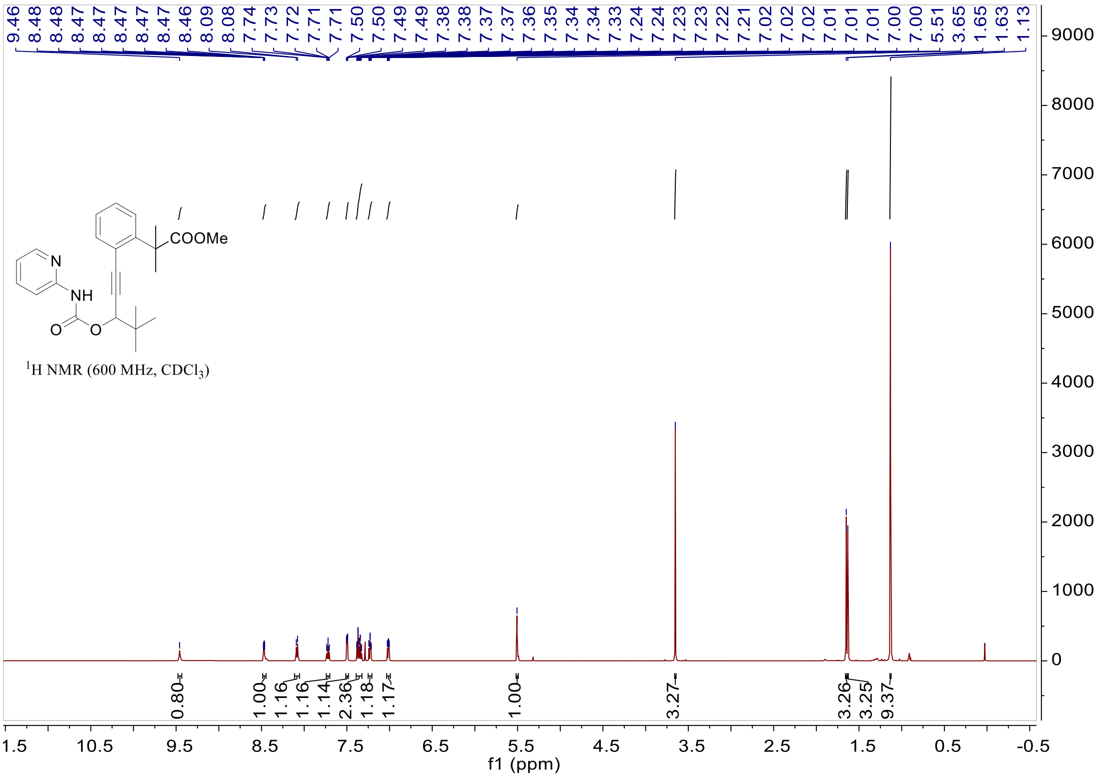
9.46
8.48
8.48
8.47
8.47
8.47
8.47
8.47
8.46
8.09
8.08
7.74
7.73
7.72
7.71
7.71
7.50
7.50
7.49
7.49
7.38
7.37
7.37
7.36
7.35
7.34
7.34
7.33
7.24
7.24
7.23
7.23
7.22
7.21
7.02
7.02
7.02
7.01
7.01
7.00
7.00
5.51
3.65
1.65
1.63
1.13
COOMe
N
NH
O
O
¹H NMR (600 MHz, CDCl₃)
0.80
1.00
1.16
1.16
1.14
2.36
1.18
1.17
1.00
3.27
3.26
3.25
9.37
f1 (ppm)

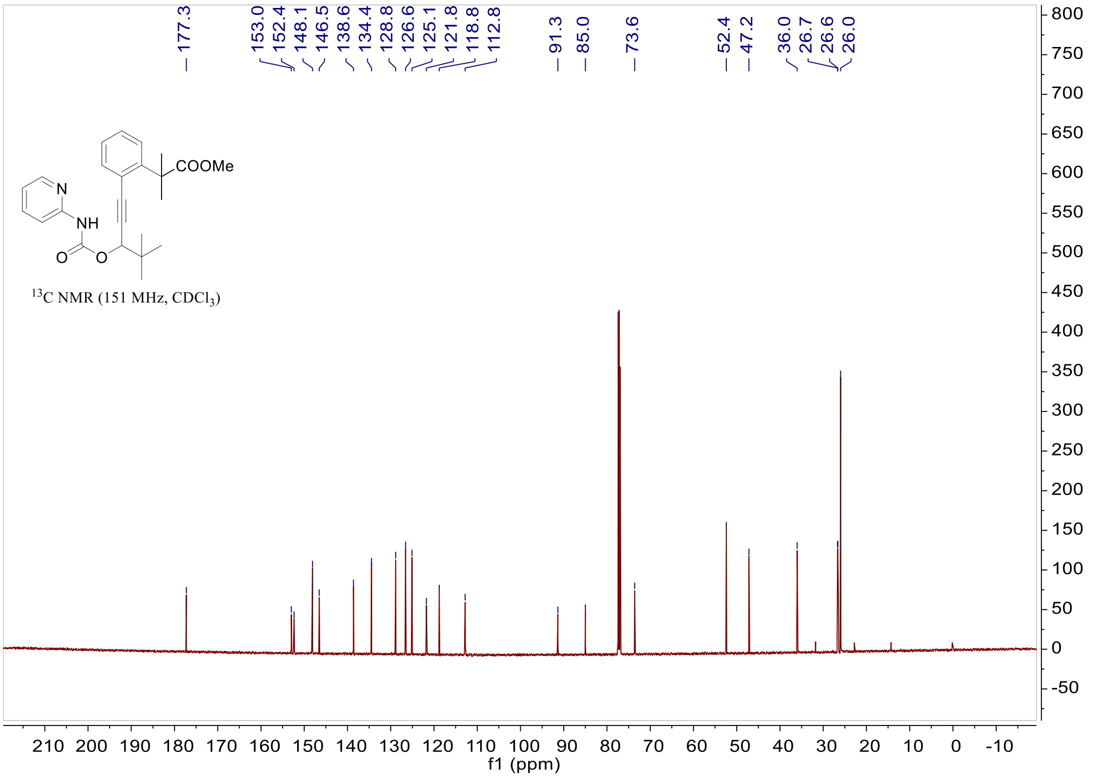
177.3
153.0
152.4
148.1
146.5
138.6
134.4
128.8
126.6
125.1
121.8
118.8
112.8
91.3
85.0
73.6
52.4
47.2
36.0
26.7
26.6
26.0
COOMe
N
NH
O
O
¹³C NMR (151 MHz, CDCl₃)
f1 (ppm)

Compound **2u**

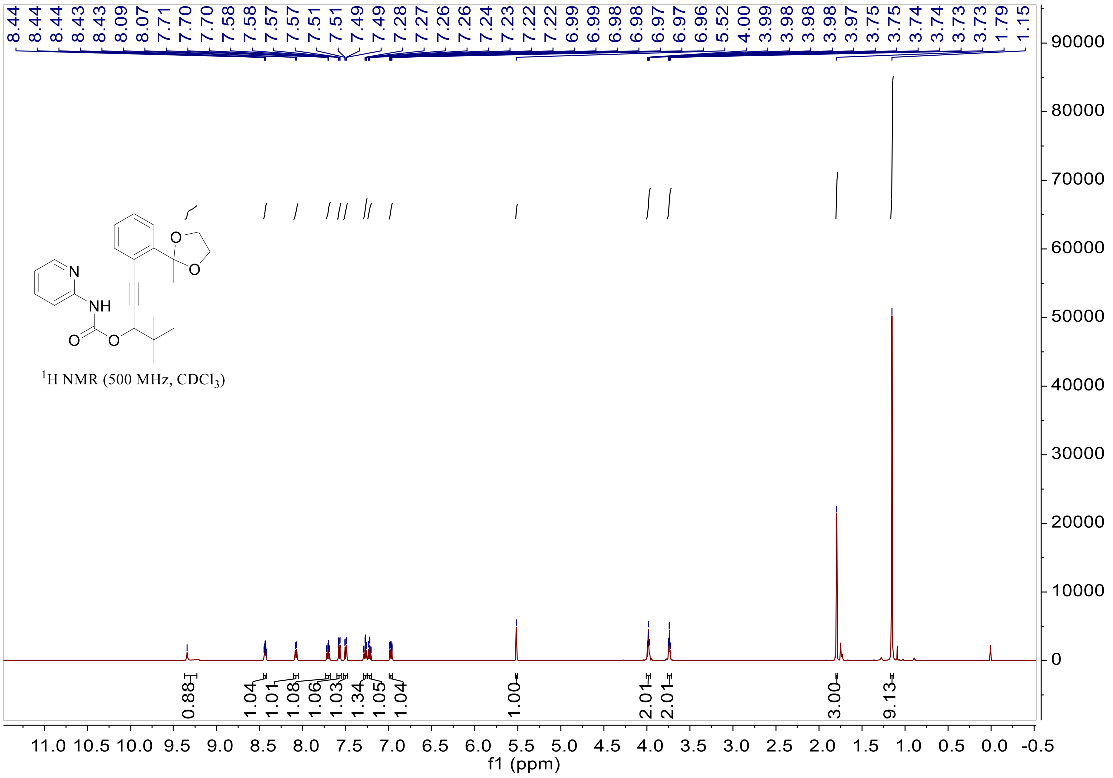

^{1}H NMR (500 MHz, $CDCl_3$)

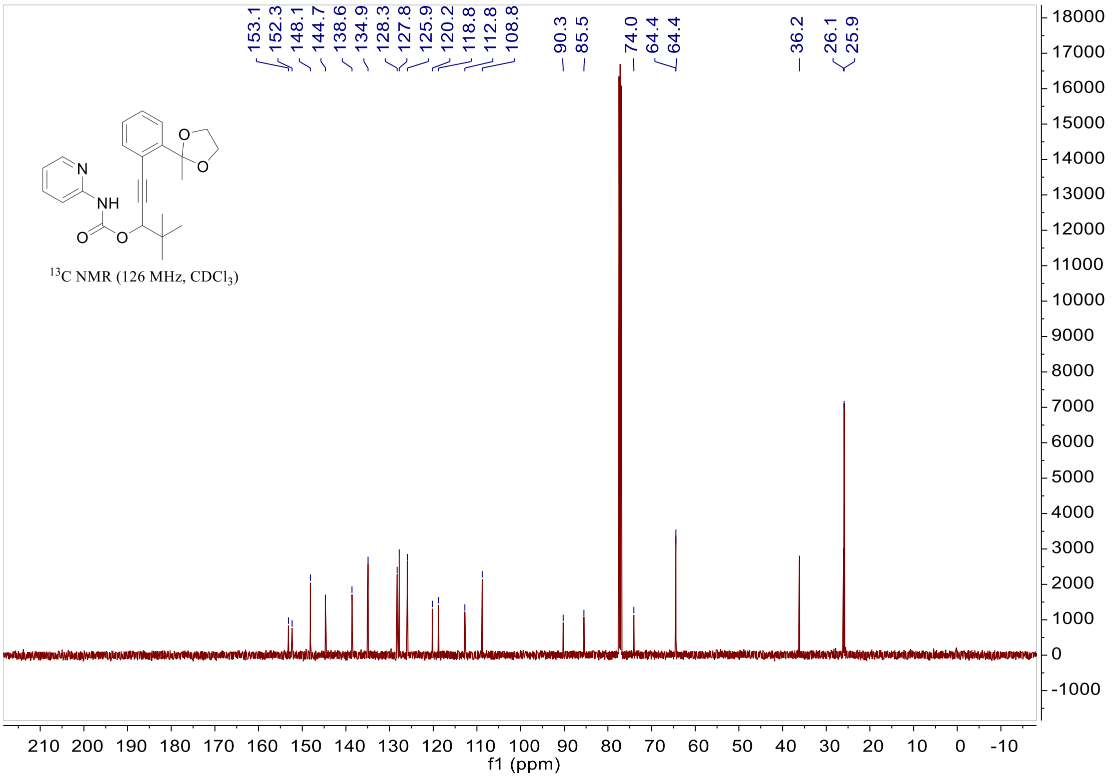

^{13}C NMR (126 MHz, $CDCl_3$)

Compound **2v**

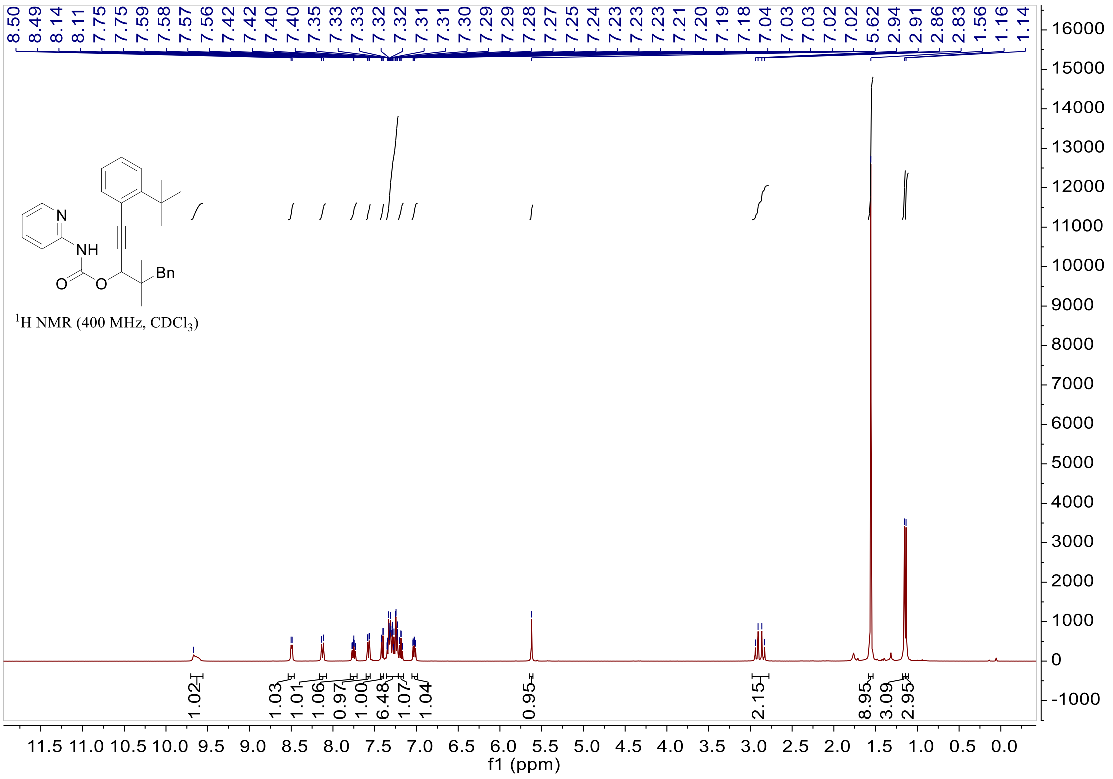

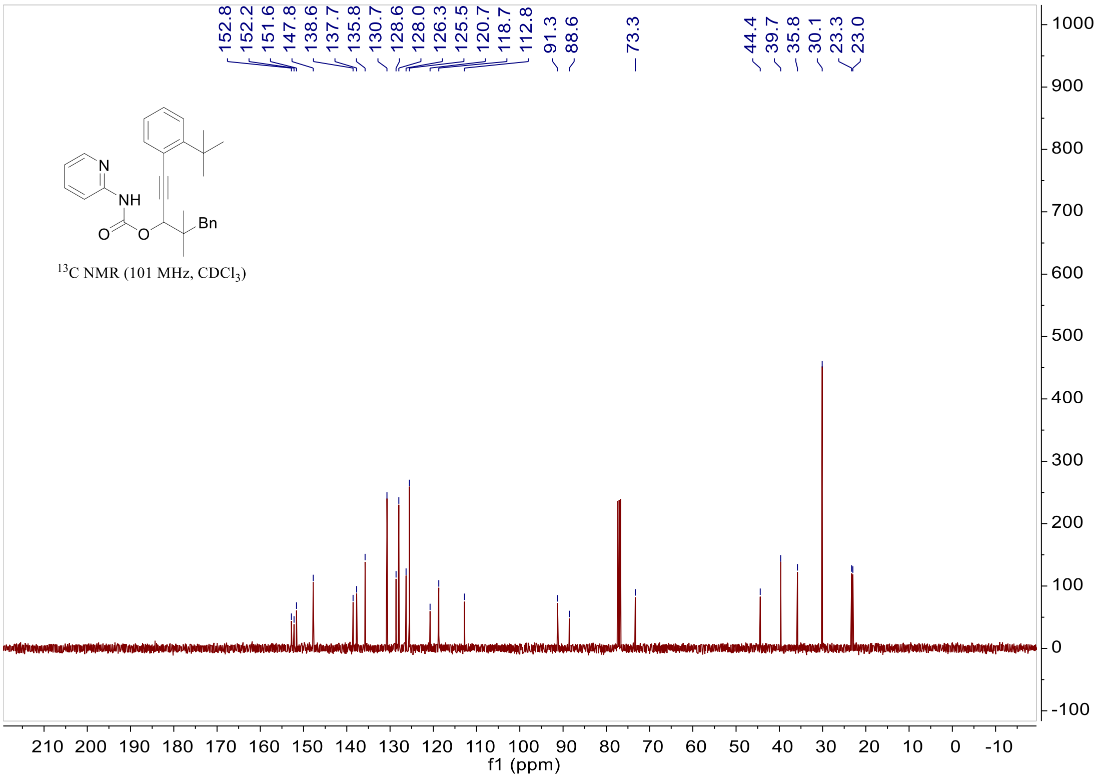

Compound **2w**

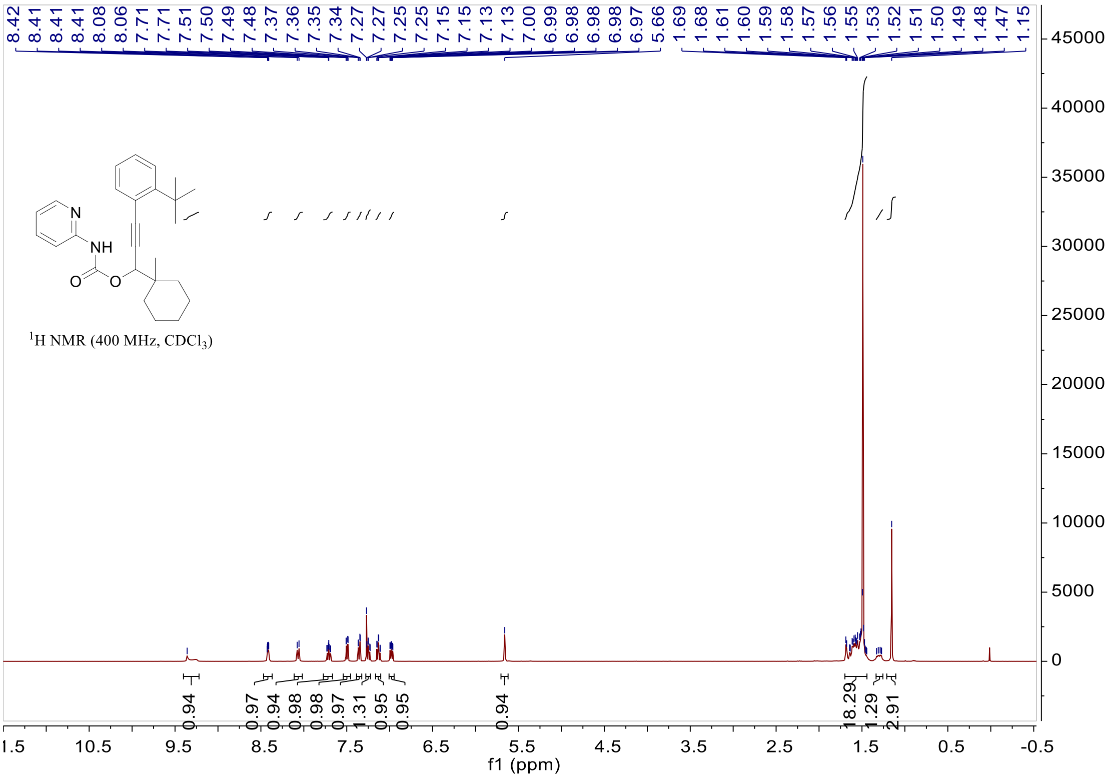

NH
N
O
O
^{1}H NMR (400 MHz, CDCl$_3$)
8.42
8.41
8.41
8.08
8.06
7.71
7.71
7.51
7.50
7.49
7.48
7.37
7.36
7.35
7.34
7.27
7.27
7.25
7.25
7.15
7.15
7.13
7.13
7.00
6.99
6.98
6.98
6.98
6.97
5.66
1.69
1.68
1.61
1.60
1.59
1.58
1.57
1.56
1.55
1.53
1.52
1.51
1.50
1.49
1.48
1.47
1.15
0.94
0.97
0.94
0.98
0.98
0.97
1.31
0.95
0.95
0.94
18.29
1.29
2.91
f1 (ppm)

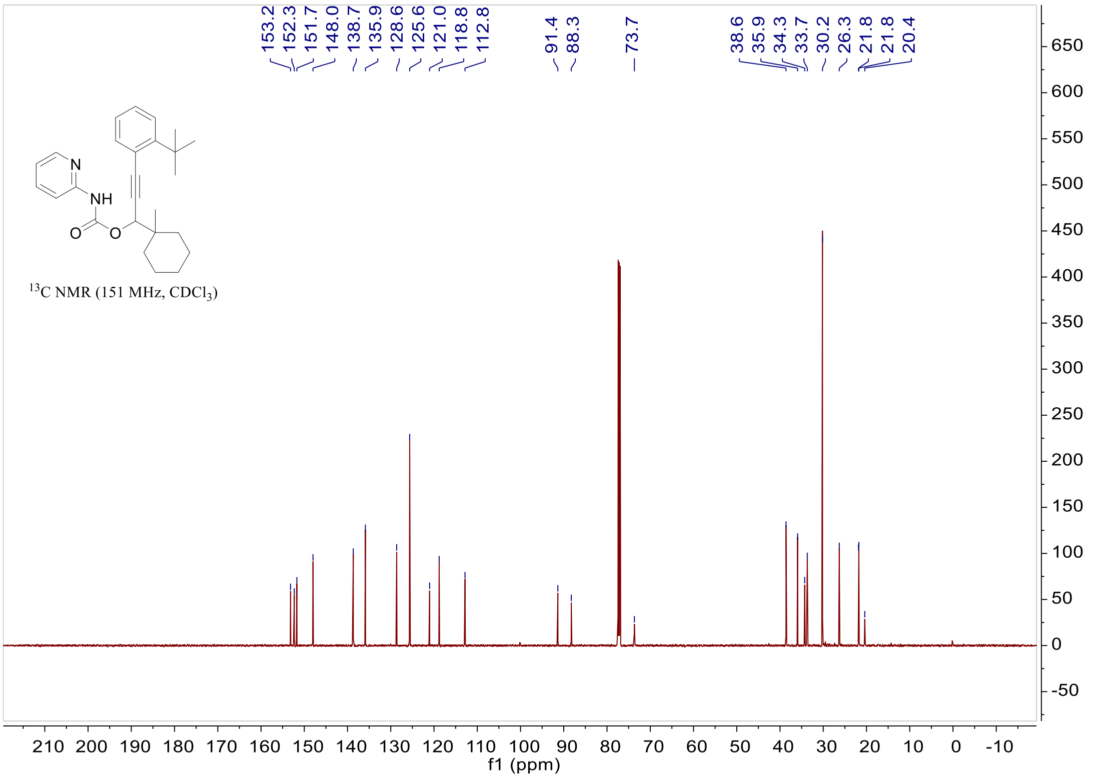

NH
N
O
O
^{13}C NMR (151 MHz, CDCl$_3$)
153.2
152.3
151.7
148.0
138.7
135.9
128.6
125.6
121.0
118.8
112.8
91.4
88.3
73.7
38.6
35.9
34.3
33.7
30.2
26.3
21.8
21.8
20.4
f1 (ppm)
